# Effectiveness, immunogenicity and safety of human papillomavirus vaccination in non-HIV immunocompromised individuals: a systematic review

**DOI:** 10.1016/j.eclinm.2026.103865

**Published:** 2026-04-11

**Authors:** Philipp Kapp, Waldemar Siemens, Lea Gorenflo, Henriette Schulz, Yuan Chi, Marianne Röbl-Mathieu, Mona Askar, María Brotons, Peter Henrik Andersen, Deborah Konopnicki, Judi Lynch, Simona Ruţă, Liisa Saare, Béatrice Swennen, Ruth Tachezy, Anja Takla, Veronika Učakar, Simopekka Vänskä, Dace Zavadska, Karam Adel Ali, Kate Olsson, Thomas Harder, Joerg J. Meerpohl

**Affiliations:** a Institute for Evidence in Medicine, Medical Center - University of Freiburg/Medical Faculty - University of Freiburg, Freiburg, Germany; b Cochrane Germany, Cochrane Germany Foundation, Freiburg, Germany; c Department of Cardiology, The First Affiliated Hospital, Jinan University, Guangzhou, China; d Gynaecologist's office, Munich, Germany; e Standing Committee on Vaccination at the Robert Koch Institute, Berlin, Germany; f Immunization Unit, Robert Koch-Institute, Berlin, Germany; g Cancer Epidemiology Research Programme, Catalan Institute of Oncology (ICO), L'Hospitalet de Llobregat, Barcelona, Spain; h Bellvitge Biomedical Research Institute – IDIBELL. L'Hospitalet de Llobregat, Barcelona, Spain; i Consortium for Biomedical Research in Epidemiology and Public Health – CIBERESP, Madrid, Spain; j Department of Infectious Disease Epidemiology and Prevention, SSI, Copenhagen, Denmark; k Saint-Pierre University Hospital, Université Libre de Bruxelles, Brussels, Belgium; l National Cervical Screening Laboratory, The Coombe Hospital, Dublin, Ireland; m Carol Davila University of Medicine and Pharmacy & Stefan S. Nicolau Institute of Virology, Bucharest, Romania; n Institute of Family Medicine and Public Health, University of Tartu, Tartu, Estonia; o Public Health School, Université Libre de Bruxelles, Brussels, Belgium; p Faculty of Science BIOCEV Charles University, Prague, Czech Republic; q National Institute of Public Health, Ljubljana, Slovenia; r Finnish Institute for Health and Welfare, Helsinki, Finland; s Rīgas Stradiņa Universitāte, Riga, Latvia; t European Centre for Disease Prevention and Control, Stockholm, Sweden

**Keywords:** Human papilloma virus, Immunocompromised, Cervical cancer, Immunogenicity, Vaccination

## Abstract

**Background:**

Immunocompromised individuals may be at an increased risk for human papillomavirus (HPV)-related diseases and cancers, but the protective benefit of HPV vaccination remains unclear. In this systematic review and meta-analysis we assessed the efficacy, effectiveness, immunogenicity, and safety of HPV vaccination in non-HIV immunocompromised individuals.

**Methods:**

We searched MEDLINE, Embase and CENTRAL (26 November 2025) for randomised and non-randomised studies comparing vaccinated immunocompromised individuals to unvaccinated immunocompromised individuals (comparison 1). Additionally, we considered studies comparing vaccinated immunocompromised individuals to vaccinated individuals with a different disease or condition (comparison 2) or vaccinated healthy individuals (comparison 3). We assessed the risk of bias (ROBINS-I) and the certainty of evidence (CoE; GRADE) for prioritised outcomes including cervical precancer or cancer, HPV types 16 and 18 immunogenicity, and serious adverse events. We pooled studies using the random-effect meta-analyses model. This study is registered in PROSPERO, CRD42024554574.

**Findings:**

We identified 24 non-randomised studies, comprising various immunocompromised populations (e.g. solid organ transplant recipients or autoimmune diseases). One case–control study compared vaccinated immunocompromised with unvaccinated immunocompromised individuals (comparison 1), reporting rate ratios near the null effect for effectiveness against cervical intraepithelial neoplasia (CIN) 2+ (0.96, 95% CI 0.68–1.37) and CIN 3+ (0.96, 95% CI 0.54–1.70), although the CoE was very low. Most studies assessed immunogenicity, generally showing high seropositivity rates (median at 7 months 95.8%, IQR 89.2–99.7; 13 studies) in immunocompromised individuals compared to other immunocompromised or healthy individuals, with a CoE ranging from low to very low (comparisons 2 and 3). Antibody titres were generally high but varied across immunocompromised populations. Serious adverse events were rare and deemed unrelated to vaccination.

**Interpretation:**

HPV vaccination appears immunogenic and safe for non-HIV immunocompromised individuals, but the CoE is low to very low and heterogeneity across populations limits generalisability of the findings and pooled analyses. Given the unclear correlate of protection and lack of standardisation of assays for antibody measurement, immunogenicity data should be interpreted cautiously. Future studies should assess HPV-associated precancerous lesions and cancers, and explore subgroups effects, including differences in sex, age, immunosuppressive treatments, and dosing.

**Funding:**

10.13039/100032850EU4Health Programme - European Health and Digital Executive Agency, European Commission. Service Contract HaDEA/OP/2021/0011.

**Research in context**

**Evidence before this study**

We conducted a systematic literature search on 26 November 2025, across Ovid MEDLINE, Ovid Embase, Cochrane Central, and ClinicalTrials.gov, supplemented by reference screening of included studies and prior systematic reviews.

In our literature search, we identified three systematic reviews focussing on non-HIV immunocompromised populations, each including three to six non-randomised studies of interventions (NRSI). These reviews mainly concentrated on specific immunocompromised groups, such as solid organ transplant recipients, and only one conducted meta-analyses. No comprehensive review has evaluated human papillomavirus (HPV) vaccination across a broader spectrum of non-HIV immunocompromised groups. Although HPV vaccination shows beneficial effects in the general population, its protective effects in immunocompromised populations beyond HIV, such as solid organ transplant recipients or individuals with autoimmune diseases, remain uncertain despite their potentially increased risk of HPV-related diseases.

**Added value of this study**

Addressing the evidence gap for non-HIV immunocompromised populations, this systematic review and meta-analysis evaluated HPV vaccine effectiveness, immunogenicity, and safety in this population. We identified 24 NRSI and 15 registry entries encompassing diverse immunocompromised groups. Only one case–control study reported on vaccine effectiveness outcomes (i.e. cervical intraepithelial neoplasia [CIN] 2+ and CIN 3+) without showing clear protective effects. Most studies reported immunogenicity data showing generally high seropositivity rates, similar to those observed in healthy populations at ≥7 months follow-up. Antibody titres were elevated in general but varied across immunocompromised populations. Serious adverse events were rare and deemed unrelated to vaccination. In summary, HPV vaccination appears immunogenic and safe for non-HIV immunocompromised individuals, but the certainty of evidence is low to very low and heterogeneity across populations limits generalisability of the findings and pooled analyses.

**Implications of all the available evidence**

Identified studies are mostly small, non-randomised and deficient in terms of follow-up time and clinically relevant outcomes. Most studies reported data on the quadrivalent HPV vaccine, while few studies evaluated bivalent or nonavalent vaccines. The interpretation of immunogenicity data is further limited by unclear correlates of protection and heterogeneity in antibody assays. High-quality studies are needed, ideally randomised controlled trials, but also well-designed observational studies, comparing vaccinated and unvaccinated immunocompromised individuals. Future research should also distinguish between the variety of immunocompromised groups and subgroups, including differences in specific immunosuppressive treatments, dosing, age and sex.

## Introduction

Cervical cancer is the fourth most common cancer affecting women globally, caused by persistent infection with oncogenic types, also known as high-risk HPV types, of human papillomavirus (HPV). Of over 200 identified HPV types, more than 40 infect the genital tract. HPV types 16 and 18 are the two primary oncogenic types, and are responsible for 77% of cervical cancers (i.e. squamous cell carcinoma) and together with HPV types 31, 33, 45, 52 and 58 account for 95% of cervical cancers globally.^1^, ^2^, ^3^ While most HPV infections resolve spontaneously, persistent infections with high-risk HPV types can progress to premalignant glandular or squamous intraepithelial lesions (cervical dysplasia). High risk HPV types are also associated with different cancers, e.g. of the anus, vagina, vulva, penis and oropharynx. In addition to the oncogenic types, studies indicate that HPV type 6 and 11 are the primary causes of anogenital warts.^2^

HPV vaccination in adolescents is a key measure to prevent HPV-associated cancers.^4^^,^^5^ To date, most HPV vaccination programmes target adolescent girls and boys, while some countries have extended HPV vaccination catch-up programmes to adults.^6^ In Europe, three vaccines, bivalent, quadrivalent, and nonavalent, are currently approved, all covering types 16 and 18. The nonavalent also includes the types 31, 33, 45, 52, and 58. The non-oncogenic types 6 and 11 are also covered by the nonavalent and the quadrivalent vaccines.^7^

Although HPV vaccination shows beneficial effects in the general population, its protective benefit in immunocompromised individuals, including solid organ transplant recipients, cancer patients and individuals with autoimmune diseases, remains unclear. While this heterogeneous group is at a potentially higher risk for HPV related disease, such as cervical cancers, the vaccine-induced immune responses may be altered by the underlying diseases, immunomodulatory treatments, and demographic factors.^8^, ^9^, ^10^, ^11^, ^12^, ^13^, ^14^, ^15^ Estimates from the United States and England suggest that 2.7%–6.6% of the adult population are immunocompromised.^16^, ^17^, ^18^

While existing evidence syntheses on HPV vaccination in immunocompromised individuals primarily focus on populations with HIV,^19^, ^20^, ^21^ there is no comprehensive systematic review on other immunocompromised populations.^22^, ^23^, ^24^, ^25^

This systematic review and meta-analysis aims to investigate the efficacy, effectiveness, immunogenicity, and safety of HPV vaccination in non-HIV immunocompromised individuals, with a focus on cervical cancer and precancer, and immunogenicity-related outcomes.

## Methods

We reported this systematic review and meta-analysis in accordance with the standards of the Preferred Reporting Items for Systematic Review and Meta-Analysis (PRISMA) Statement This study is registered in PROSPERO, CRD42024554574.^26^

### Search strategy and selection criteria

We conducted the systematic search on 26 November 2025 in three electronic databases: Ovid MEDLINE, Ovid Embase and the Cochrane Central Register of Controlled Trials (CENTRAL; Supplement Table S1–S3). A second information specialist peer-reviewed the literature search, following the recommendations of PRESS (Peer Review of Electronic Search Strategies).^27^ We conducted a supplementary search in ClinicalTrials.gov to identify ongoing studies or unpublished completed studies (Supplement Table S4). Supplementary searches covered reference lists of relevant studies, systematic reviews and the websites of regulatory agencies (European Medicines Agency and Food and Drug Administration). We did not use date or language restrictions. We included full-text journal publications and preprint articles. We excluded studies reported in abstract form only, theses, editorials, letters and comments, due to potentially limited information on study methods.

To assess the efficacy, effectiveness and safety, we searched for randomised controlled trials (RCTs), as this study design, if performed appropriately and under ideal conditions of clinical care, provides the best evidence for efficacy questions. In addition, we included non-randomised studies of interventions (NRSI), defined as studies in which participants are allocated to different groups using methods that are not random and observational studies. In observational studies, group allocation is determined by factors outside the investigator's control, allowing the assessment of the HPV vaccine's effectiveness under typical clinical care conditions.^28^^,^^29^ We also included single-arm studies to assess vaccine safety. Furthermore, we included studies investigating immunocompromised individuals of any age and sex, excluding HIV and other infectious diseases, such as malaria or helminthiasis. We included the nonavalent, quadrivalent, and bivalent HPV vaccines. The detailed eligibility criteria of this review are presented in Supplement Table S5.

We included studies that compared vaccinated immunocompromised groups to unvaccinated immunocompromised control groups with the same disease or condition (comparison 1), receiving placebo, a non-HPV vaccine or no vaccination. In addition, we considered studies that compared vaccinated immunocompromised groups to vaccinated control groups with a different disease or condition that affects the immune system (comparison 2) or vaccinated healthy individuals (i.e. participants from the general population) that are not immunocompromised (comparison 3; Supplement Table S5).

Two reviewers independently screened citations (title and abstract screening [PK, WS, LG, AT]; full text screening [PK, WS]) identified in electronic data sources in Covidence (https://www.covidence.org/home). We resolved disagreements by consensus, moderated by a third reviewer, if necessary (LG, JM).

Two review authors (PK, WS, LG, HS) extracted 20% of the study characteristics and outcome data independently, using a customised data extraction form. One reviewer extracted the remaining studies, followed by a second reviewer that verified the data (PK, WS). We solved disagreements by discussion.

We included the following patient-relevant efficacy and effectiveness outcomes irrespective of HPV types and specifically for HPV types 16 and 18: precancer or cancer of the cervix (histopathologically confirmed), precancers or cancers of the vulva, vagina, penis or anus and oropharyngeal cancer, HPV infection and mortality caused by HPV-related cancers. Additionally, we collected studies reporting anogenital warts and immunogenicity parameters for HPV types 16 and 18, including seropositivity rates and geometric mean ratios (GMRs), to assess the antibody responses. We collected safety and adverse outcomes, including any serious adverse event, any adverse events, and adverse effects related to the HPV vaccine. We extracted study characteristics (e.g. author, year, country, sample size and funding) participant characteristics (e.g. clinical condition, age, sex and immunosuppressive medication at baseline), intervention (e.g. vaccine type and number of doses) and the control intervention characteristics.

We assessed NRSI using the ‘Risk of Bias in Non-randomised Studies of Interventions’ tool (ROBINS-I).^30^ We did not proceed with the assessment when the risk of confounding was substantial.

Two reviewers (PK, LG) assessed the risk of bias of each individual study on outcome level and resolved any disagreements by consensus, moderated by a third reviewer (WS), if necessary. We assessed only NRSI, as we did not identify RCTs. We did not assess risk of bias of single-arm studies.

We summarised the certainty of evidence (CoE) using the Grading of Recommendations Assessment, Development and Evaluation (GRADE) approach.^31^^,^^32^ The summary of findings tables focused on prioritised, both desirable and undesirable health outcomes that are considered as critical and/or important for decision-making. Prioritised outcomes included precancer or cancer of the cervix, vulva, vagina, penis or anus and oropharyngeal cancer, HPV infection, immunogenicity parameters for HPV types 16 and 18, and any serious adverse events. We reported and interpreted results across all certainty levels, including outcomes rated as low or very low certainty, to ensure that data for decision-making is as informative as possible.

### Data analysis

We performed the meta-analyses using R version 4.5.2 and the meta package.^33^^,^^34^ We applied random-effect meta-analyses and planned to use the Hartung-Knapp adjustment when analyses for immunocompromised groups consistently reported three or more studies.^35^^,^^36^ Antibody titres are presented as GMR with its 95% CI and dichotomous outcomes as risk ratio (RR) with its 95% CI. We pooled data by applying the inverse variance method. Due to clinical heterogeneity across studies, we conducted meta-analyses and CoE assessments separately for each immunocompromised group (i.e. underlying disease). Residual heterogeneity in pooled analyses was assessed by visually inspecting differences in study estimates, their 95% CIs, complemented by the evaluation of I^2^ and prediction intervals in case of at least four studies. To ensure a comprehensive overview of the data, we included all studies in the meta-analyses regardless of bias risk. We planned subgroup analyses according to population characteristics (age, sex, and type of immunosuppressive therapy), intervention characteristics (type of HPV vaccine, number of doses, and ascertainment of vaccination), as well as study setting and study design. Sensitivity analyses included the exclusion of studies at critical risk of bias and the use of a fixed-effect model.

### Ethics

This study is a systematic review of previously published studies and does not involve the collection of primary data from human participants. Therefore, ethical approval was not required.

### Role of the funding source

This study was funded by the EU4Health Programme - European Health and Digital Executive Agency, European Commission. Service Contract HaDEA/OP/2021/0011. The funder had no role in the design, data collection, data analysis, data interpretation, or writing of the report.

## Results

**Fig. 1:**
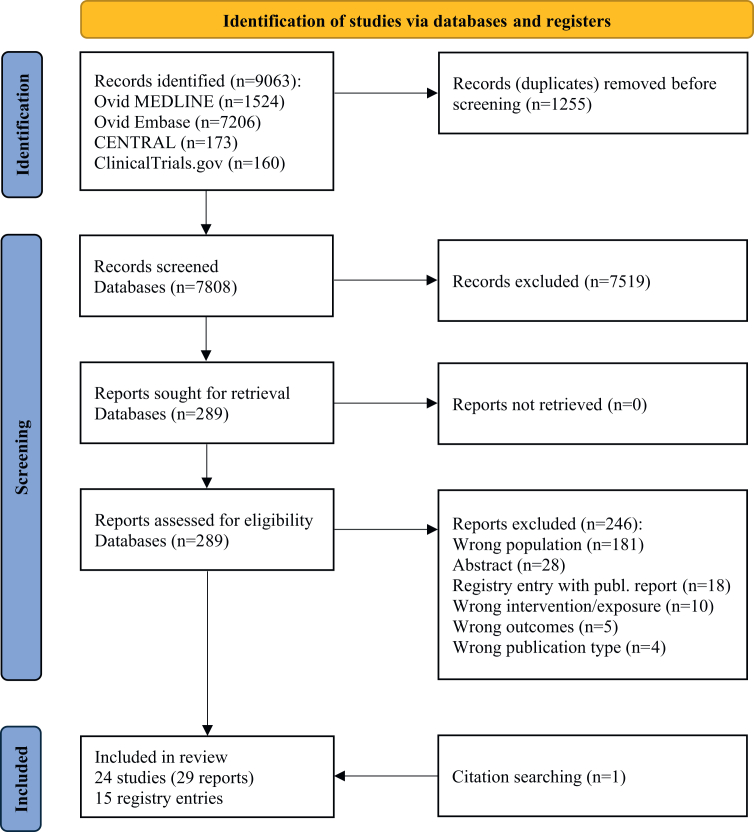
**PRISMA 2020 flow diagram.** n: number.

The literature search resulted in 9063 records. After deduplication, we screened 7808 titles and abstracts, and 290 full texts. Finally, we included 29 reports of 24 NRSI and additionally 15 registry entries (Table 1; Supplement Table S6). We did not find any RCT evidence on the efficacy, immunogenicity, or safety of HPV vaccination in immunocompromised individuals. The flow diagram and reasons for exclusion are provided in Fig. 1 and Supplement Table S7.

**Table 1:** Key study characteristics.

Study	Country	Study type	Funding	Clinical condition, intervention group	Clinical condition, control group	N intervention group	N control group	Intervention
Vaccinated immunocompromised group compared to unvaccinated immunocompromised control group with the same disease or condition (comparison 1)								
Grönlund 2016	Sweden	NRSI (registry based)	Public, non-profit	Autoimmune disease	Autoimmune disease	11,256	59,009	Quadrivalent HPV vaccine
Silverberg 2020	USA	NRSI (case–control)	Public, non-profit	Ever prior solid organ transplant, immunosuppressive therapy, HIV-infected^a^	Ever prior solid organ transplant, immunosuppressive therapy, HIV-infected^a^	4357 (cases)^b^	21,773 (controls)^b^	Quadrivalent HPV vaccine
Vaccinated immunocompromised group compared to other vaccinated immunocompromised control group with a different disease or condition that affects the immune system (comparison 2)								
Nailescu 2020	USA	NRSI (prospective)	Industry	Dialysis, transplant recipients (kidney, liver)	Chronic kidney disease (CKD)	47	18	Quadrivalent HPV vaccine
Nelson 2016	USA	NRSI (prospective)	Industry	Dialysis, transplant recipients (kidney)	CKD	38	29	Quadrivalent HPV vaccine
Vaccinated immunocompromised group compared to vaccinated healthy control group (comparison 3)								
Alter 2014	USA	NRSI (prospective/retrospective, historic control)	Public, non-profit	Fanconi anaemia (FA)^c^	Healthy participants	38	107	Quadrivalent HPV vaccine
Dhar 2017	USA	NRSI (prospective, historic control)	Industry	Systemic lupus erythematosus (SLE)	Healthy participants	37	NR	Quadrivalent HPV vaccine
Esposito 2014	Italy	NRSI (prospective)	Public, non-profit	Juvenile idiopathic arthritis (JIA)	Healthy participants	21	21	Bivalent HPV vaccine
Grein 2020a	Brazil	NRSI (prospective)	Public, non-profit	Childhood SLE	Healthy participants	234	41	Quadrivalent HPV vaccine
Grein 2020b	Brazil	NRSI (prospective)	Mixed	Juvenile dermatomyositis (JDM)	Healthy participants	47	41	Quadrivalent HPV vaccine
Gomez-Lobo 2014	USA	NRSI (prospective, historic control)	Industry	Transplant recipients (kidney, liver)	Healthy participants	20	5	Quadrivalent HPV vaccine
Heijstek 2014	Nether-lands	NRSI (prospective)	Mixed	JIA	Healthy participants	68	55	Bivalent HPV vaccine
Jacobson 2013	USA	NRSI (prospective, historic control)	Mixed	Inflammatory bowel disease (IBD)	Healthy participants	52	NR	Quadrivalent HPV vaccine
Kitano 2023	Canada	NRSI (prospective)	Industry	Transplant recipients (kidney, liver)	Healthy participants	17	19	Quadrivalent HPV vaccine
Landier 2022	USA	NRSI (prospective, historic control)	Mixed	Survivors of cancer^d^	Healthy participants	453	26,486	Intervention group: Quadrivalent HPV vaccine (58.3%) Nonavalent HPV vaccine (41.7%) Control group: Quadrivalent HPV vaccine and nonavalent HPV vaccine (proportions vary)
Miyaji 2024	Brazil	NRSI (prospective)	Public, non-profit	Transplant recipients (kidney, kidney + pancreas, liver, lung, heart)	Healthy participants	125	132	Quadrivalent HPV vaccine
Mok 2013	China	NRSI (prospective)	Industry	SLE	Healthy participants	50	50	Quadrivalent HPV vaccine
Nelson 2016	USA	NRSI (prospective, historic control)	Industry	Chronic kidney disease (CKD), Dialysis, transplant recipients (kidney)	Healthy participants	67	917–3329	Quadrivalent HPV vaccine
Sauter 2021	USA	NRSI (cross-sectional study)	Public, non-profit	FA	Healthy participants	212	111	Quadrivalent HPV vaccine (primarily)
Stratton 2020	USA	NRSI (prospective)	Public, non-profit	Allogeneic haematopoietic stem cell transplant recipients (post-HSCT)	Healthy participants	44	20	Quadrivalent HPV vaccine

CKD: chronic kidney disease; DBA: Diamond Blackfan anaemia, DC: dyskeratosis congenital; DoI: Declaration of Interests; FA: Fanconi anaemia; HPV: human papillomavirus; IBD: inflammatory bowel disease; JDM: juvenile dermatomyositis; JIA: juvenile idiopathic arthritis; N: number of participants; NR: not reported; NRSI: non-randomised studies of interventions; post-HSCT: allogeneic haematopoietic stem cell transplant; SD: standard deviations, SDS: Shwachman Diamond syndrome; SLE: systemic lupus erythematosus; TAR: thrombocytopaenia-absent radius.

a The study does not exclude HIV participants. However, cases with HIV were rare (intervention: 4 participants, control: 5 participants).

b This review incorporates only a subsample of this study.

c Including also DBA, DC, SDS and TAR.

d Including: leukaemia, lymphoma, solid tumour participants.

Overall, we identified two studies comparing vaccinated immunocompromised groups with unvaccinated immunocompromised control groups with the same disease or condition (comparison 1).^37^^,^^38^ Two studies provided indirect evidence in comparing vaccinated immunocompromised groups to vaccinated control groups with a different disease or condition that affects the immune system (comparison 2)^39^^,^^40^ and 15 studies with healthy participant groups that are not immunocompromised (comparison 3).^40^, ^41^, ^42^, ^43^, ^44^, ^45^, ^46^, ^47^, ^48^, ^49^, ^50^, ^51^, ^52^, ^53^, ^54^ One study contributed results for both comparison 2 and 3.^40^ Additionally, we identified six single-arm studies reporting safety and adverse outcomes.^55^, ^56^, ^57^, ^58^, ^59^, ^60^, ^61^ Across all comparisons, 15 studies provided data for analysis.^38^, ^39^, ^40^, ^41^, ^42^, ^43^^,^^45^, ^46^, ^47^, ^48^, ^49^, ^50^, ^51^, ^52^^,^^54^

Across comparisons, studies included in this review primarily reported on cervical precancers, immunogenicity at month 7 (i.e. after the intended completion of the vaccination series), and safety (Table 2, Table 3, Table 4, Table 5 and Table 6). None of the studies reported outcomes on precancers or cancers of the vulva, vagina, penis, or anus, oropharyngeal cancer, HPV infection, HPV-related mortality, or anogenital warts. We judged nine studies to have an overall serious risk of bias, mainly due to confounding (i.e. studies considered not all baseline confounders that are possibly relevant), selection of the participants into the study (i.e. some participants were retrospectively included into the study) and selection of the reported results (e.g. potential deviations from the study plan).^38^^,^^39^^,^^43^^,^^45^^,^^47^^,^^49^^,^^51^^,^^52^ Additionally, seven studies had a critical risk of bias, due to very problematic confounding (e.g. uncontrolled differences in participant characteristics at baseline).^40^, ^41^, ^42^^,^^46^^,^^48^^,^^50^^,^^54^ For one study, the risk-of-bias assessment varied by outcome, with a critical risk of bias for serious adverse events and a serious risk of bias for immunogenicity outcomes.^40^ The detailed ROBINS-I judgements for each outcome are presented in Supplement Table S8.

**Table 2:** Summary of findings, vaccinated immunocompromised group compared to unvaccinated immunocompromised control group with the same disease or condition (comparison 1) Outcomes: CIN 2+ and CIN 3+.

Studies (participants)	Results	Certainty	Interpretation
Participants: Mixed population^a^			
CIN 2+			
1 NRSI (case–control study: 506 cases, 2672 controls)^38^	The study reports an adjusted rate ratio of 0.96, 95 % CI (0.68–1.37)^b^	Very low^c^^,^^d^	The evidence from NRSI is of very low certainty about the effect of HPV vaccination on CIN 2+.
CIN 3+			
1 NRSI (case–control study: 215 cases, 1142 controls)^38^	The study reports an adjusted rate ratio of 0.96, 95% CI (0.54–1.70)^b^	Very low^c^^,^^d^	The evidence from NRSI is of very low certainty about the effect of HPV vaccination on CIN 3+.

CI: confidence interval; CIN 2+: cervical intraepithelial neoplasia grade 2+; CIN 3+: cervical intraepithelial neoplasia grade 3+; HPV: human papillomavirus; NRSI: non-randomised studies of interventions.

a Including: ever prior solid organ transplant, immunosuppressive therapy, HIV-infected.

b As reported in the study and adjusted for immunosuppression history, vaccination, immunosuppression, smoking, hormone therapy or oral contraceptives, race and ethnicity, recent sexually transmitted infections, parity, and prior number of outpatient visits.

c Risk of bias downgraded by one level: mainly due to serious concerns regarding confounding and selection of participants into the study.

d Imprecision downgraded by two levels: due to the considerably wide 95% CI of the rate ratio.

**Table 3:** Summary of findings, vaccinated immunocompromised group vs. other vaccinated immunocompromised control group with a different disease or condition that affects the immune system (comparison 2) Outcome: Seropositivity, 7 months.

HPV type	Studies (participants)	Relative effect (95% CI)	Absolute effects (95% CI)			Certainty	Interpretation
			Other vaccinated immunocompromised group (chronic kidney disease)	Vaccinated immunocompromised group (dialysis or transplant)	Risk difference		
Participants: Dialysis compared to chronic kidney disease (CKD)							
HPV 16	2 NRSI (27 participants in intervention group, 27 in control group)^39^^,^^40^	**RR 0.96** (0.87–1.05)	1000 per 1000	**960 per 1000** (870–1000)	**40 fewer per 1000** (from 130 fewer to 50 more)	Low^a^^,^^b^	The evidence is of low certainty, but suggests that there may be little to no difference in seropositivity rates for HPV 16 between dialysis and CKD participants.
HPV 18	2 NRSI (27 participants in intervention group, 27 in control group)^39^^,^^40^	**RR 0.98** (0.81–1.17)	963 per 1000	**944 per 1000** (780–1000)	**19 fewer per 1000** (from 183 fewer to 164 more)	Low^a^^,^^b^	The evidence is of low certainty, but suggests that there may be little to no difference in seropositivity rates for HPV 18 between dialysis and CKD participants.
Participants: Transplant compared to chronic kidney disease (CKD)							
HPV 16	2 NRSI (51 participants in intervention group, 27 in control group)^39^^,^^40^	**RR 0.94** (0.86–1.03)	1000 per 1000	**940 per 1000** (860–1000)	**60 fewer per 1000** (from 140 fewer to 30 more)	Low^a^^,^^b^	The evidence is of low certainty, but suggests that there may be little to no difference in seropositivity rates for HPV 16 between transplant and CKD participants.
HPV 18	2 NRSI (51 participants in intervention group, 27 in control group)^39^^,^^40^	**RR 0.77** (0.63–0.94)	963 per 1000	**741 per 1000** (607–905)	**221 fewer per 1000** (from 356 fewer to 58 fewer)	Very low^a^^,^^c^	The evidence is of very low certainty about the effect of HPV vaccination on seropositivity rates for HPV 18. There may be a reduction in seropositivity rates in transplant compared to CKD participants.

CI: confidence interval; CKD: chronic kidney disease; HPV: human papillomavirus; NRSI: non-randomised studies of interventions; RR: risk ratio.

a Risk of bias downgraded by one level: serious concerns regarding confounding, selection of participants into the study, missing data and measurement of outcomes.

b Imprecision downgraded by one level: due to absolute differences that indicate fewer or more events and imprecision due to a small sample size.

c Imprecision downgraded by two levels: due to absolute differences that indicate considerably fewer or more events and imprecision due to a small sample size.

**Table 4:** Summary of findings, vaccinated immunocompromised group compared to vaccinated healthy control group (comparison 3) Outcome: Seropositivity, 7 months.

HPV type	Studies (participants)	Relative effect (95% CI)	Absolute effects (95% CI)			Certainty	Interpretation
			Vaccinated healthy control group	Vaccinated immunocompromised group	Risk difference		
Participants: Cancer survivors^a^							
HPV 16	1 NRSI (358 participants in intervention group, 14,923 in control group)^50^	**RR 1.002**^b^ (1.000–1.003)	998 per 1000	**1000 per 1000** (998–1.000)	**2 more per 1000** (from 0 fewer to 3 more)	Low^c^	The evidence is of low certainty, but suggests that there may be no difference in seropositivity rates for HPV 16 between cancer survivors and healthy participants.
HPV 18	1 NRSI (369 participants in intervention group, 15,834 in control group)^50^	**RR 1.001**^b^ (1.001–1.002)	996 per 1000	**997 per 1000** (997–998)	**1 more per 1000** (from 1 more to 2 more)	Low^c^	The evidence is of low certainty, but suggests that there may be no difference in seropositivity rates for HPV 18 between cancer survivors and healthy participants.
Participants: Inflammatory bowel disease (IBD)							
HPV 16	1 NRSI (33 participants in intervention group, 4164 in control group)^48^	**RR 1.002**^b^ (1.000–1.003)	998 per 1000	**1000 per 1000** (998–1000)	**2 more per 1000** (from 0 fewer to 3 more)	Low^c^	The evidence is of low certainty, but suggests that there may be no difference in seropositivity rates for HPV 16 between IBD and healthy participants.
HPV 18	1 NRSI (33 participants in intervention group, in control group 4488)^48^	**RR 0.95** (0.87–1.03)	995 per 1000	**940 per 1000** (864–1000)	**55 fewer per 1000** (from 131 fewer to 29 more)	Very low^c^^,^^d^	The evidence is of low certainty, but suggests that there may be little to no difference in seropositivity rates for HPV 18 between IBD and healthy participants.
Participants: Juvenile dermatomyositis (JDM)							
HPV 16	1 NRSI (31 participants in intervention group, 15 in control group)^46^	**RR 1.00** (0.90–1.11)	1000 per 1000	**1000 per 1000** (904–1000)	**0 fewer per 1000** (from 96 fewer to 106 more)	Very low^c^^,^^d^	The evidence is of very low certainty about the effect of HPV vaccination on seropositivity rates for HPV 16. There may be little to no difference in seropositivity between JDM and healthy participants.
HPV 18	1 NRSI (31 participants in intervention group, 15 in control group)^46^	**RR 0.97** (0.91–1.03)	1000 per 1000	**968 per 1000** (909–1000)	**32 fewer per 1000** (from 91 fewer to 31 more)	Very low^c^^,^^d^	The evidence is of very low certainty about the effect of HPV vaccination on seropositivity rates for HPV 18. There may be little to no difference in seropositivity between JDM and healthy participants.
Participants: Juvenile idiopathic arthritis (JIA)							
HPV 16	2 NRSI (62 participants in intervention group, 62 in control group)^43^^,^^47^	**RR 1.00** (0.96–1.04)	1000 per 1000	**1000 per 1000** (959–1000)	**0 fewer per 1000** (from 41 fewer to 43 more)	Low^e^^,^^f^	The evidence is of low certainty, but suggests that there may be no difference in seropositivity rates for HPV 16 between JIA and healthy participants
HPV 18	2 NRSI (62 participants in intervention group, 62 in control group)^43^^,^^47^	**RR 1.00** (0.96–1.04)	1000 per 1000	**1000 per 1000** (959–1000)	**0 fewer per 1000** (from 41 fewer to 43 more)	Low^e^^,^^f^	The evidence is of low certainty, but suggests that there may be no difference in seropositivity rates for HPV 18 between JIA and healthy participants.
Participants: Allogeneic haematopoietic stem cell transplant (post-HSCT)							
HPV 16	1 NRSI (44 participants in intervention group, 20 in control group)^52^	**RR 0.97** (0.90–1.04)	1000 per 1000	**968 per 1000** (899–1000)	**32 fewer per 1000** (from 101 fewer to 42 more)	Low^e^^,^^d^	The evidence is of low certainty, but suggests that there may be little to no difference in seropositivity rates for HPV 16 between post-HSCT and healthy participants.
HPV 18	1 NRSI (44 participants in intervention group, 20 in control group)^52^	**RR 0.95** (0.86–1.04)	1000 per 1000	**948 per 1000** (862–1000)	**52 fewer per 1000** (from 138 fewer to 43 more)	Low^e^^,^^d^	The evidence is of low certainty, but suggests that there may be little to no difference in seropositivity rates for HPV 18 between post-HSCT and healthy participants.
Participants: Systemic lupus erythematosus (SLE)							
HPV 16	3 NRSI (181 participants in intervention group, 717 in control group)^41^^,^^45^^,^^51^	**RR 0.99** (0.95–1.03)	982 per 1000	**970 per 1000** (928–1000)	**12 fewer per 1000** (from 54 fewer to 32 more)	Very low^c^^,^^d^	The evidence is of very low certainty about the effect of HPV vaccination on seropositivity rates for HPV 16. There may be little to no difference in seropositivity rates between SLE and healthy participants.
HPV 18	3 NRSI (188 participants in intervention group, 778 in control group)^41^^,^^45^^,^^51^	**RR 0.94** (0.83–1.06)	963 per 1000	**908 per 1000** (804–1000)	**55 fewer per 1000** (from 159 fewer to 63 more)	Very low^c^^,^^d^	The evidence is of very low certainty about the effect of HPV vaccination on seropositivity rates for HPV 18. There may be little to no difference in seropositivity rates between SLE and healthy participants.
Participants: Transplant recipients (kidney and liver)							
HPV 16	1 NRSI (10 participants in intervention group, 3 in control group)^49^	**RR 0.81** (0.60–1.09)	1000 per 1000	**810 per 1000** (604–1000)	**190 fewer per 1000** (from 396 fewer to 86 more)	Very low^e^^,^^g^	The evidence is of very low certainty about the effect of HPV vaccination on seropositivity rates for HPV 16. There may be reduced or slightly increased seropositivity rates in transplant compared to healthy participants.
HPV 18	1 NRSI (10 participants in intervention group, 3 in control group)^49^	**RR 0.90** (0.74–1.10)	1000 per 1000	**905 per 1000** (744–1000)	**95 fewer per 1000** (from 256 fewer to 101 more)	Very low^e^^,^^g^	The evidence is of very low certainty about the effect of HPV vaccination on seropositivity rates for HPV 18. There may be reduced or slightly increased seropositivity rates in transplant compared to healthy participants.
Participants: Transplant recipients (kidney, kidney + pancreas, liver, lung, heart)							
HPV 16	1 NRSI (77 participants in intervention group, 87 in control group)^42^	**RR 0.69** (0.59–0.80)	1000 per 1000	**690 per 1000** (590–600)	**310 fewer per 1000** (from 410 fewer to 200 fewer)	Very low^c^^,^^f^	The evidence is of very low certainty about the effect of HPV vaccination on seropositivity rates for HPV 16. There may be a reduction in seropositivity rates in transplant compared to healthy participants.
HPV 18	1 NRSI (89 participants in intervention group, 95 in control group)^42^	**RR 0.58** (0.48–0.69)	1000 per 1000	**580 per 1000** (480–690)	**420 fewer per 1000** (from 520 fewer to 310 fewer)	Very low^c^^,^^f^	The evidence is of very low certainty about the effect of HPV vaccination on seropositivity rates for HPV 18. There may be a reduction in seropositivity rates in transplant compared to healthy participants.

CI: confidence interval; CKD: chronic kidney disease; HPV: human papillomavirus; IBD: inflammatory bowel disease; JDM: juvenile dermatomyositis; JIA: juvenile idiopathic arthritis; NRSI: non-randomised studies of interventions; post-HSCT: allogeneic haematopoietic stem cell transplant; RR: risk ratio; SLE: systemic lupus erythematosus.

a Including: leukaemia, lymphoma, solid tumour participants.

b Three decimal places are reported to provide numerical detail when the point estimate and its 95% CI are near the null effect. Data in the corresponding meta-analyses are presented with two decimal places.

c Risk of bias downgraded by two levels: due to very serious concerns regarding confounding.

d Imprecision downgraded by one level: due to absolute differences that indicate fewer or more events and imprecision due to a small sample size.

e Risk of bias downgraded by one level: mainly due to serious concerns regarding confounding, selection of participants into the study, missing outcome data or measurement of the outcome.

f Imprecision downgraded by one level: due to a small sample size.

g Imprecision downgraded by two levels: due to absolute differences that indicate considerably fewer or more events and imprecision due to a small sample size.

**Table 5:** Summary of findings, Serious adverse events: comparison 2 to comparison 3 Outcome: serious adverse event.

Outcome No of participants (studies)	Result	Certainty	Interpretation
Safety outcomes: Serious adverse events			
Participants: All participant populations			
10 NRSI^c^ (any time point, ≈600 participants in intervention group, ≈400 control group).	Overall, most studies do not report any serious adverse events for the immunocompromised individuals or healthy participants at all, or only a small number of serious adverse events that were judged to be unrelated to the HPV vaccine.	Very low^a^^,^^b^	The evidence is of very low certainty about the effect of HPV vaccination on serious adverse events.

HPV: human papillomavirus; NRSI: non-randomised studies of interventions.

a Risk of bias downgraded by two levels: due to very serious concerns regarding confounding.

b Inconsistency downgraded by one level: due to slightly varying effects between the included studies.

c Studies reporting data for both the intervention and control groups. Number of participants incompletely reported in studies.

**Table 6:** Summary of immunogenicity outcomes.

Outcome, number of studies	Events/total (intervention) or total	Events/total (control)	Estimate, 95%-CI	Heterogeneity (I^2^)	Overall RoB
Vaccinated immunocompromised group compared to other vaccinated immunocompromised control group with a different disease or condition that affects the immune system (comparison 2)					
Participants: Dialysis compared to chronic kidney disease (CKD)					
Seropositivity HPV 16, 7 months, 2 NRSI	26/27	27/27	**RR 0.96** (0.87–1.05)	0%	Serious
Seropositivity HPV 18, 7 months, 2 NRSI	24/27	26/27	**RR 0.98** (0.81–1.17)	0%	Serious
Participants: Transplant compared to CKD					
HPV 16, 7 months, 2 NRSI	48/51	27/27	**RR 0.94** (0.86–1.03)	0%	Serious
HPV 18, 7 months, 2 NRSI	34/51	26/27	**RR 0.77** (0.63–0.94)	7.3%	Serious
Vaccinated immunocompromised group compared to vaccinated healthy control group (comparison 3)					
Participants: Cancer survivors^a^					
Seropositivity HPV 16, 7 months, 1 NRSI	358/358	14,897/14,923	**RR 1.002** (1.001–1.003)^b^	38.1%	Critical
Seropositivity HPV 18, 7 months, 1 NRSI	367/369	15,766/15,834	**RR 1.001** (1.001–1.002)^b^	0%	Critical
GMR HPV 16, 7 months, 1 NRSI	358 (total)	14,923 (total)	**GMR 2.59** (2.05–3.26)	47.9%	Critical
GMR HPV 18, 7 months, 1 NRSI	369 (total)	15,834 (total)	**GMR 2.52** (1.94–3.27)	54.5%	Critical
Participants: Fanconi anaemia (FA)					
Seropositivity HPV 16, 12 months and more, 1 NRSI	53/60	19/21	**RR 0.98** (0.83–1.15)	NA	Critical
Seropositivity HPV 18, 12 months and more, 1 NRSI	44/60	19/21	**RR 0.81** (0.66–1.00)	NA	Critical
GMR HPV 16, 12 months and more, 1 NRSI	37 (total)	21 (total)	**GMR 0.59** (0.13–2.64)	0%	Critical
GMR HPV 18, 12 months and more, 1 NRSI	37 (total)	21 (total)	**GMR 0.56** (0.12–2.58)	22.2%	Critical
Participants: Inflammatory bowel disease (IBD)					
Seropositivity HPV 16, 7 months, 1 NRSI	33/33	4157/4164	**RR 1.002** (1.000–1.003)^b^	0%	Critical
Seropositivity HPV 18, 7 months, 1 NRSI	31/33	4465/4488	**RR 0.95** (0.87–1.03)	0%	Critical
GMR HPV 16, 7 months, 1 NRSI	33	4168	**GMR 1.06** (0.60–1.88)	27.3%	Critical
GMR HPV 18, 7 months, 1 NRSI	33	4493	**GMR 1.12** (0.62–2.02)	0%	Critical
Participants: Juvenile dermatomyositis (JDM)					
Seropositivity HPV 16, 7 months, 1 NRSI	31/31	15/15	**RR 1.00** (0.90–1.11)	NA	Critical
Seropositivity HPV 18, 7 months, 1 NRSI	30/31	15/15	**RR 0.97** (0.91–1.03)	NA	Critical
Participants: Juvenile idiopathic arthritis (JIA)					
Seropositivity HPV 16, 7 months, 2 NRSI	62/62	62/62	**RR 1.00** (0.96–1.04)	0%	Serious
Seropositivity HPV 16, 12 months and more, 1 NRSI	42/43	44/44	**RR 0.98** (0.93–1.02)	NA	Serious
Seropositivity HPV 18, 7 months, 2 NRSI	62/62	62/62	**RR 1.00** (0.96–1.04)	0%	Serious
Seropositivity HPV 18, 12 months and more, 1 NRSI	42/43	44/44	**RR 0.98** (0.93–1.02)	NA	Serious
GMR HPV 16, 7 months, 1 NRSI	41 (total)	41 (total)	**GMR 0.40** (0.20–0.82)	NA	Serious
GMR HPV 16, 12 months and more, 1 NRSI	43 (total)	44 (total)	**GMR 0.44** (0.22–0.87)	NA	Serious
GMR HPV 18, 7 months, 1 NRSI	41 (total)	41 (total)	**GMR 0.52** (0.27–1.01)	NA	Serious
GMR HPV 18, 12 months and more, 1 NRSI	43 (total)	44 (total)	**GMR 0.63** (0.31–1.25)	NA	Serious
Participants: Allogeneic haematopoietic stem cell transplant (post-HSCT)					
Seropositivity HPV 16, 7 months, 1 NRSI	43/44	20/20	**RR 0.97** (0.90–1.04)	0%	Serious
Seropositivity HPV 18, 7 months, 1 NRSI	42/44	20/20	**RR 0.95** (0.86–1.04)	0%	Serious
GMR HPV 16, 7 months, 1 NRSI	44 (total)	20 (total)	**GMR 0.88** (0.40–1.97)	0%	Serious
GMR HPV 16, 12 months and more, 1 NRSI	44 (total)	20 (total)	**GMR 0.86** (0.40–1.85)	0%	Serious
GMR HPV 18, 7 months, 1 NRSI	44 (total)	20 (total)	**GMR 0.74** (0.37–1.48)	0%	Serious
GMR HPV 18, 12 months and more, 1 NRSI	44 (total)	20 (total)	**GMR 0.88** (0.46–1.70)	0%	Serious
Participants: Systemic lupus erythematosus (SLE)					
Seropositivity HPV 16, 7 months, 3 NRSI	174/181	704/717	**RR 0.99** (0.95–1.03)	81.2%	Critical
Seropositivity HPV 16, 12 months and more, 1 NRSI	37/39	43/44	**RR 0.97** (0.89–1.06)	NA	Serious
Seropositivity HPV 18, 7 months, 3 NRSI	168/188	749/778	**RR 0.94** (0.83–1.06)	91.9%	Critical
Seropositivity HPV 18, 12 months and more, 1 NRSI	29/38	32/40	**RR 0.95** (0.75–1.21)	NA	Serious
GMR HPV 16, 7 months, 1 NRSI	19 (total)	657 (total)	**GMR 1.43** (1.02–2.02)	NA	Critical
GMR HPV 18, 7 months, 1 NRSI	27 (total)	722 (total)	**GMR 1.75** (1.23–2.48)	NA	Critical
Participants: Transplant recipients (kidney and liver)					
Seropositivity HPV 16, 7 months, 1 NRSI	8/10	3/3	**RR 0.81** (0.60–1.09)	NA	Serious
Seropositivity HPV 18, 7 months, 1 NRSI	9/10	3/3	**RR 0.90** (0.74–1.10)	NA	Serious
Participants: Transplant recipients (kidney)					
Seropositivity HPV 16, 12 months and more, 1 NRSI	4/6	13/13	**RR 0.69** (0.41–1.16)	NA	Serious
Seropositivity HPV 18, 12 months and more, 1 NRSI	4/6	13/13	**RR 0.69** (0.41–1.16)	NA	Serious
Participants: Transplant recipients (liver)					
Seropositivity HPV 16, 12 months and more, 1 NRSI	6/6	13/13	**RR 1.00** (0.78–1.28)	NA	Serious
Seropositivity HPV 18, 12 months and more, 1 NRSI	5/6	13/13	**RR 0.85** (0.61–1.17)	NA	Serious
Participants: Transplant recipients (kidney, kidney + pancreas, liver, lung, heart)					
Seropositivity HPV 16, 7 months, 1 NRSI	53/77	87/87	**RR 0.69** (0.59–0.80)	NA	Critical
Seropositivity HPV 18, 7 months, 1 NRSI	51/89	95/95	**RR 0.58** (0.48–0.69)	NA	Critical
GMR HPV 16, 7 months, 1 NRSI	81 (total)	88 (total)	**GMR 0.13** (0.07–0.25)	NA	Critical
GMR HPV 18, 7 months, 1 NRSI	67 (total)	96 (total)	**GMR 0.13** (0.07–0.26)	NA	Critical

CI: confidence interval; CKD: Chronic kidney disease; FA: Fanconi anaemia; GMR: geometric mean ratio; HPV: human papillomavirus; IBD: inflammatory bowel disease; JDM: juvenile dermatomyositis; JIA: juvenile idiopathic arthritis; NA: not applicable; NR: not reported; NRSI: non-randomised studies of interventions; post-HSCT: allogeneic haematopoietic stem cell transplant; RoB: Risk of Bias; RR: risk ratio; SLE: systemic lupus erythematosus.

a Including: leukaemia, lymphoma, solid tumour participants.

b Three decimal places are reported to provide numerical detail when the point estimate and its 95% CI are near the null effect. Data in the corresponding meta-analyses are presented with two decimal places.

We included two NRSI comparing vaccinated immunocompromised groups to unvaccinated immunocompromised control groups with the same disease or condition (comparison 1): one registry-based cohort study^37^ and one case–control study.^38^ Participants of the included studies comprised various immunocompromised groups, including solid organ transplant recipients, individuals with autoimmune diseases, or individuals undergoing immunosuppressive therapy. Both studies reported on participants that received the quadrivalent HPV vaccine.^37^^,^^38^ One study reported that participants received a minimum of one dose,^38^ while Grönlund et al. reported that approximately 60% received three doses of the HPV vaccine.^37^ Table 1 and Supplement Table S9 provide further details on study characteristics.

The study by Silverberg et al. reported on vaccine effectiveness and showed no reduction in CIN 2+ and CIN 3+ associated with quadrivalent HPV catch-up vaccination at the age of 18–29 years (adjusted rate ratio 0.96, 95% CI 0.68–1.37 for CIN 2+; 0.96, 95% CI 0.54–1.70 for CIN 3+; Table 2). We assessed the CoE to be very low (due to serious concerns in risk of bias and considerable imprecision).^38^ The studies did not provide data for the analysis on other prespecified effectiveness and immunogenicity outcomes.

None of the studies identified for comparison 1 assessed vaccine safety. However, the study by Grönlund et al. reported the incidence of new-onset autoimmune disease in participants with pre-existing autoimmune diseases. There was no increase in the incidence of new-onset autoimmune disease associated with the quadrivalent HPV vaccination (adjusted incidence rate ratio 0.77, 95% CI 0.65–0.93); in fact, a slightly reduced risk was observed.^37^

We included two NRSI comparing vaccinated immunocompromised groups to other vaccinated immunocompromised control groups with a different disease or condition that affects the immune system (comparison 2).^39^^,^^40^ Participants of included studies comprised immunocompromised groups undergoing dialysis, solid organ transplant recipients, and chronic kidney disease (CKD). All participants received the quadrivalent HPV vaccine. Most included participants received three doses of the HPV vaccine (range 85.1%–90.3%).^39^^,^^40^ Further details on study characteristics are reported in Table 1 and Supplement Table S9.

**Fig. 2:**
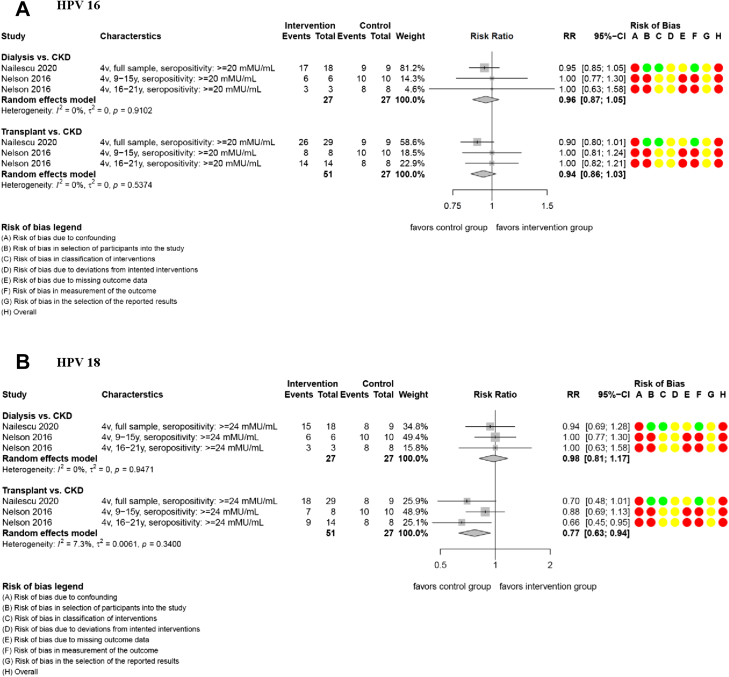
**Seropositivity of HPV 16 (A) and 18 (B) at 7 months (comparison 2).** Meta-analyses are presented for each immunocompromised group, alongside the risk of bias assessment. CI: confidence interval; CKD: chronic kidney disease; HPV: human papillomavirus; mMU/mL: milli-Merck Units per millilitre; y: years.

The included studies did not provide data on prespecified effectiveness outcomes. Most immunogenicity data showed that HPV vaccination is associated with high to very high seropositivity rates for HPV types 16 and 18 (66.7%–100%) in individuals undergoing dialysis, solid organ transplant recipients and CKD participants.^39^^,^^40^ Most analyses indicated that there may be little to no difference in seropositivity rates between different immunocompromised groups for HPV types 16 and 18 at 7 months (Tables 3 and 6; Fig. 2). In solid organ transplant recipients, there may be reduced seropositivity rates compared to CKD participants for HPV types 18 at 7 months (RR 0.77, 95% CI 0.63–0.94), 2 NRSI; Tables 3 and 6; Fig. 2).^39^^,^^40^ We assessed the CoE for analyses in comparator 2 to be low to very low (mainly due to serious concerns in risk of bias and imprecision; Table 3). The studies did not provide sufficient data for the analysis of GMRs, longer follow-up time points or other prespecified outcomes (Supplement Table S10 and S11).

Both included studies reported on vaccine safety. Nailescu et al. did not identify serious adverse events in either of the investigated groups (Table 5; Supplement Table S12).^39^ Both Nailescu et al. and Nelson et al. observed cases of acute organ rejections, but deemed these unrelated to HPV vaccination.^39^^,^^40^ Nelson et al. additionally reported local and systemic events, such as pain, bruising at injection site and headache after HPV vaccination among participants with CKD, dialysis and solid organ transplant recipients (Supplement Table S13).^40^

We included 15 NRSI comparing vaccinated immunocompromised groups to vaccinated healthy control groups (comparison 3).^40^, ^41^, ^42^, ^43^, ^44^, ^45^, ^46^, ^47^, ^48^, ^49^, ^50^, ^51^, ^52^, ^53^, ^54^ Participants of identified studies comprised various immunocompromised groups, including allogeneic haematopoietic stem cell transplant recipients (post-HSCT), CKD, dialysis, Fanconi anaemia (FA), inflammatory bowel disease (IBD), juvenile dermatomyositis (JDM), juvenile idiopathic arthritis (JIA), solid organ transplant recipients, survivors of cancer, and systemic lupus erythematosus (SLE). Most studies reported on participants that received the quadrivalent HPV vaccine (n = 12),^40^, ^41^, ^42^^,^^44^, ^45^, ^46^^,^^48^^,^^49^^,^^51^, ^52^, ^53^, ^54^ two studies on the bivalent HPV vaccine,^43^^,^^47^ and one study on different HPV vaccines (nonavalent and quadrivalent HPV vaccines).^50^ In the majority of studies, participants received three doses of the HPV vaccine (range 70.3%–100%),^40^, ^41^, ^42^, ^43^, ^45^, ^46^, ^47^, ^48^, ^49^, ^50^, ^51^, ^52^, ^53^ while one study reported that less than 40% of the participants received three HPV vaccine doses.^44^ One study did not provide information on dosing.^54^ Seven studies did not report dosing information for the control group (i.e. healthy participants).^40^^,^^41^^,^^44^^,^^48^^,^^50^^,^^53^^,^^54^ Further details on study characteristics are reported in Table 1 and Supplement Table S9.

**Fig. 3:**
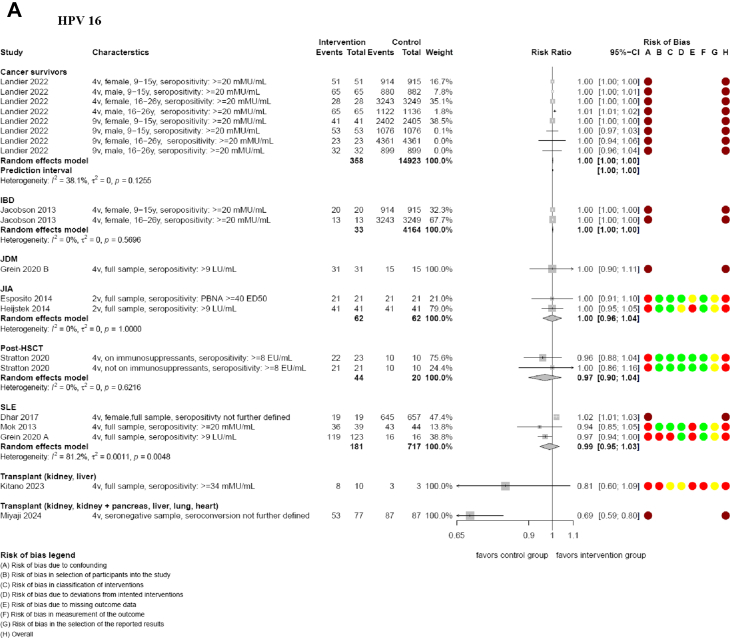

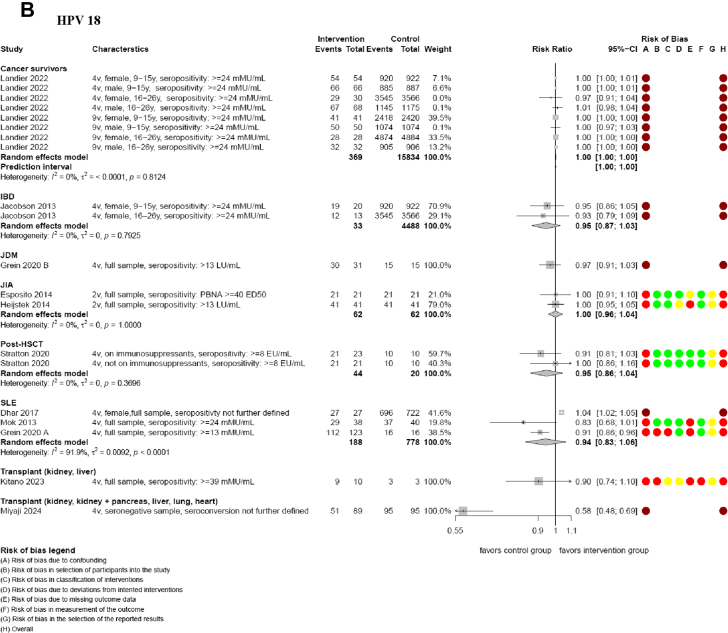
**Seropositivity of HPV 16 (A) and 18 (B) at 7 months (comparison 3).** Meta-analyses are presented for each immunocompromised group, alongside the risk of bias assessment. CI: confidence interval; EU/mL: ELISA Units per millilitre; ED50: effective dose producing a response in 50% of subjects; HPV: human papillomavirus; IBD: inflammatory bowel disease; JDM: Juvenile dermatomyositis; JIA: juvenile idiopathic arthritis; LU/mL: Luminex Units per millilitre; PBNA: pseudoviron-based neutralization assay; post-HSCT: allogeneic hematopoietic stem cell transplant; mMU/mL: milli-Merck Units per millilitre; SLE: systemic lupus erythematosus; y: years; 2v: bivalent; 4v: quadrivalent; 9v: nonavalent.

The included studies did not provide data on prespecified effectiveness outcomes. Most immunogenicity data analyses showed that HPV vaccination was associated with high to very high rates of seropositivity for HPV types 16 and 18 (range 57.3%–100%) in cancer survivors, FA, IBD, JDM, JIA, post-HSCT, SLE, solid organ transplant recipient, and healthy participants.^41^, ^42^, ^43^^,^^45^, ^46^, ^47^, ^48^, ^49^, ^50^, ^51^, ^52^^,^^54^ Most analyses indicated that there may be little to no difference in seropositivity rates in immunocompromised groups compared to healthy participants for HPV types 16 and 18 at 7 months (Tables 4 and 6, Fig. 3).^41^^,^^43^^,^^45^, ^46^, ^47^, ^48^, ^49^, ^50^, ^51^, ^52^ However, analyses in kidney, kidney and pancreas, liver, lung and heart transplant recipients suggested that seropositivity rates may be reduced compared to healthy participants (e.g. HPV type 16 at 7 months RR 0.69, 95% CI 0.59–0.80; 1 NRSI), although these findings were limited by a small sample size and a critical risk of bias.^42^ Evidence from other studies on liver and kidney transplant recipients, as well as participants with FA or SLE, showed wide confidence intervals, with effects that may favour healthy participants, show no difference, or favour immunocompromised individuals (e.g. kidney and liver transplant recipients for HPV type 16 at 7 months RR 0.81, 95% CI 0.60–1.09; 1 NRSI; SLE participants for HPV 18 at 7 months RR 0.94, 95% CI 0.84–1.07; 3 NRSI; Tables 4 and 6; Fig. 3).^41^^,^^45^^,^^49^^,^^51^ Results at 7 months were similar to follow-up timepoints at 12 months and more (Table 6; Supplement Table S14, Supplement Figure S1). We assessed the CoE for analyses in comparator 3 on seropositivity to be low to very low (mainly due to serious concerns in risk of bias and imprecision; Table 4; Supplement Table S14).

GMRs of antibody titres varied between immunocompromised groups and healthy participants, with analyses showing both potentially decreased or increased antibody titres compared to those of healthy participants.^42^ Results at 7 months were similar to follow-up time points at 12 months and more. We assessed the CoE for analyses in comparator 3 on GMRs to be low to very low (mainly due to serious concerns in risk of bias and imprecision; Supplement Table S15). Full details on GMRs of antibody titres are presented in Table 6, Supplement Table S16–S19, and Supplement Figure S2 and S3.

Most studies that reported on vaccine safety did not identify serious adverse events in the HPV vaccination groups (Supplement Table S12).^43^^,^^44^^,^^46^^,^^49^^,^^51^^,^^52^ Studies that reported serious adverse event generally deemed HPV vaccination unrelated to these events.^41^^,^^42^^,^^45^^,^^47^^,^^48^ We assessed the CoE for serious adverse events across comparison 2 and 3 to be very low (mainly due to serious concerns in risk of bias and inconsistency; Table 5). All studies reported on local (e.g. pain, induration, erythema or oedema) and systemic adverse events (e.g. headache, fatigue and nausea). Three studies reported that some local events (e.g. oedema, erythema and pain) were more frequent in healthy participants compared to the immunocompromised groups.^47^^,^^50^^,^^52^ Studies did not report any major differences in systemic adverse events compared to healthy participants. Some studies reported additional adverse events, such as rectal bleeding and diarrhoea, or disease related adverse events (Supplement Table S13).^41^^,^^42^^,^^44^^,^^48^^,^^50^^,^^51^

We included six single-arm studies of vaccinated immunocompromised groups.^55^, ^56^, ^57^, ^58^, ^59^, ^60^, ^61^ Participants of identified studies encompassed various immunocompromised groups, including CKD, SLE, solid organ transplant recipients and mixed groups with different underlying diseases. One study reported on participants that received the nonavalent HPV vaccine,^56^ while the remaining five studies received the quadrivalent HPV vaccine.^55^^,^^57^, ^58^, ^59^, ^60^, ^61^ In most studies the majority of participants received three doses of the HPV vaccine (range 74.1%–100%),^55^, ^56^, ^57^^,^^59^, ^60^, ^61^ while one study did not give the exact information on dosing.^58^ Further details on study characteristics are reported in Supplement Table S20.

Boey et al. reported that eight participants experienced at least one serious adverse event within 1–15 days after any vaccine dose, while 28 participants experienced an serious adverse event within 7 months of the first dose. Investigators deemed none of them related to the HPV vaccine.^56^ None of the remaining studies reported any serious adverse event.^55^^,^^57^, ^58^, ^59^, ^60^, ^61^ Three studies reported on local (e.g. pain and tenderness at injection side) and systemic adverse events (e.g. headache and fatigue; Supplement Table S13).^56^^,^^58^^,^^60^

Meaningful subgroup analyses were not feasible across comparisons, as we analysed all immunocompromised groups based on their underlying disease separately. We conducted sensitivity analyses on seropositivity for HPV types 16 and 18 at 7 months based on the risk of bias assessment (excluding studies with critical risk of bias) for comparison 3. The effects appeared comparable to the primary analyses on seropositivity for HPV types 16 and 18, with effect estimates and confidence intervals indicating no relevant differences (HPV 16) or a slightly favourable effect (HPV 18) for healthy participants. Additionally, we presented pooled effect estimates using a fixed-effect model for analyses including more than one study (Supplement Table S21).

## Discussion

This systematic review summarises the most recent evidence on the effectiveness, immunogenicity and safety of HPV vaccines across a range of immunocompromised groups. Direct evidence comparing vaccinated immunocompromised groups to unvaccinated immunocompromised control groups with the same disease or condition (comparison 1) was rare and derived from NRSI (n = 2). Most included studies provided indirect evidence across different vaccinated immunocompromised groups (comparison 2; n = 2) or compared vaccinated immunocompromised groups to vaccinated healthy control groups (comparison 3; n = 15). Six studies provided single-arm data on safety.

Only one case–control study presented data on precancer and cancer of the cervix (i.e. CIN 2+ and CIN 3+) in women having received a catch-up vaccination at age 18–26, showing RRs near the null effect, although the certainty of evidence was very low due to serious risk of bias and considerable imprecision.^38^ None of the studies reported outcomes on precancers or cancers of the vulva, vagina, penis, or anus, oropharyngeal cancer, HPV infection, HPV-related mortality, or anogenital warts. Most studies assessed only immunogenicity outcomes (i.e. seropositivity rates and GMRs of antibody titres). Overall, the CoE was low to very low across groups and outcomes, primarily downgraded due to (very) serious risk of bias and (considerable) imprecision. However, seropositivity rates were high across most immunocompromised groups and time points.^39^, ^40^, ^41^, ^42^, ^43^^,^^45^, ^46^, ^47^, ^48^, ^49^, ^50^, ^51^, ^52^^,^^54^ GMRs of antibody titres varied between immunocompromised groups and healthy participants (comparison 3), with analyses showing both, potentially decreased or increased antibody titres compared to those of healthy participants. One study on solid organ transplant recipients suggested that there may be reduced seropositivity and antibody titres compared to healthy participants. However, the evidence was of very low certainty and inconsistent with findings from another study on solid organ transplant recipients, showing imprecise effects.^42^ Immunogenicity markers must be interpreted cautiously, particularly for immunocompromised individuals, as no antibody titre thresholds have been established that correlate with protection against HPV-associated cancers.^62^ Thus, higher antibody titres or seropositivity rates do not necessarily indicate greater protection. Despite the efforts of the World Health Organisation to standardise assays and protocols for antibody measurement,^63^ methodological inconsistencies persist across studies. Overall, the clinical relevance of immunogenicity outcomes remains uncertain, as there are no validation studies confirming immunogenicity as a reliable surrogate endpoint for patient-relevant outcomes. Furthermore, immunogenicity markers are influenced by various factors, including the underlying clinical conditions that affect the immune system, different immunosuppressive medications, vaccine timing, and prior exposure to HPV.^62^ Given the range of immunocompromised participants, with vaccine-induced immune responses influenced by unique factors, findings should not be generalised across groups.

Most studies included in this review reported immunogenicity data within 7 months after the initial vaccination, limiting insight into the durability of the immune response. However, some studies also reported extended follow-ups (i.e. 12 months or longer after initial vaccination), showing comparable immunogenicity outcomes.^47^^,^^49^^,^^51^^,^^52^ In addition, Mok et al. presented sustained immunogenicity data at five years in SLE participants, supported by similar findings from the single-arm study of MacIntyre et al. in a mixed immunocompromised population (i.e. haematological stem cell, liver and kidney transplantation recipients, JIA and IBD). However, long-term follow-up data are rare and should be interpreted with caution due to the limitations of the study design and inherent risk of bias. Based on our literature search for ongoing studies registered on ClinicalTrials.gov (Supplement Table S6), several additional NRSI are expected to be published in the coming years, which will expand the current evidence base. However, most of these upcoming studies are expected to focus on immunogenicity outcomes rather than efficacy and effectiveness outcomes.

This is the first comprehensive systematic review on HPV vaccination efficacy, effectiveness, immunogenicity and safety across a broad range of non-HIV immunocompromised individuals. The previously published narrative review of Garland et al. concluded that HPV vaccination is highly immunogenic and safe for immunocompromised participants; however, the review is primarily based on data from people living with HIV, with limited assessment of generalisability and study quality.^24^

The review of Vinkenes et al. included three studies on solid organ transplant recipients but found the evidence inconclusive due to small sample sizes and imprecise estimates.^22^ Our review adds two NRSI with control group on solid organ transplant recipients published in 2023 and 2024,^42^^,^^49^ but the added evidence does not resolve the uncertainties for this population. To date, most evidence on the effect of HPV vaccination in immunocompromised individuals is based on participants with HIV. Staadegard et al. identified 18 studies, including seven RCTs, showing a robust immune response in HIV participants, though data on efficacy and effectiveness outcomes were scarce. Further, the studies included in the review found only a few serious adverse events, and the HPV vaccine was deemed safe.^19^ Overall, these findings are consistent with our results.

Generalisability regarding different immunocompromised groups, types of immunosuppressive medications, demographic characteristics such as age, sex, and HPV vaccine types remains uncertain, as there were insufficient data to conduct meaningful subgroup analyses. However, some studies on solid organ transplant recipients suggested suboptimal immunogenicity results, compared to other populations, though effect estimates were often imprecise with confidence intervals frequently overlapping the null effect (RR = 1, GMR = 1).^39^^,^^40^^,^^42^^,^^49^ Only the study of Miyaji et al. reported in kidney, kidney and pancreas, liver, lung and heart transplant recipients precise effect estimates, yet the results were of very low certainty.^42^ Data from single-arm studies support suboptimal immunogenicity in solid organ transplant recipients.^56^^,^^58^

Most participants in the included studies received immunosuppressive treatments at baseline (Supplement Tables S9 and S20), but subgroup analyses were not feasible, limiting the assessment of specific immunosuppressive medications. Studies predominantly enrolled younger and female participants. Only three studies with healthy control groups included male participants^50^^,^^53^^,^^54^ but these either provided imprecise effect estimates or did not report detailed subgroup analyses. Three studies enrolled mostly adults over 25 years with SLE or a history of solid organ transplantation. Adults with SLE showed seropositivity rates comparable to those of healthy controls and other immunocompromised groups, whereas solid organ transplant recipients generally indicated lower immunogenicity.^41^^,^^51^ Results were also consistent across age groups 9–15 and 16–26 years.^40^^,^^50^ However, we assessed the certainty of the data presented in this review to be low to very low, generally deeming it insufficient for groups outside the scope of current HPV vaccine programmes.

Most studies reported data on the quadrivalent HPV vaccine; few studies evaluated the bivalent or nonavalent vaccines. However, studies reported broadly consistent findings on immunogenicity and serious adverse events across vaccine types.

We cannot rule out limitations within our review process. While the possibility of missing studies exists, we consider this unlikely, given our thorough literature search, including the review of reference lists from included studies and already published systematic reviews, supplemented by a search for registered studies. We excluded studies on HIV and other infectious diseases (e.g. malaria or helminthiasis) to maintain a focused research question. Additionally, baseline differences such as prior HPV vaccination or pre-existing infections may have influenced outcomes and cannot be excluded.

Risk of bias assessments inherently involve some subjectivity and other reviewers might have come to different conclusions. To mitigate this, paired reviewers conducted independent assessments. Importantly, evidence on efficacy and effectiveness outcomes (e.g. precancer or cancer of the cervix) is lacking. Most available evidence is limited to immunogenicity markers from NRSI with serious to critical risk of bias. Due to the insufficient data, we were not able to conduct subgroup analyses as pre-planned.

Most analyses in this review are descriptive, presenting groups with different underlying diseases separately. However, we were able to present a small number of pooled analyses for SLE, JIA, and solid organ transplant recipients. Although immunosuppressive treatments were generally comparable across these studies, other known and unknown factors, such as differences in dosing, combinations of treatments and demographic characteristics like age or sex, may have contributed to considerable clinical heterogeneity, limiting the generalisability and interpretability of pooled analyses. In addition, the underlying mechanisms of impaired vaccine responses are complex and not completely understood. Moreover, all effect estimates presented in this review are of low to very low certainty. Consequently, the true effect may be (low certainty) or is likely to be (very low certainty) substantially different from the effect estimates presented, so firm conclusions cannot be drawn. These findings should therefore be interpreted with caution and cannot be generalised. In summary, HPV vaccination appears to be immunogenic and generally safe for immunocompromised individuals, although the evidence is of low to very low CoE. To date, there is a lack of data on efficacy and effectiveness outcomes, such as HPV-associated precancers and cancers. High-quality studies addressing these endpoints are needed, ideally RCTs, but also well-conducted observational studies (e.g. cohort or registry-based studies), comparing vaccinated and unvaccinated immunocompromised individuals. Future research should also distinguish between the variety of immunocompromised groups and subgroups, including differences in specific immunosuppressive treatments, dosing, age and sex.

## Contributors

PK, WS, JJM, and TH conceptualised the review methodology. YC conceptualised the search strategy. PK, WS, LG, AT conducted the title and abstract screening. PK and WS conducted the full-text screening and disagreements were resolved by LG or JJM. PK, WS, LG, HS conducted data extraction and disagreements were resolved by JJM. PK and LG conducted the risk of bias assessment and WS resolved conflicts. PK and WS conducted the CoE assessment using GRADE. WS and PK conducted the statistical analyses. PK conceptualised the first draft of this review. WS, LG, HS, YC, MRM, MA, MB, PHA, DK, JL, SR, LS, BS, RT, AT, VU, SV, DZ, KAA, KO, TH, JJM provided feedback on the review findings and review draft. MRM, MB, PHA, DK, JL, SR, LS, BS, RT, AT, VU, SV, DZ, TH, JJM gave clinical and conceptual input throughout the review process. All authors read and approved the final version of the manuscript. PK and WS had direct access to and verified the underlying data reported in the manuscript.

## Data sharing statement

Data extracted for this study are available in the Supplementary Materials. Analysis code and example input datasets to reproduce Fig. 3A and Supplement Figure S2A are available in the Open Science Framework repository: osf.io/eh23s.

## Declaration of interests

The Cancer Epidemiology Research Program, with which MB is affiliated, has received sponsorship for grants from Merck Sharp & Dohme. We declare no competing interests for the remaining authors.
