# Supplementary material for: Effectiveness, immunogenicity and safety of human papillomavirus vaccination in non-HIV immunocompromised individuals: a systematic review
Source: eClinicalMedicine. 2026 Apr 11;94:103865. doi: 10.1016/j.eclinm.2026.103865 (PMC13091942; doi:10.1016/j.eclinm.2026.103865)
Supplement: Supplementary Tables and Figures [file mmc1.docx]

**Supplementary material**

[List of Tables 2](#_Toc224663980)

[Search strategies 3](#_Toc224663981)

[Eligibility criteria 9](#_Toc224663982)

[Registered studies 12](#_Toc224663983)

[List of included studies 13](#_Toc224663984)

[Excluded references by full text 16](#_Toc224663985)

[Risk of Bias assessments (ROBINS-I): comparisons 1-3 32](#_Toc224663986)

[Additional study characteristics: comparisons 1-3 47](#_Toc224663987)

[Antibody titres: comparison 2 55](#_Toc224663988)

[Serious adverse events: comparisons 2-3 56](#_Toc224663989)

[Local, systemic and additional adverse events: comparisons 2-3 and single-arm studies 58](#_Toc224663990)

[Additional summary of findings tables: comparison 3 75](#_Toc224663991)

[Antibody titres: comparison 3 80](#_Toc224663992)

[Additional study characteristics: single-arm studies 88](#_Toc224663993)

[Sensitivity analyses: comparisons 2-3 90](#_Toc224663994)

[Additional analyses: comparison 3 91](#_Toc224663995)

# **List of Tables**

[Supplement Table S1: Search strategy: Ovid MEDLINE(R) 3](#_Toc224637745)

[Supplement Table S2: Search strategy: Ovid Embase 4](#_Toc224637746)

[Supplement Table S3: Search strategy: Cochrane CENTRAL 6](#_Toc224637747)

[Supplement Table S4: Search strategy: ClinicalTrials.gov 8](#_Toc224637748)

[Supplement Table S5: Detailed eligibility criteria 9](#_Toc224637749)

[Supplement Table S6: Registered studies without corresponding publication 12](#_Toc224637750)

[Supplement Table S7: References excluded by full-text 16](#_Toc224637751)

[Supplement Table S8: Risk of Bias (ROBINS-I) 32](#_Toc224637752)

[Supplement Table S9: Additional study characteristics 47](#_Toc224637753)

[Supplement Table S10: Comparison 2 - GMT and GMR of HPV 16 at 7 months 55](#_Toc224637754)

[Supplement Table S11: Comparison 2 - GMT and GMR of HPV 18 at 7 months 55](#_Toc224637755)

[Supplement Table S12: Serious adverse events 56](#_Toc224637756)

[Supplement Table S13: Local, systemic and additional adverse events across vaccine types 58](#_Toc224637757)

[Supplement Table S14: Summary of findings, vaccinated immunocompromised group vs. vaccinated healthy control group (comparison 3) Outcome: Seropositivity, 12 months and more 75](#_Toc224637758)

[Supplement Table S15: Summary of findings, vaccinated immunocompromised group vs. vaccinated healthy control group (comparison 3) Outcome: Geometric mean ratio (GMR) 77](#_Toc224637759)

[Supplement Table S16: Comparison 3 - GMT and GMR of HPV 16 at 7 months 80](#_Toc224637760)

[Supplement Table S17: Comparison 3 - GMT and GMT of HPV 18 at 7 months 83](#_Toc224637761)

[Supplement Table S18: Comparison 3 - GMT and GMR of HPV 16 at 12 months and more 86](#_Toc224637762)

[Supplement Table S19: Comparison 3 - GMT and GMR of HPV 18 at 12 months and more 87](#_Toc224637763)

[Supplement Table S20: Study characteristics (single-arm studies) 88](#_Toc224637764)

[Supplement Table S21: Sensitivity analysis 90](#_Toc224637765)

**List of Figures**

[Supplement Figure S1: Seropositivity of HPV 16 and 18 at 12 months and more (comparison 3) 91](#_Toc224637698)

[Supplement Figure S2: GMR of HPV 16 and 18 at 7 months (comparison 3) 93](#_Toc224637699)

[Supplement Figure S3: GMR of HPV 16 and 18 at 12 months and more (comparison 3) 94](#_Toc224637700)

# **Search strategies**

**Supplement Table S1: Search strategy: Ovid MEDLINE(R) ALL 1946 to May 03, 2024 (searched November 26, 2025)**

| # | Search | Results |
| --- | --- | --- |
| 1 | exp Papillomavirus Vaccines/ | 11774 |
| 2 | ((hpv* or human papilloma virus* or human papiloma virus* or human papillomavirus* or human papilomavirus*) and (vaccin* or immuni*)).ti,kf. | 12659 |
| 3 | ((hpv* or human papilloma virus* or human papiloma virus* or human papillomavirus* or human papilomavirus*) adj3 (vaccin* or immuni*)).ab. | 14918 |
| 4 | (cecolin* or cervarix* or gardasil*).mp. | 855 |
| 5 | 1 or 2 or 3 or 4 | 19452 |
| 6 | exp Papillomavirus Infections/ | 47940 |
| 7 | exp Papillomaviridae/ | 41582 |
| 8 | (hpv* or human papilloma virus* or human papiloma virus* or human papillomavirus* or human papilomavirus*).ti,ab,kf. | 74901 |
| 9 | Vaccination/ or exp Immunization/ | 228477 |
| 10 | (6 or 7 or 8) and 9 | 7314 |
| 11 | 5 or 10 | 20069 |
| 12 | Cancer survivors/ | 12742 |
| 13 | Cancer Care Facilities/ | 6263 |
| 14 | ((cancer* or tumor* or tumour* or neoplas* or malignan* or oncolog* or carcinoma* or adenocarcinoma* or choriocarcinoma* or leukemia* or leukaemia* or metastat* or sarcoma* or teratoma* or hodgkin* or nonhodgkin* or lymphoma* or melanoma* or myeloma* or palliative) adj2 (survivor* or survival or treatment* or care)).ti,ab,kf. | 414423 |
| 15 | exp Transplantation/ | 602718 |
| 16 | ((organ or tissue or heart or kidney or liver or lung or pancreas) adj transplant*).ti,ab,kf. | 222697 |
| 17 | exp Primary Immunodeficiency Diseases/ | 16188 |
| 18 | exp Phagocyte Bactericidal Dysfunction/ or exp Lymphopenia/ or exp Dysgammaglobulinemia/ or Agammaglobulinemia/ or Common Variable Immunodeficiency/ | 24700 |
| 19 | exp Autoimmune Diseases/ or exp Immunocompromised Host/ or exp Hereditary Autoinflammatory Diseases/ | 630663 |
| 20 | (immunocompromis* or autoinflammat* or autoimmune* or asplenia or behcet or cryopyrin* or familial mediterranean fever or mevalonate kinase deficien* or ataxia telangiectasia or bloom syndrome or Chediak Higashi or Leukocyte-Adhesion or Wiskott-Aldrich or rheumatoid arthritis or systemic lupus erythematosus or sle or myasthenia gravis or multiple sclerosis).ti,ab,kf. | 563307 |
| 21 | exp Immunosuppressive Agents/ | 360545 |
| 22 | exp Immunosuppression Therapy/ | 69087 |
| 23 | Plasmapheresis/ | 9460 |
| 24 | Intravenous Immunoglobulins/ or Immunoglobulins/ | 61716 |
| 25 | Lymphatic Irradiation/ | 1382 |
| 26 | Celecoxib/ or Glatiramer Acetate/ or Minocycline/ or Thalidomide/ or Interleukin 1 Receptor Antagonist Protein/ or Pentoxifylline/ or Glucocorticoid/ | 109044 |
| 27 | Immunogenicity/ | 4343 |
| 28 | (immunologic or immunosuppress* or immunomodulat* or plasmapheresis or immunoglobulin* or (lymph* adj irradiation) or celecoxib or glatiramer or minocycline or thalidomide or anakinra or NP001 or pentoxiphylline or pentoxifylline).ti,ab,kf. | 576700 |
| 29 | exp Hematologic Diseases/ | 847152 |
| 30 | hematolog*.ti,ab,kf. | 153487 |
| 31 | exp Hematopoietic Stem Cell Mobilization/ | 4654 |
| 32 | exp Allograft/ or exp Allotransplantation/ or exp Myeloablative Agent/ or exp Bone Marrow Rescue/ | 13326 |
| 33 | exp Graft Versus Host Reaction/ | 0 |
| 34 | ((allogen* or bone or cell or blood or marrow) adj2 transplant*).ti,ab,kf. | 143449 |
| 35 | (myeloablative therapy or myeloablative agonist? or allograft? or haematopo?etic or hematopo?etic or haemopo?etic or hemopo?etic or haematocytopo?etic or hematocytopo?etic or HSCT or cotransplant* or coinfus* or co transplant* or co infus* or HLA matched or HLA identical or haploidentical).ti,ab,kf. | 243422 |
| 36 | 12 or 13 or 14 or 15 or 16 or 17 or 18 or 19 or 20 or 21 or 22 or 23 or 24 or 25 or 26 or 27 or 28 or 29 or 30 or 31 or 32 or 33 or 34 or 35 | 3443651 |
| 37 | 11 and 36 | 1606 |
| 38 | (animals not (humans and animals)).sh. | 5364406 |
| 39 | 37 not 38 | 1524 |

**Supplement Table S2: Search strategy: Ovid Embase 1974 to May 03 2024 (searched November 26, 2025)**

| # | Search | Results |
| --- | --- | --- |
| 1 | exp Papillomavirus Vaccines/ | 22192 |
| 2 | ((hpv* or human papilloma virus* or human papiloma virus* or human papillomavirus* or human papilomavirus*) and (vaccin* or immuni*)).ti,kf. | 17061 |
| 3 | ((hpv* or human papilloma virus* or human papiloma virus* or human papillomavirus* or human papilomavirus*) adj3 (vaccin* or immuni*)).ab. | 20334 |
| 4 | (cecolin* or cervarix* or gardasil*).mp. | 3915 |
| 5 | 1 or 2 or 3 or 4 | 30758 |
| 6 | exp Papillomavirus Infections/ | 54899 |
| 7 | exp Papillomaviridae/ | 78336 |
| 8 | (hpv* or human papilloma virus* or human papiloma virus* or human papillomavirus* or human papilomavirus*).ti,ab,kf. | 103761 |
| 9 | Vaccination/ or exp Immunization/ | 442994 |
| 10 | (6 or 7 or 8) and 9 | 20825 |
| 11 | 5 or 10 | 33920 |
| 12 | Cancer survivors/ | 44187 |
| 13 | Cancer Care Facilities/ | 57951 |
| 14 | ((cancer* or tumor* or tumour* or neoplas* or malignan* or oncolog* or carcinoma* or adenocarcinoma* or choriocarcinoma* or leukemia* or leukaemia* or metastat* or sarcoma* or teratoma* or hodgkin* or nonhodgkin* or lymphoma* or melanoma* or myeloma* or palliative) adj2 (survivor* or survival or treatment* or care)).ti,ab,kf. | 633936 |
| 15 | exp Transplantation/ | 1391056 |
| 16 | ((organ or tissue or heart or kidney or liver or lung or pancreas) adj transplant*).ti,ab,kf. | 385644 |
| 17 | exp Primary Immunodeficiency Diseases/ | 722067 |
| 18 | exp Phagocyte Bactericidal Dysfunction/ or exp Lymphopenia/ or exp Dysgammaglobulinemia/ or Agammaglobulinemia/ or Common Variable Immunodeficiency/ | 69401 |
| 19 | exp Autoimmune Diseases/ or exp Immunocompromised Host/ or exp Hereditary Autoinflammatory Diseases/ | 929346 |
| 20 | (immunocompromis* or autoinflammat* or autoimmune* or asplenia or behcet or cryopyrin* or familial mediterranean fever or mevalonate kinase deficien* or ataxia telangiectasia or bloom syndrome or Chediak Higashi or Leukocyte-Adhesion or Wiskott-Aldrich or rheumatoid arthritis or systemic lupus erythematosus or sle or myasthenia gravis or multiple sclerosis).ti,ab,kf. | 856709 |
| 21 | exp Immunosuppressive Agents/ | 1602784 |
| 22 | exp Immunosuppression Therapy/ | 308925 |
| 23 | Plasmapheresis/ | 45453 |
| 24 | Intravenous Immunoglobulins/ or Immunoglobulins/ | 150953 |
| 25 | Lymphatic Irradiation/ | 232473 |
| 26 | Celecoxib/ or Glatiramer Acetate/ or Minocycline/ or Thalidomide/ or Interleukin 1 Receptor Antagonist Protein/ or Pentoxifylline/ or Glucocorticoid/ | 259638 |
| 27 | Immunogenicity/ | 103171 |
| 28 | (immunologic or immunosuppress* or immunomodulat* or plasmapheresis or immunoglobulin* or (lymph* adj irradiation) or celecoxib or glatiramer or minocycline or thalidomide or anakinra or NP001 or pentoxiphylline or pentoxifylline).ti,ab,kf. | 836462 |
| 29 | exp Hematologic Diseases/ | 3232582 |
| 30 | hematolog*.ti,ab,kf. | 291321 |
| 31 | exp Hematopoietic Stem Cell Mobilization/ | 9309 |
| 32 | exp Allograft/ or exp Allotransplantation/ or exp Myeloablative Agent/ or exp Bone Marrow Rescue/ | 92950 |
| 33 | exp Graft Versus Host Reaction/ | 92349 |
| 34 | ((allogen* or bone or cell or blood or marrow) adj2 transplant*).ti,ab,kf. | 254066 |
| 35 | (myeloablative therapy or myeloablative agonist? or allograft? or haematopo?etic or hematopo?etic or haemopo?etic or hemopo?etic or haematocytopo?etic or hematocytopo?etic or HSCT or cotransplant* or coinfus* or co transplant* or co infus* or HLA matched or HLA identical or haploidentical).ti,ab,kf. | 385569 |
| 36 | 12 or 13 or 14 or 15 or 16 or 17 or 18 or 19 or 20 or 21 or 22 or 23 or 24 or 25 or 26 or 27 or 28 or 29 or 30 or 31 or 32 or 33 or 34 or 35 | 7827045 |
| 37 | 11 and 36 | 7828 |
| 38 | (exp animal/ or exp invertebrate/ or nonhuman/ or animal experiment/ or animal tissue/ or animal model/ or exp plant/ or exp fungus/) not (exp human/ or human tissue/) | 8461075 |
| 39 | 37 not 38 | 7206 |

**Supplement Table S3: Search strategy: Cochrane CENTRAL (via the Cochrane Register of Studies Online) (searched November 26, 2025)**

| # | Search | Results |
| --- | --- | --- |
| 1 | MESH DESCRIPTOR Papillomavirus Vaccines EXPLODE ALL AND CENTRAL:TARGET | 737 |
| 2 | ((hpv* or human papilloma virus* or human papiloma virus* or human papillomavirus* or human papilomavirus*) and (vaccin* or immuni*)):TI,KY  AND CENTRAL:TARGET | 1466 |
| 3 | ((hpv* or human papilloma virus* or human papiloma virus* or human papillomavirus* or human papilomavirus*) adj3 (vaccin* or immuni*)):AB  AND CENTRAL:TARGET | 1571 |
| 4 | (cecolin* or cervarix* or gardasil*):TI,AB,KY  AND CENTRAL:TARGET | 271 |
| 5 | #1 OR #2 OR #3 OR #4 | 1910 |
| 6 | MESH DESCRIPTOR Papillomavirus Infections EXPLODE ALL AND CENTRAL:TARGET | 1203 |
| 7 | MESH DESCRIPTOR Papillomaviridae EXPLODE ALL AND CENTRAL:TARGET | 1141 |
| 8 | (hpv* or human papilloma virus* or human papiloma virus* or human papillomavirus* or human papilomavirus*):TI,AB,KY  AND CENTRAL:TARGET | 4946 |
| 9 | MESH DESCRIPTOR Vaccination AND CENTRAL:TARGET | 4931 |
| 10 | MESH DESCRIPTOR Immunization EXPLODE ALL AND CENTRAL:TARGET | 8320 |
| 11 | (#6 OR #7 OR #8) AND (#9 OR #10) | 493 |
| 12 | #5 OR #11 | 1921 |
| 13 | MESH DESCRIPTOR Cancer survivors AND CENTRAL:TARGET | 1259 |
| 14 | MESH DESCRIPTOR Cancer Care Facilities AND CENTRAL:TARGET | 123 |
| 15 | ((cancer* or tumor* or tumour* or neoplas* or malignan* or oncolog* or carcinoma* or adenocarcinoma* or choriocarcinoma* or leukemia* or leukaemia* or metastat* or sarcoma* or teratoma* or hodgkin* or nonhodgkin* or lymphoma* or melanoma* or myeloma* or palliative) adj2 (survivor* or survival or treatment* or care)):TI,AB,KY  AND CENTRAL:TARGET | 53146 |
| 16 | MESH DESCRIPTOR Transplantation EXPLODE ALL AND CENTRAL:TARGET | 18861 |
| 17 | ((organ or tissue or heart or kidney or liver or lung or pancreas) adj transplant*):TI,AB,KY  AND CENTRAL:TARGET | 16695 |
| 18 | MESH DESCRIPTOR Primary Immunodeficiency Diseases EXPLODE ALL AND CENTRAL:TARGET | 268 |
| 19 | MESH DESCRIPTOR Phagocyte Bactericidal Dysfunction EXPLODE ALL AND CENTRAL:TARGET | 38 |
| 20 | MESH DESCRIPTOR Lymphopenia EXPLODE ALL AND CENTRAL:TARGET | 137 |
| 21 | MESH DESCRIPTOR Dysgammaglobulinemia EXPLODE ALL AND CENTRAL:TARGET | 17 |
| 22 | MESH DESCRIPTOR Agammaglobulinemia EXPLODE ALL AND CENTRAL:TARGET | 63 |
| 23 | MESH DESCRIPTOR Common Variable Immunodeficiency EXPLODE ALL AND CENTRAL:TARGET | 38 |
| 24 | MESH DESCRIPTOR Autoimmune Diseases EXPLODE ALL AND CENTRAL:TARGET | 28159 |
| 25 | MESH DESCRIPTOR Immunocompromised Host EXPLODE ALL AND CENTRAL:TARGET | 350 |
| 26 | MESH DESCRIPTOR Hereditary Autoinflammatory Diseases EXPLODE ALL AND CENTRAL:TARGET | 284 |
| 27 | (immunocompromis* or autoinflammat* or autoimmune* or asplenia or behcet or cryopyrin* or familial mediterranean fever or mevalonate kinase deficien* or ataxia telangiectasia or bloom syndrome or Chediak Higashi or Leukocyte-Adhesion or Wiskott-Aldrich or rheumatoid arthritis or systemic lupus erythematosus or sle or myasthenia gravis or multiple sclerosis):TI,AB,KY  AND CENTRAL:TARGET | 41562 |
| 28 | MESH DESCRIPTOR Immunosuppressive Agents EXPLODE ALL AND CENTRAL:TARGET | 27518 |
| 29 | MESH DESCRIPTOR Immunosuppression Therapy EXPLODE ALL AND CENTRAL:TARGET | 2972 |
| 30 | MESH DESCRIPTOR Plasmapheresis AND CENTRAL:TARGET | 333 |
| 31 | MESH DESCRIPTOR Immunoglobulins, Intravenous AND CENTRAL:TARGET | 1143 |
| 32 | MESH DESCRIPTOR Immunoglobulins AND CENTRAL:TARGET | 1519 |
| 33 | MESH DESCRIPTOR Lymphatic Irradiation AND CENTRAL:TARGET | 97 |
| 34 | MESH DESCRIPTOR Celecoxib AND CENTRAL:TARGET | 1234 |
| 35 | MESH DESCRIPTOR Glatiramer Acetate AND CENTRAL:TARGET | 469 |
| 36 | MESH DESCRIPTOR Minocycline AND CENTRAL:TARGET | 684 |
| 37 | MESH DESCRIPTOR Thalidomide AND CENTRAL:TARGET | 1157 |
| 38 | MESH DESCRIPTOR Interleukin 1 Receptor Antagonist Protein AND CENTRAL:TARGET | 459 |
| 39 | MESH DESCRIPTOR Pentoxifylline AND CENTRAL:TARGET | 3141 |
| 40 | (immunologic or immunosuppress* or immunomodulat* or plasmapheresis or immunoglobulin* or (lymph* adj irradiation) or celecoxib or glatiramer or minocycline or thalidomide or anakinra or NP001 or pentoxiphylline or pentoxifylline):TI,AB,KY  AND CENTRAL:TARGET | 42970 |
| 41 | MESH DESCRIPTOR Hematologic Diseases EXPLODE ALL AND CENTRAL:TARGET | 20977 |
| 42 | hematolog*:TI,AB,KY  AND CENTRAL:TARGET | 23517 |
| 43 | MESH DESCRIPTOR Hematopoietic Stem Cell Mobilization EXPLODE ALL AND CENTRAL:TARGET | 361 |
| 44 | MESH DESCRIPTOR Allografts EXPLODE ALL AND CENTRAL:TARGET | 385 |
| 45 | MESH DESCRIPTOR Vascularized Composite Allotransplantation EXPLODE ALL AND CENTRAL:TARGET | 7 |
| 46 | ((allogen* or bone or cell or blood or marrow) adj2 transplant*):TI,AB,KY  AND CENTRAL:TARGET | 14267 |
| 47 | (myeloablative therapy or myeloablative agonist? or allograft? or haematopo?etic or hematopo?etic or haemopo?etic or hemopo?etic or haematocytopo?etic or hematocytopo?etic or HSCT or cotransplant* or coinfus* or co transplant* or co infus* or HLA matched or HLA identical or haploidentical):TI,AB,KY  AND CENTRAL:TARGET | 13804 |
| 48 | #13 OR #14 OR #15 OR #16 OR #17 OR #18 OR #19 OR #20 OR #21 OR #22 OR #23 OR #24 OR #25 OR #26 OR #27 OR #28 OR #29 OR #30 OR #31 OR #32 OR #33 OR #34 OR #35 OR #36 OR #37 OR #38 OR #39 OR #40 OR #41 OR #42 OR #43 OR #44 OR #45 OR #46 OR #47 | 223399 |
| 49 | #12 AND #48 | 173 |

**Supplement Table S4: Search strategy: ClinicalTrials.gov (searched November 26, 2025)**

| **Conditions:** immunocompromised OR autoimmune OR sle OR hematology OR transplant OR ((cancer OR tumor OR carcinoma OR adenocarcinoma OR metastatic OR melanoma) AND (survivor OR survival OR treatment OR care) | 160 |
| --- | --- |
| **Intervention/treatment:** (hpv OR human papilloma virus OR human papiloma virus OR human papillomavirus OR human papilomavirus) AND (vaccine OR vaccination OR immune OR immunization) |  |

# **Eligibility criteria**

**Supplement Table S5: Detailed eligibility criteria**

| **Types of studies** | Randomised controlled trials (RCTs)  Other study designs also included were defined as:   - Non-randomised studies of interventions (NRSI)^^[[1]](#footnote-1)^^ in which participants (individuals or clusters of individuals) are allocated to different groups (intervention and control group) using methods that are not random - Observational studies, i.e. prospective and retrospective cohort studies with a control group and case control studies. In observational studies the allocation to a group is determined by factors outside the investigator's control which can bias the selections of participants into the study - Single-arm studies (i.e. cohort studies that are sampled based on HPV vaccination status) |
| --- | --- |
| **Types of participants** | Studies investigating individuals of any age and sex with one or more of the following pre-specified conditions were included:   - - Conditions (with or without therapy):     - Primary immunodeficiencies     - Autoinflammatory diseases (e.g. Familial Mediterranean Fever)     - Autoimmune diseases (e.g. Rheumatoid arthritis)     - Haematological diseases     - Oncological diseases     - Organ transplantation     - Patients on dialysis     - Chronic kidney diseases     - Stem cell therapy     - Any other secondary immunodeficiency (excluding HIV and other infectious diseases, such as malaria or helminthiasis)   - Under therapy: Immunomodulatory or immunosuppressive drugs (e.g. glucocorticoids)   Studies were also included if the population of interest (i.e. immunocompromised individuals) comprised a minimum of 80% of the entire study population or if the studies reported results of immunocompromised individuals separately.  Studies focusing on individuals with HIV and other infectious diseases were excluded. |
| **Types of Intervention** | (i) nonavalent HPV vaccine (Gardasil 9, 9vHPV), (ii) quadrivalent HPV vaccine (Gardasil, 4vHPV) and (iii) bivalent HPV vaccine (Cervarix, 2vHPV). |
| **Type of comparison** | Studies that compared a **vaccinated immunocompromised group** with any of the following groups were included:  *Unvaccinated immunocompromised control group with the same disease or condition (comparison 1)*   - No vaccination - Placebo (containing no active agent, only the adjuvant of the HPV vaccine) - A non-HPV vaccine   In addition, the following control groups were considered in the review:  *Other vaccinated immunocompromised control group with a different disease or condition that affects the immune system (comparison 2)*   - Other immunocompromised group (as defined in section 3.1.2) who received the HPV vaccination   *Vaccinated healthy control group (comparison 3)*   - Healthy controls from the general population that are not immunocompromised (as defined in “types of participants) who received the HPV vaccination   Additionally, safety/adverse outcomes data of studies without independent comparison group were also descriptively reported:   - No independent control (e.g. before and after comparisons within the same individuals)   No control group (non-comparative, single-arm studies) |
| **Outcomes** | **1. Patient relevant outcomes:**   - Precancer or cancer of the cervix (including the histopathologically confirmed cervical lesions as defined by the WHO [e.g. CIN 2+])   - By HPV type: for disease-related HPV types 16/18 - Precancers or cancers of the vulva (e.g. vulvar intraepithelial neoplasia [VIN], vagina (e.g. vaginal intraepithelial neoplasia [VaIN]), penis (e.g. penile intraepithelial neoplasia [PeIN]) or anus (e.g. anal intraepithelial neoplasia [AIN]), and oropharyngeal cancer   - By HPV type: for disease-related HPV types 16/18 - Anogenital warts (as reported by the study authors)   - By HPV type: for disease-related HPV types 6/11 - HPV infection (incident^^[[2]](#footnote-2)^^and persistent^^[[3]](#footnote-3)^^ infections)   - By HPV type: for disease-related HPV types 16/18 - Mortality caused by HPV-related cancers   **2. Immunogenicity parameters of interest:**   - Seropositivity rates (as defined by clinical trials) - Geometric mean ratio (GMR) to measure the antibody response   **3. Safety/adverse outcomes:**   - Any adverse events (as defined by clinical trials) - Any serious adverse events (as defined by clinical trials) - Specific adverse effects related to the HPV vaccine: Local events and systemic events (as defined by clinical trials) |

AIN: anal intraepithelial neoplasia; CIN 2+: cervical intraepithelial neoplasia grade 2+; CIN 3+: cervical intraepithelial neoplasia grade 3+; GMR: geometric mean ratio; HPV: human papillomavirus; NRSI: non-randomised studies of interventions; PeIN: penile intraepithelial neoplasia; RCT: randomized controlled trial; VIN: vulvar intraepithelial neoplasia; VaIN: vaginal intraepithelial neoplasia; WHO: World Health Organization

# **Registered studies**

**Supplement Table S6: Registered studies without corresponding publication**

| **Study ID/ country** | **Register link** | **Type of study/ Actual or planed sample size** | **Status** |
| --- | --- | --- | --- |
| NCT01687192 / (PRIMAVERA) France | <https://clinicaltrials.gov/study/NCT01687192> | NRSI / 37 | completed - no results posted; actual study completion date: 10/2016 |
| NCT00505063/ USA | https://clinicaltrials.gov/study/NCT00505063 | NRSI / 76 | completed - no results posted; actual study completion date: 06/2025 |
| NCT03180359 / (COVAGREF) France | https://clinicaltrials.gov/study/NCT03180359 | NRSI / 55 | completed - no results posted; actual study completion date: 10/2019 |
| NCT03023631 / USA | <https://clinicaltrials.gov/study/NCT03023631> | NRSI / 48 | active - not recruiting; estimated study completion date: 07/2026 |
| NCT01896986 / Australia | <https://clinicaltrials.gov/study/NCT01896986> | NRSI / 37 | terminated - results posted; actual study completion date: 03/2012 |
| NCT03519464 / USA | <https://clinicaltrials.gov/study/NCT03519464> | NRSI / 54 | active – not recruiting; estimated study completion date: 12/2026 |
| NCT05557370 / USA | <https://clinicaltrials.gov/study/NCT05557370> | NRSI / 30 | active – not recruiting; estimated study completion date: 12/2035 |
| NCT00964210 / Australia | <https://clinicaltrials.gov/study/NCT00964210> | NRSI / 240 | completed - no results posted; actual study completion date: 04/2011 |
| NCT03036930 / USA | <https://clinicaltrials.gov/study/NCT03036930> | NRSI / 51 | active - not recruiting; estimated study completion date: 03/2026 |
| NCT05439083 / Spain | <https://clinicaltrials.gov/study/NCT05439083> | NRSI / 120 | active – recruiting; estimated study completion date: 03/2025 |
| NCT03100682 / (HPVaxResponse Study) Germany | <https://clinicaltrials.gov/study/NCT03100682> | NRSI / 140 | unknown; estimated study completion date: 12/2022 |
| NCT00573651 / (CHASE) USA | <https://clinicaltrials.gov/study/NCT00573651> | NRSI / 43 | completed - results posted; actual study completion date: 10/2014 |
| NCT06793410 / (ALLO-HPV) Sweden | https://www.clinicaltrials.gov/study/NCT06793410 | RCT / 100 | active – recruiting; estimated study completion date: 10/2029 |
| NCT01298869 / Thailand | https://clinicaltrials.gov/study/NCT01298869 | NRSI / 60 | unknown; estimated study completion date: 12/2014 |
| NCT01034358 / USA | <https://clinicaltrials.gov/study/NCT01034358> | NRSI / 15 | completed - results posted; actual study completion date: 06/2011 |

NRSI: non-randomised studies of interventions

# **List of included studies**

**Vaccinated immunocompromised group vs. unvaccinated immunocompromised control group with the same disease or condition (comparison 1)**

1. Grönlund O, Herweijer E, Sundström K, Arnheim-Dahlström L. Incidence of new-onset autoimmune disease in girls and women with pre-existing autoimmune disease after quadrivalent human papillomavirus vaccination: a cohort study. J Intern Med. 2016;280(6):618-26.
2. Silverberg MJ, Leyden WA, Lam JO, Chao CR, Gregorich SE, Huchko MJ, et al. Effectiveness of 'catch-up' human papillomavirus vaccination to prevent cervical neoplasia in immunosuppressed and non-immunosuppressed women. Vaccine. 2020;38(29):4520-3.

**Vaccinated immunocompromised group vs. other vaccinated immunocompromised control group with a different disease or condition that affects the immune system (comparison 2)**

1. Nailescu C, Nelson RD, Verghese PS, Twombley KE, Chishti AS, Mills M, et al. Human Papillomavirus Vaccination in Male and Female Adolescents Before and After Kidney Transplantation: A Pediatric Nephrology Research Consortium Study. Front Pediatr. 2020;8:46.
2. Nelson DR, Neu AM, Abraham A, Amaral S, Batisky D, Fadrowski JJ. Immunogenicity of Human Papillomavirus Recombinant Vaccine in Children with CKD. Clinical journal of the American Society of Nephrology: Clin J Am Soc Nephrol. 2016;11(5):776-84.

**Vaccinated immunocompromised group vs. vaccinated healthy control group (comparison 3)**

1. Alter BP, Giri N, Pan Y, Savage SA, Pinto LA. Antibody response to human papillomavirus vaccine in subjects with inherited bone marrow failure syndromes. Vaccine. 2014;32(10):1169-73.
2. Dhar JP, Essenmacher L, Dhar R, Magee A, Ager J, Sokol RJ. The safety and immunogenicity of Quadrivalent HPV (qHPV) vaccine in systemic lupus erythematosus. Vaccine. 2017;35(20):2642-6.

*Secondary publication:
Dhar JP, Essenmacher L, Dhar R, Magee A, Ager J, Sokol RJ. The effect of history of abnormal pap smear or preceding HPV infection on the humoral immune response to Quadrivalent Human Papilloma virus (qHPV) vaccine in women with systemic lupus erythematosus. Hum Vaccin Immunother. 2018;14(9):2318-22.*

1. Esposito S, Corona F, Barzon L, Cuoco F, Squarzon L, Marcati G, et al. Immunogenicity, safety and tolerability of a bivalent human papillomavirus vaccine in adolescents with juvenile idiopathic arthritis. Expert Rev Vaccines. 2014;13(11):1387-93.
2. Grein IHR, Pinto NF, Lobo A, Groot N, Sztajnbok F, da Silva CAA, et al. Safety and immunogenicity of the quadrivalent human papillomavirus vaccine in patients with childhood systemic lupus erythematosus: a real-world interventional multi-centre study. Lupus. 2020a;29(8):934-42.
3. Grein IHR, Pinto NBF, Groot N, Martins CB, Lobo A, Aikawa NE, et al. Safety and immunogenicity of the quadrivalent human papillomavirus vaccine in patients with juvenile dermatomyositis: a real-world multicentre study. Pediatr Rheumatol Online J. 2020b;18(1):87.
4. Gomez-Lobo V, Whyte T, Kaufman S, Torres C, Moudgil A. Immunogenicity of a prophylactic quadrivalent human papillomavirus L1 virus-like particle vaccine in male and female adolescent transplant recipients. Pediatr Transplant. 2014;18(3):310-5.
5. Heijstek MW, Scherpenisse M, Groot N, Tacke C, Schepp RM, Buisman AM, et al. Immunogenicity and safety of the bivalent HPV vaccine in female patients with juvenile idiopathic arthritis: A prospective controlled observational cohort study. Ann Rheum Dis. 2014;73(8):1500-7.
6. Jacobson DL, Bousvaros A, Ashworth L, Carey R, Shrier LA, Burchett SK, et al. Immunogenicity and tolerability to human papillomavirus-like particle vaccine in girls and young women with inflammatory bowel disease. Inflamm Bowel Dis. 2013;19(7):1441-9.
7. Kitano T, Schwartz KL, Abdulnoor M, Garfield H, Booran NK, Avitzur Y, et al. Immunogenicity of a quadrivalent human papillomavirus vaccine in pediatric kidney and liver transplant recipients. Pediatr Transplant. 2023;27(3):e14476.
8. Landier W, Bhatia S, Wong FL, York JM, Flynn JS, Henneberg HM, et al. Immunogenicity and safety of the human papillomavirus vaccine in young survivors of cancer in the USA: a single-arm, open-label, phase 2, non-inferiority trial. Lancet Child Adolesc Health. 2022;6(1):38-48.
9. Miyaji KT, Infante V, Picone CM, et al. Quadrivalent HPV (4vHPV) vaccine immunogenicity and safety in women using immunosuppressive drugs due to solid organ transplant. Front Cell Infect Microbiol. 2024; 14: 1452916.

*Secondary publication:
Moreira Dos Santos LZ, Rodrigues CCM, Miyaji KT, et al. Immunogenicity and safety of the fourth dose of quadrivalent human papillomavirus (HPV) vaccine in immunosuppressed women who did not seroconvert after three doses. Front Cell Infect Microbiol 2024; 14: 1451308.*

1. Mok CC, Ho LY, Fong LS, To CH. Immunogenicity and safety of a quadrivalent human papillomavirus vaccine in patients with systemic lupus erythematosus: a case-control study. Ann Rheum Dis. 2013;72(5):659-64.

*Secondary publication:
Mok CC, Ho LY, To CH. Long-term immunogenicity of a quadrivalent human papillomavirus vaccine in systemic lupus erythematosus. Vaccine. 2018;36(23):3301-7.*

1. Nelson DR, Neu AM, Abraham A, Amaral S, Batisky D, Fadrowski JJ. Immunogenicity of Human Papillomavirus Recombinant Vaccine in Children with CKD. Clinical journal of the American Society of Nephrology: Clin J Am Soc Nephrol. 2016;11(5):776-84.
2. Sauter SL, Zhang X, Romick-Rosendale L, Wells SI, Myers KC, Brusadelli MG, et al. Human papillomavirus oral-and seropositivity in fanconi anemia. Cancers. 2021;13(6):1-18.

*Secondary publication:
Katzenellenbogen RA, Carter JJ, Stern JE, Butsch Kovacic MS, Mehta PA, Sauter SL, et al. Skin and mucosal human papillomavirus seroprevalence in persons with Fanconi Anemia. Clinical and vaccine immunology: CVI. 2015;22(4):413-20.*

1. Stratton P, Battiwalla M, Tian X, Abdelazim S, Baird K, Barrett AJ, et al. Immune Response Following Quadrivalent Human Papillomavirus Vaccination in Women After Hematopoietic Allogeneic Stem Cell Transplant: A Nonrandomized Clinical Trial. JAMA Oncol. 2020;6(5):696-705.

**Single-arm studies**

1. Boey L, Curinckx A, Roelants M, Derdelinckx I, Van Wijngaerden E, De Munter P, Vos R, Kuypers D, Van Cleemput J, Vandermeulen C. Immunogenicity and Safety of the 9-Valent Human Papillomavirus Vaccine in Solid Organ Transplant Recipients and Adults Infected With Human Immunodeficiency Virus (HIV). Clin Infect Dis. 2021 Aug 2;73(3):e661-e671.
2. Liu EY, Smith LM, Ellis AK, Whitaker H, Law B, Kwong JC, et al. Quadrivalent human papillomavirus vaccination in girls and the risk of autoimmune disorders: the Ontario Grade 8 HPV Vaccine Cohort Study. CMAJ. 2018;190(21):E648-E55.
3. Kumar D, Unger ER, Panicker G, Medvedev P, Wilson L, Humar A. Immunogenicity of quadrivalent human papillomavirus vaccine in organ transplant recipients. *Am J Transplant*. 2013;13(9):2411-7.
4. MacIntyre CR, Shaw P, Mackie FE, Boros C, Marshall H, Barnes M, et al. Immunogenicity and persistence of immunity of a quadrivalent Human Papillomavirus (HPV) vaccine in immunocompromised children. Vaccine. 2016;34(36):4343-50.

*Secondary publication:*

*MacIntyre CR, Shaw PJ, Mackie FE, Boros C, Marshall H, Seale H, et al. Long term follow up of persistence of immunity following quadrivalent Human Papillomavirus (HPV) vaccine in immunocompromised children. Vaccine. 2019;37(37):5630-6.*

1. Praditpornsilpa K, Kingwatanakul P, Deekajorndej T, Rianthavorn P, Susantitaphong P, Katavetin P, et al. Immunogenicity and safety of quadrivalent human papillomavirus types 6/11/16/18 recombinant vaccine in chronic kidney disease stage IV, V and VD. Nephrol Dial Transplant. 2017;32(1):132-6.
2. Soybilgic A, Onel KB, Utset T, Alexander K, Wagner-Weiner L. Safety and immunogenicity of the quadrivalent HPV vaccine in female Systemic Lupus Erythematosus patients aged 12 to 26 years. Pediatr Rheumatol Online J. 2013;11:29.

# **Excluded references by full text**

**Supplement Table S7: References excluded by full-text**

| **Exclusion reason** | **References** |
| --- | --- |
| **Abstract (n=28)** | 1. Dhar JP, Essenmacher L, Dhar R, Magee A, Ager J, Sokol R: Safety and immunogenicity of quadrivalent human papilloma virus (qHPV) vaccine (gardasil) in systemic lupus erythematous (SLE), phase I trial completion. Arthritis and Rheumatology 2015; 67. 2. Dhar JP, Essenmacher L, Dhar R, et al.: Safety of gardasil vaccine in systemic lupus erythematosus. Arthritis and Rheumatism 2013; 65: S1214-S5. 3. Dhar JP, Essenmacher L, Dhar R, et al.: Safety of Gardasil vaccine in systemic lupus erythematosu, trial update. Arthritis and Rheumatology 2014; 66: S313. 4. Dhar JP, Essenmacher L, Dhar R, Ragina N, Sokol R: Lack of uptake of prophylactic human papilloma virus (HPV) vaccination among women with SLE in saginaw valley, a high risk population. Arthritis and Rheumatology 2017; 69. 5. Hoecker B, Aguilar M, Schnitzler P, et al.: Vaccination status and titres before and after pediatric renal transplantation: an analysis of the certain registry. Pediatric transplantation 2017; 21: 17-8.      1. Moudgil A, Whyte T, Eid L, et al.: Immunogenicity of quadrivalent human papillomavirus vaccine in adolescent transplant recipients. Pediatric Transplantation 2013; 17: 44. 2. Nailescu C, Nelson RD, Twombley K, et al.: The response to human papillomavirus vaccination in pediatric patients before and after kidney transplantation. American Journal of Transplantation 2019; 19: 976. 3. Nailescu C, Slaven J, Saha C, Shew M: The response to human papillomavirus vaccination in pediatric patients before and after kidney transplantation. Pediatric Transplantation 2015; 19: 80. 4. Nelson D, Neu A, Abraham A, Amaral S, Batisky D, Fadrowski J: Immunogenicity of human papillomavirus recombinant vaccine in children with CKD. Pediatric Nephrology 2016; 31: 1747-8. 5. Papastamelos C, Dokus K, Laryea M: Frequency of Cervical Cancer Screening in Solid Organ Transplant Recipients. American Journal of Transplantation 2022; 22: 612. 6. Praditpornsilpa K, Susantitaphong P, Eiam-Ong S: Immunogenicity and Safety of Quadrivalent Human Papillomavirus (HPV) Types 6/11/16/18 Recombinant Vaccine in CKD Stage IV-V-VD. Journal of the American Society of Nephrology 2015; 26: 242A-3A. 7. Singer N, Wagner-Weiner L, Nanda K, Robinson A, Spalding S, Bukulmez H: Immunization with quadrivalent HPV vaccine (GARDASIL®) appears safe and induces antibody response in JIA: An interim analysis. Annals of the Rheumatic Diseases 2014; 73. 8. Singer NG, Wallette M, Tomanova-Soltys I, Montealegre-Sanchez G: Interim safety data of gardasil in a trial in females with JIA and seronegative arthritis. Arthritis and Rheumatism 2009; 60: 226. 9. Sousa Morais J, Oliveira DG, Faria R, et al.: Human papilloma virus (HPV) vaccination safety in systemic lupus erythematosus cohort-portuguese university hospital single-center cohort study. Annals of the Rheumatic Diseases 2020; 79: 1503-4.      1. Soybilgic A, Holmes L, Onel KB, Utset T, Alexander K, Weiner LW: Safety and immunogenicity of the Quadrivalent HPV vaccine Gardasil in female systemic lupus erythematosus patients aged 9 to 26 years and its effects on the autoantibody profile. Lupus 2010; 19: 177. 2. Soybilgic A, Onel K, Utset TO, Alexander KA, Wagner-Weiner L: Immunogenicity of the quadrivalent recombinant HPV vaccine in female systemic lupus erythematosus patients aged 9 to 26 years. Arthritis and Rheumatism 2010; 62: 464. 3. Stratton P, Battiwalla M, Abdelazim S, et al.: Immunogenicity of HPV quadrivalent vaccine in women after allogeneic HCT is comparable to healthy volunteers. Biology of Blood and Marrow Transplantation 2018; 24: S85-S6. 4. Targhetta C, Simula MP, Depau C, et al.: Safety of vaccines in a cohort of allogeneic stem cell transplant recipients. Haematologica 2019; 104: 132. 5. Mok CC, Chan PT, Ho LY, Yu KL, To CH: Safety of a quadrivalent human papillomavirus (HPV) vaccine in patients with systemic lupus erythematosus. Arthritis and Rheumatism 2011; 63. 6. Mok CC, Ho LY, Fong LS, To CH: Long-term immunogenicity of a quadrivalent human papillomavirus vaccine in patients with systemic lupus erythematosus. Arthritis and Rheumatology 2017; 69. 7. Heijstek MW, Groot N, Scherpenisse M, et al.: Safety and immunogenicity of human papillomavirus vaccination in juvenile patients with rheumatic diseases. Pediatric Rheumatology 2011; 9. 8. Lu Y, Ashworth LA, Bousvaros A, Carey R, Renna HD, Jacobson DL: Immune response to human papillomavirus vaccine (Gardasil) in girls and young women with inflammatory bowel disease. Gastroenterology 2011; 140: S158-S9. 9. Nelson D, Neu A, Abraham A, Amaral S, Batisky D, Fadrowski J: Immunogenicity of human papillomavirus recombinant vaccine (Gardasil) in children with chronic kidney disease. Blood Purification 2016; 41: 230-2. 10. Rosillon D, Willame C, Pladevall M, et al.: Design and feasibility of a study using the clinical practice research datalink general practice online database (CPRD gold) to assess the risk of new onset of auto-immune diseases (NOAD) following administration of the human papillomavirus (HPV)-16/18 AS. Pharmacoepidemiology and Drug Safety 2014; 23: 164-5. 11. Lepage AK, McIntyre RC, Kennedy SE, Pussell BA, Mackie FE: Safety and reactogenicity of the human papilloma virus vaccine in kidney transplant patients. Immunology and Cell Biology 2011; 89: A22. 12. Targhetta C, Simula MP, Depau C, et al.: Safety of vaccines in a cohort of allogeneic stem cell transplantation recipients. Bone Marrow Transplantation 2019; 54: 260. 13. Arnheim-Dahlstrom L, Pasternak B, Svanstrom H, Sparen P, Hviid A: Autoimmune, neurologic, and venous thromboembolic adverse events following administration of a quadrivalent HPV vaccine to adolescent girls in Denmark and Sweden. Pharmacoepidemiology and Drug Safety 2013; 22: 445. 14. Saah A: An evaluation of the long-term effectiveness, immunogenicity, and safety of gardasil in previously vaccinated women. Sexually Transmitted Infections 2011; 87: A357-A8. |
| **Registry entry with corresponding publication report  (n=18)** | 1. Alberta Uo: Safety and Immunogenicity of Human Papillomavirus (HPV) Vaccine in Solid Organ Transplant Recipients. https://classic.clinicaltrials.gov/show/NCT00677677 2008. 2. Chicago Uo, Sharp M, Llc D: Immunogenicity and Safety of HPV Vaccine Gardasil in Young Women. https://classic.clinicaltrials.gov/show/NCT00786409 2008.      1. Children THfS, Foundation TPSI, Sharp M, Llc D: Immunogenicity and Safety of Human Papilloma Virus Vaccine in Solid Organ Transplant Recipients. https://classic.clinicaltrials.gov/show/NCT02624349 2013. 2. Hospital TM: Immunogenicity and Safety of a Quadrivalent Human Papillomavirus (HPV) Vaccine in Patients With SLE: a Controlled Study. https://classic.clinicaltrials.gov/show/NCT00911521 2009. 3. Hospital TM: Long-term Immunogenicity of a HPV Vaccine in SLE. https://classic.clinicaltrials.gov/show/NCT02477254 2015. 4. Institute MHR, Sharp M, Llc D: Does the HPV Vaccine Cause the Same Response in Adolescent Kidney and Liver Transplant Patients as in Healthy Controls? : https://classic.clinicaltrials.gov/show/NCT01101750 2010.      1. Nct: Immune Response After Human Papillomavirus Vaccination in Patients With Autoimmune Disease. https://clinicaltrialsgov/show/NCT00815282 2008. 2. Nct: Long-term Immunogenicity of a HPV Vaccine in SLE. https://clinicaltrialsgov/show/NCT02477254 2015. 3. University JH, Sharp M, Llc D: Antibody Response to Human Papillomavirus Recombinant Vaccine (Gardasil®) in Girls and Young Women With Chronic Kidney Disease. https://classic.clinicaltrials.gov/show/NCT00806676 2008. 4. Universitaire Ziekenhuizen KU Leuven: Study of Safety, Tolerability and Immunogenicity of Gardasil®9 in Immunocompromised Patients. https://clinicaltrials.gov/study/NCT03525210?cond=NCT03525210 2018. 5. Wales TUoNS, Network SCsH, Women's, Children's Hospital A: Immunogenicity of HPV Vaccine in Immunosuppressed Children. https://classic.clinicaltrials.gov/show/NCT02263703 2007. 6. Wulffraat NM, Health NIfP, Environment t, Utrecht UMC: Immune Response After Human Papillomavirus Vaccination in Patients With Autoimmune Disease. https://classic.clinicaltrials.gov/show/NCT00815282 2009. 7. University I, Sharp M, Llc D, Foundation NK: The Impact of the Human Papilloma Virus in Pediatric Chronic Kidney Disease, Dialysis, and Transplant Patients. https://classic.clinicaltrials.gov/show/NCT00767897 2011. 8. University of Alabama at Birmingham: Quadrivalent Human Papillomavirus (qHPV) Vaccine in Cancer Survivors: Cross Sectional Survey and Phase II Open-Label Vaccine Trial. https://clinicaltrials.gov/study/NCT01492582 2012. 9. Wayne State University: Phase I, Safety and Immunogenicity of Gardasil® in Systemic Lupus Erythematosus. https://www.clinicaltrials.gov/study/NCT01741012 2013. 10. [Unknown authors] Immunogenicity and Safety of the Quadrivalent HPV Vaccine Gardasil in Female Systemic Lupus Erythematosus Patients Aged 9-26 11. Boston Children's Hospital: Pilot Study of Immunogenicity and Tolerability to the Quadrivalent Human Papillomavirus Virus-like Particle (VLP) Vaccine (Gardasil) Among IBD Patients on Immunosuppressive Therapy Compared to Healthy Children and Youth Adult Females. https://clinicaltrials.gov/study/NCT00727636 2008. 12. National Heart, Lung, and Blood Institute (NHLBI): A Phase I Trial of Safety and Immunogenicity of Gardasil Vaccination Post Stem Cell Transplantation in Patients With and Without Immunosuppression. <https://clinicaltrials.gov/study/NCT01092195> 2010. |
| **Wrong intervention/exposure (n=10)** | 1. Atiase Y, Effah K, Mawusi Wormenor C, et al.: Prevalence of high-risk human papillomavirus infection among women with diabetes mellitus in Accra, Ghana. BMC women's health 2024; 24: 260. 2. Gernert M, Kiesel M, Frohlich M, et al.: High Prevalence of Genital Human Papillomavirus Infection in Patients With Primary Immunodeficiencies. Frontiers in Immunology 2021; 12: 789345.      1. Garcia M, McGillicuddy C, Rodriguez EM, et al.: Human papillomavirus vaccination uptake among childhood cancer survivors in Western New York. Pediatric blood & cancer 2022; 69: e29962. 2. Reinholdt K, Thomsen LT, Dehlendorff C, et al.: Human papillomavirus-related anogenital premalignancies and cancer in renal transplant recipients: a Danish nationwide, registry-based cohort study. International journal of cancer 2020; 146: 2413-22. 3. Reinholdt K, Larsen HK, Thomsen LT, et al.: Human papillomavirus (HPV)-related anogenital premalignancies and cancer among renal transplant recipients: A Danish nationwide, registry-based cohort study. Transplant International 2019; 32: 228. 4. Foster E, Malloy MJ, Jokubaitis VG, et al.: Increased risk of cervical dysplasia in females with autoimmune conditions-Results from an Australia database linkage study. PloS one 2020; 15: e0234813. 5. Larsen HK, Kjaer SK, Haedersdal M, et al.: Anal Human Papillomavirus Infection in Kidney Transplant Recipients Compared With Immunocompetent Controls. Clinical infectious diseases: an official publication of the Infectious Diseases Society of America 2022; 75: 1993-9. 6. Khorsandi N, Samghabadi P, Gasper C. Immunocompetency Status and Its Association With Multicentric Lower Anogenital Tract Dysplasia. J Low Genit Tract Dis. 2025 Oct 13. 7. Ring LL, Larsen HK, Frederiksen K, et al. Incidence and clearance of cervical and anal high-risk human papillomavirus in kidney transplant recipients: Results from a Danish prospective clinical study. Am J Transplant. 2024 Jul;24(7):1295-1302. 8. Saco A, Carbonell S, Rakislova N, et al. Human Papillomavirus Infection and Cytological Atypia in Female Allogeneic Hematopoietic Stem Cell Transplantation Recipients. Transplantation. 2025 Jul 1;109(7):e371-e378. |
| **Wrong outcomes  (n=5)** | 1. Dhar JP, Essenmacher L, Dhar R, Ragina N, Sokol RJ: Lack of Uptake of Prophylactic Human Papilloma Virus Vaccine Among Women With Systemic Lupus Erythematosus Seen at a Regional Medical Center. Journal of clinical rheumatology: practical reports on rheumatic & musculoskeletal diseases 2019; 25: 348-50. 2. Kaddas HK, Ramsay JM, Ou JY, Fair D, Kepka D, Kirchhoff AC: HPV Vaccination Initiation and Completion Among Pediatric, Adolescent, and Young Adult Cancer Survivors and a Comparison Population Sample Receiving Primary Care. Journal of pediatric hematology/oncology 2023; 45: e236-e43. 3. Bossart S, Daneluzzi C, Moor MB, et al.: HPV Vaccination in immunosuppressed patients with established skin warts and nonmelanoma skin cancer: A single-institutional cohort study. medRxiv 2023. 4. St. Jude Children's Research Hospital: Human Papillomavirus (HPV) Vaccination Among Survivors of Childhood Cancer. <https://clinicaltrials.gov/study/NCT01117389> 2010. 5. Han Y, Cao Q, Hudson MM, Cai J. Seroreversion of Human Papilloma Virus Antibodies in a Childhood Solid Tumor Survivor Following Three-Dose Vaccination. Pediatr Blood Cancer. 2025 Dec;72(12):e32095. |
| **Wrong population  (n=181)** | 1. Arana JE, Harrington T, Cano M, et al.: Post-licensure safety monitoring of quadrivalent human papillomavirus vaccine in the Vaccine Adverse Event Reporting System (VAERS), 2009-2015. Vaccine 2018; 36: 1781-8. 2. Brown J, Baisley K, Kavishe B, et al.: Impact of malaria and helminth infections on immunogenicity of the human papillomavirus-16/18 AS04-adjuvanted vaccine in Tanzania. Vaccine 2014; 32: 611-7. 3. Crawford NW, Hodgson K, Gold M, Buttery J, Wood N: Adverse events following HPV immunization in Australia: Establishment of a clinical network. Human Vaccines and Immunotherapeutics 2016; 12: 2662-5. 4. Harris T, Williams DM, Fediurek J, Scott T, Deeks SL: Adverse events following immunization in Ontario's female school-based HPV program. Vaccine 2014; 32: 1061-6. 5. Iversen OE, Miranda MJ, Ulied A, et al.: Immunogenicity of the 9-valent HPV Vaccine Using 2-Dose Regimens in Girls and Boys Vs A 3-Dose Regimen in Women. JAMA - Journal of the American Medical Association 2016; 316: 2411-21. 6. Jena AB, Goldman DP, Seabury SA: Incidence of sexually transmitted infections after human papillomavirus vaccination among adolescent females. JAMA Internal Medicine 2015; 175: 617-23. 7. London IC: Nonavalent Prophylactic HPV Vaccine (GARDASIL9) After Local Conservative The NOVEL Trial. https://classic.clinicaltrials.gov/show/NCT03979014 2019. 8. Mauro AB, Fernandes EG, Miyaji KT, et al.: Adverse events following Quadrivalent HPV vaccination reported in Sao Paulo State, Brazil, in the first three years after introducing the vaccine for routine immunization (March 2014 to December 2016). Revista do Instituto de Medicina Tropical de Sao Paulo 2019; 61: e43.      1. Meites E, Gorbach PM, Gratzer B, et al.: Monitoring for Human Papillomavirus Vaccine Impact Among Gay, Bisexual, and Other Men Who Have Sex With Men-United States, 2012-2014. The Journal of infectious diseases 2016; 214: 689-96. 2. Noda A, Sakai T, Tsuchiya M, Oyanagi G, Obara T, Mano N: Characteristics of adverse events following immunization reporting in children: The japanese adverse drug event report database. Vaccines 2020; 8: 1-13. 3. Perez G, Lazcano-Ponce E, Hernandez-Avila M, et al.: Safety, immunogenicity, and efficacy of quadrivalent human papillomavirus (types 6, 11, 16, 18) L1 virus-like-particle vaccine in Latin American women. International Journal of Cancer 2008; 122: 1311-8. 4. Ruiz-Sternberg AM, Moreira ED, Jr., Restrepo JA, et al.: Efficacy, immunogenicity, and safety of a 9-valent human papillomavirus vaccine in Latin American girls, boys, and young women. Papillomavirus research (Amsterdam, Netherlands) 2018; 5: 63-74. 5. Siegrist CA, Lewis EM, Eskola J, Evans SJW, Black SB: Human papilloma virus immunization in adolescent and young adults: A cohort study to illustrate what events might be mistaken for adverse reactions. Pediatric Infectious Disease Journal 2007; 26: 979-84.      1. Sundaram ME, Kieke BA, Hanson KE, et al.: Extended surveillance to assess safety of 9-valent human papillomavirus vaccine. Human Vaccines & Immunotherapeutics 2022; 18: 2159215. 2. Tay EH, Garland S, Tang G, et al.: Clinical trial experience with prophylactic HPV 6/11/16/18 VLP vaccine in young women from the Asia-Pacific region. International Journal of Gynecology and Obstetrics 2008; 102: 275-83. 3. Villa LL, Ault KA, Giuliano AR, et al.: Immunologic responses following administration of a vaccine targeting human papillomavirus Types 6, 11, 16, and 18. Vaccine 2006; 24: 5571-83. 4. Bhatla N, Muwonge R, Malvi SG, et al.: Impact of age at vaccination and cervical HPV infection status on binding and neutralizing antibody titres at 10 years after receiving single or higher doses of quadrivalent HPV vaccine. Human Vaccines and Immunotherapeutics 2023; 19: 2289242.      1. Schwarz TF, Galaj A, Spaczynski M, et al.: Ten-year immune persistence and safety of the HPV-16/18 AS04-adjuvanted vaccine in females vaccinated at 15-55 years of age. Cancer Medicine 2017; 6: 2723-31. 2. Luxembourg A, Kjaer SK, Nygard M, et al.: Design of a long-term follow-up effectiveness, immunogenicity and safety study of women who received the 9-valent human papillomavirus vaccine. Contemporary clinical trials 2017; 52: 54-61. 3. Draper E, Bissett SL, Howell-Jones R, et al.: A Randomized, Observer-Blinded Immunogenicity Trial of Cervarix and Gardasil Human Papillomavirus Vaccines in 12-15 Year Old Girls. PLoS ONE 2013; 8: e61825. 4. Huang T, Liu Y, Li Y, et al.: Evaluation on the persistence of anti-HPV immune responses to the quadrivalent HPV vaccine in Chinese females and males: Up to 3.5years of follow-up. Vaccine 2018; 36: 1368-74. 5. Thiem VD, Quang ND, Tuan NH, et al.: Immunogenicity and safety of a nine-valent human papillomavirus vaccine in Vietnamese males and females (9 to 26 years of age): an open-label, phase 3 trial. Human Vaccines and Immunotherapeutics 2021; 17: 1980-5. 6. Ferris DG, Samakoses R, Block SL, et al.: 4-valent human papillomavirus (4vHPV) vaccine in preadolescents and adolescents after 10 years. Pediatrics 2017; 140: e20163947. 7. Pinto LA, Kemp TJ, Torres BN, et al.: Quadrivalent Human Papillomavirus (HPV) Vaccine Induces HPV-Specific Antibodies in the Oral Cavity: Results From the Mid-Adult Male Vaccine Trial. The Journal of infectious diseases 2016; 214: 1276-83. 8. Lazcano-Ponce E, Carnalla-Cortes M, Barrientos-Gutierrez T, et al.: The effect of a booster dose of HPV tetravalent vaccine after 51 months: implications for extended vaccination schedules. El efecto de un refuerzo de la vacuna tetravalente de VPH despues de 51 meses: implicaciones para un esquema de vacunacion extendido 2018; 60: 666-73. 9. Zimmerman RK, Nowalk MP, Lin CJ, et al.: Randomized trial of an alternate human papillomavirus vaccine administration schedule in college-aged women. Journal of Women's Health 2010; 19: 1441-7. 10. Hu SY, Tsang SH, Chen F, et al.: Association between common vaginal infections and cervical non-human papillomavirus (HPV) 16/18 infection in HPV-vaccinated women. Journal of Infectious Diseases 2021; 223: 445-51. 11. Restrepo J, Herrera T, Samakoses R, et al.: Ten-Year Follow-up of 9-Valent Human Papillomavirus Vaccine: Immunogenicity, Effectiveness, and Safety. Pediatrics 2023; 152: e2022060993. 12. Olsson SE, Restrepo JA, Reina JC, et al.: Long-term immunogenicity, effectiveness, and safety of nine-valent human papillomavirus vaccine in girls and boys 9 to 15 years of age: interim analysis after 8 years of follow-up. Papillomavirus research 2020; 10: 100203. 13. Nakalembe M, Banura C, Namujju PB, Mirembe FM: Immunogenicity to the bivalent HPV-16/18 vaccine among adolescent african students exposed to helminths and malaria. Journal of Infection in Developing Countries 2015; 9: 197-205. 14. Senapati R, Nayak B, Kar SK, Dwibedi B: HPV Genotypes distribution in Indian women with and without cervical carcinoma: Implication for HPV vaccination program in Odisha, Eastern India. BMC Infectious Diseases 2017; 17: 30. 15. Hu Y-M, Guo M, Li C-G, et al.: Immunogenicity noninferiority study of 2 doses and 3 doses of an Escherichia coli-produced HPV bivalent vaccine in girls vs. 3 doses in young women. Science China Life sciences 2020; 63: 582-91. 16. Subelj M, Ucakar V, Kraigher A, Klavs I: Adverse events following school-based vaccination of girls with quadrivalent human papillomavirus vaccine in Slovenia, 2009 to 2013. Eurosurveillance 2016; 21. 17. Nct: Extended Follow-Up of Young Women in Costa Rica Who Received Vaccination Against Human Papillomavirus Types 16 and 18 and Unvaccinated Controls. Https://clinicaltrialsgov/show/nct00867464 2009. 18. Sharp M, Llc D, Permanente K: Observational Surveillance Study to Detect Potential Safety Signals in Patients Who Have Had at Least One Dose of GARDASIL™ (V501-031). https://classic.clinicaltrials.gov/show/NCT01078220 2007. 19. Smahelova J, Hamsikova E, Ludvikova V, et al.: Outcomes After Human Papillomavirus Vaccination in Patients With Recurrent Respiratory Papillomatosis: A Nonrandomized Clinical Trial. JAMA otolaryngology-- head & neck surgery 2022; 148: 654-61. 20. Chirila M, Bolboaca SD: Clinical efficiency of quadrivalent HPV (types 6/11/16/18) vaccine in patients with recurrent respiratory papillomatosis. European Archives of Oto-Rhino-Laryngology 2014; 271: 1135-42. 21. Silverberg MJ, Leyden WA, Lam JO, et al.: Effectiveness of catch-up human papillomavirus vaccination on incident cervical neoplasia in a US health-care setting: a population-based case-control study. The lancet Child & adolescent health 2018; 2: 707-14. 22. Castillo-Cano B, Martin-Perez M, Llorente-Garcia A, Montero-Corominas D, Comas-Cufi M, Martin-Merino E: Assessment of thyroiditis risk associated with HPV vaccination among girls aged 9-18 years: A time-varying cohort study. Vaccine 2022; 40: 4816-26. 23. Martin-Merino E, Castillo-Cano B, Martin-Perez M, Llorente-Garcia A, Montero-Corominas D: Evaluation of the Risk of Inflammatory Bowel Disease after the HPV Vaccination in Primary Care in Spain: A Time-Varying Cohort Analysis of Around 390,000 Girls. Drug Safety 2021; 44: 455-66. 24. Harper DM, Franco EL, Wheeler CM, et al.: Sustained efficacy up to 4.5 years of a bivalent L1 virus-like particle vaccine against human papillomavirus types 16 and 18: follow-up from a randomised control trial. Lancet 2006; 367: 1247-55. 25. Liu XC, Bell CA, Simmonds KA, Svenson LW, Russell ML: Adverse events following HPV vaccination, Alberta 2006-2014. Vaccine 2016; 34: 1800-5. 26. Barzon L, Squarzon L, Masiero S, et al.: Neutralizing and cross-neutralizing antibody titres induced by bivalent and quadrivalent human papillomavirus vaccines in the target population of organized vaccination programmes. Vaccine 2014; 32: 5357-62. 27. Arnheim-Dahlstrom L, Pasternak B, Svanstrom H, Sparen P, Hviid A: Autoimmune, neurological, and venous thromboembolic adverse events after immunisation of adolescent girls with quadrivalent human papillomavirus vaccine in Denmark and Sweden: cohort study. BMJ (Clinical research ed) 2013; 347: f5906. 28. Schwarz TF, Huang LM, Valencia A, et al.: A ten-year study of immunogenicity and safety of the AS04-HPV-16/18 vaccine in adolescent girls aged 10-14 years. Human Vaccines and Immunotherapeutics 2019; 15: 1970-9. 29. Nelson EAS, Lam HS, Choi KC, et al.: A pilot randomized study to assess immunogenicity, reactogenicity, safety and tolerability of two human papillomavirus vaccines administered intramuscularly and intradermally to females aged 18-26 years. Vaccine 2013; 31: 3452-60. 30. Munoz N, Manalastas Jr R, Pitisuttithum P, et al.: Safety, immunogenicity, and efficacy of quadrivalent human papillomavirus (types 6, 11, 16, 18) recombinant vaccine in women aged 24-45 years: a randomised, double-blind trial. The Lancet 2009; 373: 1949-57. 31. Bruce MG, Meites E, Bulkow L, et al.: A prospective cohort study of immunogenicity of quadrivalent human papillomavirus vaccination among Alaska Native Children, Alaska, United States. Vaccine 2020; 38: 6585-91. 32. Martellucci CA, Morettini M, Brotherton JML, et al.: Impact of a Human Papillomavirus Vaccination Program within Organized Cervical Cancer Screening: Cohort Study. Cancer Epidemiology Biomarkers and Prevention 2022; 31: 588-94. 33. Coskuner ER, Ozkan TA, Karakose A, Dillioglugil O, Cevik I: Impact of the quadrivalent HPV vaccine on disease recurrence in men exposed to HPV Infection: a randomized study. Journal of sexual medicine 2014; 11: 2785-91. 34. Joura EA, Giuliano AR, Iversen OE, et al.: A 9-valent HPV vaccine against infection and intraepithelial neoplasia in women. New England Journal of Medicine 2015; 372: 711-23. 35. Jia Y, Zhu C, Du J, et al.: Investigating safety profiles of human papillomavirus vaccine across group differences using VAERS data and MedDRA. PeerJ 2019; 2019: 7490. 36. Davis BM, Blake I, Panicker G, et al.: Immunogenicity of quadrivalent human papillomavirus vaccine among Alaska Native children aged 9-14 years at 5 years after vaccination. Vaccine 2024. 37. Lamontagne DS, Thiem VD, Huong VM, Tang Y, Neuzil KM: Immunogenicity of quadrivalent HPV vaccine among girls 11 to 13 years of age vaccinated using alternative dosing schedules: Results 29 to 32 months after third dose. Journal of Infectious Diseases 2013; 208: 1325-34. 38. Phillips A, Hickie M, Totterdell J, et al.: Adverse events following HPV vaccination: 11 years of surveillance in Australia. Vaccine 2020; 38: 6038-46. 39. Petaja T, Pedersen C, Poder A, et al.: Long-term persistence of systemic and mucosal immune response to HPV-16/18 AS04-adjuvanted vaccine in preteen/adolescent girls and young women. International Journal of Cancer 2011; 129: 2147-57. 40. Wu Q, Qian M, Welby S, et al.: Prospective, multi-center post-marketing surveillance cohort study to monitor the safety of the human papillomavirus-16/18 AS04-adjuvanted vaccine in Chinese girls and women aged 9 to 45 years, 2018-2020. Human Vaccines and Immunotherapeutics 2023; 19: 2283912. 41. Willame C, Rosillon D, Zima J, et al.: Risk of new onset autoimmune disease in 9- to 25-year-old women exposed to human papillomavirus-16/18 AS04-adjuvanted vaccine in the United Kingdom. Human Vaccines and Immunotherapeutics 2016; 12: 2862-71. 42. Gee J, Naleway A, Shui I, et al.: Monitoring the safety of quadrivalent human papillomavirus vaccine: Findings from the Vaccine Safety Datalink. Vaccine 2011; 29: 8279-84. 43. Geier DA, Geier MR: A case-control study of quadrivalent human papillomavirus vaccine-associated autoimmune adverse events. Clinical rheumatology 2015; 34: 1225-31. 44. Hu Y, Pan X, Shen L, et al.: Post-licensure safety monitoring of quadrivalent human papillomavirus vaccine using the national adverse event following immunization surveillance system from Zhejiang province, 2018-2020. Human Vaccines and Immunotherapeutics 2021; 17: 5447-53. 45. LaMontagne DS, Mugisha E, Pan Y, et al.: Immunogenicity of bivalent HPV vaccine among partially vaccinated young adolescent girls in Uganda. Vaccine 2014; 32: 6303-11. 46. Gilca V, Sauvageau C, Boulianne N, et al.: The effect of a booster dose of quadrivalent or bivalent HPV vaccine when administered to girls previously vaccinated with two doses of quadrivalent HPV vaccine. Human Vaccines and Immunotherapeutics 2015; 11: 732-8. 47. Geier DA, Geier MR: Quadrivalent human papillomavirus vaccine and autoimmune adverse events: a case-control assessment of the vaccine adverse event reporting system (VAERS) database. Immunologic research 2017; 65: 46-54. 48. Scheller NM, Svanstrom H, Pasternak B, et al.: Quadrivalent HPV vaccination and risk of multiple sclerosis and other demyelinating diseases of the central nervous system. JAMA 2015; 313: 54-61.      1. Yoon D, Lee JH, Lee H, Shin JY: Association between human papillomavirus vaccination and serious adverse events in South Korean adolescent girls: Nationwide cohort study. The BMJ 2021; 372: m4931.      1. Castellsague X, Muoz N, Pitisuttithum P, et al.: End-of-study safety, immunogenicity, and efficacy of quadrivalent HPV (types 6, 11, 16, 18) recombinant vaccine in adult women 24-45 years of age. British Journal of Cancer 2011; 105: 28-37. 2. Bi D, Apter D, Eriksson T, et al.: Safety of the AS04-adjuvanted human papillomavirus (HPV)-16/18 vaccine in adolescents aged 12-15 years: end-of-study results from a community-randomized study up to 6.5 years. Human Vaccines and Immunotherapeutics 2020; 16: 1392-403.      1. Sultana CS, Khatun S, Ismat SM, Jahan M: Evaluation of Human Papilloma Virus 16/18 Antibody Titer Seven Years after Vaccination with Cervarix among Adolescent Girls of Bangladesh. Bangladesh Journal of Obstetrics and Gynecology 2019; 34: 72-8. 2. Donken R, Schurink-Van't Klooster TM, Schepp RM, et al.: Immune Responses After 2 Versus 3 Doses of HPV Vaccination up to 41/2 Years After Vaccination: An Observational Study Among Dutch Routinely Vaccinated Girls. The Journal of infectious diseases 2017; 215: 359-67. 3. Gilca V, Sauvageau C, Panicker G, De Serres G, Ouakki M, Unger ER: Antibody persistence after a single dose of quadrivalent HPV vaccine and the effect of a dose of nonavalent vaccine given 3-8 years later-an exploratory study. Human Vaccines and Immunotherapeutics 2019; 15: 503-7. 4. Cameron RL, Ahmed S, Pollock KGJ: Adverse event monitoring of the human papillomavirus vaccines in Scotland. Internal medicine journal 2016; 46: 452-7. 5. Kinoshita T, Abe RT, Hineno A, Tsunekawa K, Nakane S, Ikeda SI: Peripheral sympathetic nerve dysfunction in adolescent Japanese girls following immunization with the human papillomavirus vaccine. Internal Medicine 2014; 53: 2185-200. 6. Maldonado I, Rodriguez Nino N, Valencia CF, et al.: Evaluation of the safety profile of the quadrivalent vaccine against human papillomavirus in the risk of developing autoimmune, neurological, and hematological diseases in adolescent women in Colombia. Vaccine 2024; 42: 2414-20. 7. Maldonado I, Plata M, Gonzalez M, et al.: Effectiveness, immunogenicity, and safety of the quadrivalent HPV vaccine in women and men aged 27-45 years. Human Vaccines and Immunotherapeutics 2022; 18: 2078626. 8. Suragh TA, Lewis P, Arana J, et al.: Safety of bivalent human papillomavirus vaccine in the US vaccine adverse event reporting system (VAERS), 2009-2017. British Journal of Clinical Pharmacology 2018; 84: 2928-32. 9. Skufca J, Ollgren J, Artama M, Ruokokoski E, Nohynek H, Palmu AA: The association of adverse events with bivalent human papilloma virus vaccination: A nationwide register-based cohort study in Finland. Vaccine 2018; 36: 5926-33. 10. Shu Y, Yu Y, Ji Y, et al.: Immunogenicity and safety of two novel human papillomavirus 4- and 9-valent vaccines in Chinese women aged 20-45 years: A randomized, blinded, controlled with Gardasil (type 6/11/16/18), phase III non-inferiority clinical trial. Vaccine 2022; 40: 6947-55.      1. Hernandez-Avila M, Torres-Ibarra L, Stanley M, et al.: Evaluation of the immunogenicity of the quadrivalent HPV vaccine using 2 versus 3 doses at month 21: An epidemiological surveillance mechanism for alternate vaccination schemes. Human Vaccines and Immunotherapeutics 2016; 12: 30-8. 2. Callreus T, Svanstrom H, Nielsen NM, Poulsen S, Valentiner-Branth P, Hviid A: Human papillomavirus immunisation of adolescent girls and anticipated reporting of immune-mediated adverse events. Vaccine 2009; 27: 2954-8. 3. De Carvalho N, Teixeira J, Roteli-Martins CM, et al.: Sustained efficacy and immunogenicity of the HPV-16/18 AS04-adjuvanted vaccine up to 7.3 years in young adult women. Vaccine 2010; 28: 6247-55. 4. Murata S, Takeuchi Y, Yamanaka K, et al.: Safety and immunogenicity of the quadrivalent HPV vaccine in japanese boys: A phase 3, open-label study. Japanese Journal of Infectious Diseases 2019; 72: 299-305. 5. Donken R, Dobson SRM, Marty KD, et al.: Immunogenicity of 2 and 3 Doses of the Quadrivalent Human Papillomavirus Vaccine up to 120 Months Postvaccination: follow-up of a Randomized Clinical Trial. Clinical infectious diseases 2020; 71: 1022-9. 6. Puthanakit T, Huang LM, Chiu CH, et al.: Randomized open trial comparing 2-dose regimens of the human papillomavirus 16/18 as04-adjuvanted vaccine in girls aged 9-14 years versus a 3-dose regimen in women aged 15-25 years. Journal of Infectious Diseases 2016; 214: 525-36.      1. Naleway AL, Crane B, Smith N, et al.: Absence of venous thromboembolism risk following quadrivalent human papillomavirus vaccination, Vaccine Safety Datalink, 2008-2011. Vaccine 2016; 34: 167-71. 2. Brotherton JML, Gold MS, Kemp AS, McIntyre PB, Burgess MA, Campbell-Lloyd S: Anaphylaxis following quadrivalent human papillomavirus vaccination. CMAJ Canadian Medical Association Journal 2008; 179: 525-33. 3. Sridhar G, Tian F, Forshee R, et al.: Evaluation of optic neuritis following human papillomavirus vaccination. Human Vaccines and Immunotherapeutics 2017; 13: 1705-13. 4. Klein NP, Hansen J, Chao C, et al.: Safety of quadrivalent human papillomavirus vaccine administered routinely to females. Archives of Pediatrics and Adolescent Medicine 2012; 166: 1140-8. 5. Kang LW, Crawford N, Tang MLK, et al.: Hypersensitivity reactions to human papillomavirus vaccine in Australian schoolgirls: Retrospective cohort study. BMJ 2008; 337: 1392-6. 6. Liu Z, Zhang L, Yang Y, et al.: Active Surveillance of Adverse Events Following Human Papillomavirus Vaccination: Feasibility Pilot Study Based on the Regional Health Care Information Platform in the City of Ningbo, China. Journal of medical Internet research 2020; 22: e17446. 7. Deceuninck G, Sauvageau C, Gilca V, Boulianne N, De Serres G: Absence of association between Guillain-Barre syndrome hospitalizations and HPV-vaccine. Expert review of vaccines 2018; 17: 99-102. 8. Krogsgaard LW, Helmuth IG, Bech BH, et al.: Are unexplained adverse health events following HPV vaccination associated with infectious mononucleosis? - A Danish nationwide matched case-control study. Vaccine 2020; 38: 5678-84. 9. Martin-Merino E, Castillo-Cano B, Martin-Perez M, Llorente-Garcia A, Montero-Corominas D: Papillomavirus vaccination and Guillain-Barre Syndrome among girls: A cohort study in Spain. Vaccine 2021; 39: 4306-13. 10. Ojha RP, Jackson BE, Tota JE, Offutt-Powell TN, Singh KP, Bae S: Guillain-Barre syndrome following quadrivalent human papillomavirus vaccination among vaccine-eligible individuals in the United States. Human Vaccines and Immunotherapeutics 2014; 10: 232-7. 11. Batmunkh T, Dalmau MT, Munkhsaikhan ME, et al.: A single dose of quadrivalent human papillomavirus (HPV) vaccine is immunogenic and reduces HPV detection rates in young women in Mongolia, six years after vaccination. Vaccine 2020; 38: 4316-24. 12. Bonaldo G, Vaccheri A, D'Annibali O, Motola D: Safety profile of human papilloma virus vaccines: an analysis of the US Vaccine Adverse Event Reporting System from 2007 to 2017. British Journal of Clinical Pharmacology 2019; 85: 634-43. 13. Konno R, Yoshikawa H, Okutani M, et al.: Efficacy of the human papillomavirus (HPV)-16/18 AS04-adjuvanted vaccine against cervical intraepithelial neoplasia and cervical infection in young Japanese women: Open follow-up of a randomized clinical trial up to 4 years post-vaccination. Human Vaccines and Immunotherapeutics 2014; 10: 1781-94. 14. Souayah N, Michas-Martin PA, Nasar A, et al.: Guillain-Barre syndrome after Gardasil vaccination: data from Vaccine Adverse Event Reporting System 2006-2009. Vaccine 2011; 29: 886-9. 15. Satanova A, Bolatbekova R, Kukubassov Y, Ossikbayeva S, Kaidarova D: Vaccination Effectiveness against Human Papillomavirus in Kazakhstan. Asian Pacific journal of cancer prevention: APJCP 2024; 25: 681-8. 16. Meng R, Ma R, Wang J, et al.: Post-marketing surveillance for the safety of the 9-valent human papillomavirus vaccine: a retrospective real-world study in China. Expert review of vaccines 2023; 22: 696-703. 17. Hoes J, Pasmans H, Knol MJ, et al.: Persisting antibody response 9 years after bivalent human papillomavirus (HPV) vaccination in a cohort of Dutch women: Immune response and the relation to genital HPV infections. Journal of Infectious Diseases 2020; 221: 1884-94. 18. Cuschieri K, Kavanagh K, Moore C, Bhatia R, Love J, Pollock KG: Impact of partial bivalent HPV vaccination on vaccine-type infection: a population-based analysis. British journal of cancer 2016; 114: 1261-4. 19. Kjaer SK, Nygard M, Sundstrom K, et al.: Long-term effectiveness of the nine-valent human papillomavirus vaccine in Scandinavian women: interim analysis after 8 years of follow-up. Human Vaccines and Immunotherapeutics 2021; 17: 943-9. 20. Guevara A, Cabello R, Woelber L, et al.: Antibody persistence and evidence of immune memory at 5 years following administration of the 9-valent HPV vaccine. Vaccine 2017; 35: 5050-7. 21. Yih WK, Greene SK, Zichittella L, et al.: Evaluation of the risk of venous thromboembolism after quadrivalent human papillomavirus vaccination among US females. Vaccine 2016; 34: 172-8. 22. Lehtinen M, Eriksson T, Apter D, et al.: Safety of the human papillomavirus (HPV)-16/18 AS04-adjuvanted vaccine in adolescents aged 12-15 years: interim analysis of a large community-randomized controlled trial. Human Vaccines and Immunotherapeutics 2016; 12: 3177-85. 23. Luxembourg A, Brown D, Bouchard C, et al.: Phase II studies to select the formulation of a multivalent HPV L1 virus-like particle (VLP) vaccine. Human Vaccines and Immunotherapeutics 2015; 11: 1313-22. 24. Lehtinen M, Apter D, Eriksson T, et al.: Effectiveness of various human papillomavirus vaccination strategies: A community randomized trial in Finland. Cancer Medicine 2021; 10: 7759-71. 25. Frisch M, Besson A, Clemmensen KKB, Valentiner-Branth P, Molbak K, Hviid A: Quadrivalent human papillomavirus vaccination in boys and risk of autoimmune diseases, neurological diseases and venous thromboembolism. International journal of epidemiology 2018; 47: 634-41. 26. Schwarz TF, Spaczynski M, Schneider A, et al.: Immunogenicity and tolerability of an HPV-16/18 AS04-adjuvanted prophylactic cervical cancer vaccine in women aged 15-55 years. Vaccine 2009; 27: 581-7. 27. Hviid A, Svanstrom H, Scheller NM, Gronlund O, Pasternak B, Arnheim-Dahlstrom L: Human papillomavirus vaccination of adult women and risk of autoimmune and neurological diseases. Journal of internal medicine 2018; 283: 154-65. 28. Konno R, Dobbelaere KO, Godeaux OO, Tamura S, Yoshikawa H: Immunogenicity, reactogenicity, and safety of human papillomavirus 16/18 AS04YAdjuvanted vaccine in japanese women. International Journal of Gynecological Cancer 2009; 19: 905-11. 29. Baisley K, Kemp TJ, Mugo NR, et al.: Comparing one dose of HPV vaccine in girls aged 9-14 years in Tanzania (DoRIS) with one dose in young women aged 15-20 years in Kenya (KEN SHE): an immunobridging analysis of randomised controlled trials. The Lancet Global Health 2024; 12: e491-e9. 30. Grimaldi-Bensouda L, Guillemot D, Godeau B, et al.: Autoimmune disorders and quadrivalent human papillomavirus vaccination of young female subjects. Journal of internal medicine 2014; 275: 398-408. 31. Lazcano-Ponce E, Perez G, Cruz-Valdez A, et al.: Impact of a Quadrivalent HPV6/11/16/18 Vaccine in Mexican Women: Public Health Implications for the Region. Archives of Medical Research 2009; 40: 514-24. 32. Nygard M, Saah A, Munk C, et al.: Evaluation of the long-term anti-human papillomavirus 6 (HPV6), 11, 16, and 18 immune responses generated by the quadrivalent HPV vaccine. Clinical and Vaccine Immunology 2015; 22: 943-8. 33. Amend KL, Turnbull B, Zhou L, et al.: Safety of 4-valent human papillomavirus vaccine in males: a large observational post-marketing study. Human Vaccines and Immunotherapeutics 2022; 18: 2073750. 34. Nakalembe M, Banura C, Namujju PB, Mirembe FM: The levels of anti-HPV16/18 and anti-HPV31/33/35/45/52/58 antibodies among AS04-adjuvanted HPV16/18 vaccinated and non-vaccinated Ugandan girls aged 10-16 years. Infectious Agents and Cancer 2014; 9: 29. 35. Block SL, Nolan T, Sattler C, et al.: Comparison of the immunogenicity and reactogenicity of a prophylactic quadrivalent human papillomavirus (types 6, 11, 16, and 18) L1 virus-like particle vaccine in male and female adolescents and young adult women. Pediatrics 2006; 118: 2135-45. 36. Kang S, Kim KH, Kim YT, et al.: Safety and immunogenicity of a vaccine targeting human papillomavirus types 6, 11, 16 and 18: A randomized, placebo-controlled trial in 176 Korean subjects. International Journal of Gynecological Cancer 2008; 18: 1013-9. 37. Huang Z, He J, Su J, et al.: Immunogenicity and safety of the quadrivalent human papillomavirus vaccine in Chinese females aged 9 to 26 years: A phase 3, open-label, immunobridging study. Vaccine 2021; 39: 760-6. 38. Lin L, Macias Parra M, Sierra VY, et al.: Long-term Immunogenicity and Safety of the AS04-adjuvanted Human Papillomavirus-16/18 Vaccine in Four- to Six-year-old Girls: three-year Follow-up of a Randomized Phase III Trial. Pediatric infectious disease journal 2019; 38: 1061-7. 39. Villa LL, Costa RLR, Petta CA, et al.: High sustained efficacy of a prophylactic quadrivalent human papillomavirus types 6/11/16/18 L1 virus-like particle vaccine through 5 years of follow-up. British Journal of Cancer 2006; 95: 1459-66. 40. Luna J, Plata M, Gonzalez M, et al.: Long-term follow-up observation of the safety, immunogenicity, and effectiveness of GardasilTM in adult women. PLoS ONE 2013; 8: e83431.      1. Reisinger KS, Block SL, Lazcano-Ponce E, et al.: Safety and persistent immunogenicity of a quadrivalent human papillomavirus types 6, 11, 16, 18 L1 virus-like particle vaccine in preadolescents and adolescents: A randomized controlled trial. Pediatric Infectious Disease Journal 2007; 26: 201-9. 2. Giuliano AR, Isaacs-Soriano K, Torres BN, et al.: Immunogenicity and safety of Gardasil among mid-adult aged men (27-45 years)-The MAM Study. Vaccine 2015; 33: 5640-6. 3. Schwarz TF, Huang LM, Medina DMR, et al.: Four-year follow-up of the immunogenicity and safety of the HPV-16/18 AS04-adjuvanted vaccine when administered to adolescent girls aged 1014 years. Journal of Adolescent Health 2012; 50: 187-94. 4. Donahue JG, Kieke BA, Lewis EM, et al.: Near real-time surveillance to assess the safety of the 9-valent human papillomavirus vaccine. Pediatrics 2019; 144: e20191808. 5. Iwata S, Murata S, Rong Han S, Wakana A, Sawata M, Tanaka Y: Safety and immunogenicity of a 9-valent human papillomavirus vaccine administered to 9- to 15-year-old Japanese girls. Japanese Journal of Infectious Diseases 2017; 70: 368-73. 6. Ferreira Costa AP, Goncalves AK, Machado PRL, et al.: Immune Response to Human Papillomavirus One Year after Prophylactic Vaccination with AS04-Adjuvanted HPV-16/18 Vaccine: HPV-Specific IgG and IgA Antibodies in the Circulation and the Cervix. Asian Pacific journal of cancer prevention : APJCP 2018; 19: 2313-7. 7. Giuliano AR, Lazcano-Ponce E, Villa L, et al.: Impact of baseline covariates on the immunogenicity of a quadrivalent (types 6, 11, 16, and 18) human papillomavirus virus-like-particle vaccine. Journal of infectious diseases 2007; 196: 1153-62. 8. Van Damme P, Meijer CJLM, Kieninger D, et al.: A phase III clinical study to compare the immunogenicity and safety of the 9-valent and quadrivalent HPV vaccines in men. Vaccine 2016; 34: 4205-12. 9. Huang LM, Puthanakit T, Cheng-Hsun C, et al.: Sustained immunogenicity of 2-dose human papillomavirus 16/18 AS04-adjuvanted vaccine schedules in girls aged 9-14 years: A randomized trial. Journal of Infectious Diseases 2017; 215: 1711-9. 10. Seeger JD, Amend KL, Turnbull BR, et al.: Incident autoimmune conditions among males receiving quadrivalent human papillomavirus vaccine in the United States. Vaccine 2023; 41: 1826-33. 11. Zeng Y, Moscicki A-B, Sahasrabuddhe VV, et al.: A prospective, single-arm, open-label, non-randomized, phase IIa trial of a nonavalent prophylactic HPV vaccine to assess immunogenicity of a prime and deferred-booster dosing schedule among 9-11 year-old girls and boys - clinical protocol. BMC cancer 2019; 19: 290. 12. Porras C, Sampson JN, Herrero R, et al.: Rationale and design of a double-blind randomized non-inferiority clinical trial to evaluate one or two doses of vaccine against human papillomavirus including an epidemiologic survey to estimate vaccine efficacy: The Costa Rica ESCUDDO trial. Vaccine 2022; 40: 76-88. 13. Rivera Medina DM, Valencia A, de Velasquez A, et al.: Safety and Immunogenicity of the HPV-16/18 AS04-Adjuvanted Vaccine: A Randomized, Controlled Trial in Adolescent Girls. Journal of Adolescent Health 2010; 46: 414-21. 14. Giacomet V, Penagini F, Trabattoni D, et al.: Safety and immunogenicity of a quadrivalent human papillomavirus vaccine in HIV-infected and HIV-negative adolescents and young adults. Vaccine 2014; 32: 5657-61. 15. Neuzil KM, Canh DG, Thiem VD, et al.: Immunogenicity and reactogenicity of alternative schedules of HPV vaccine in Vietnam: A cluster randomized noninferiority trial. JAMA 2011; 305: 1424-32. 16. Hillman RJ, Giuliano AR, Palefsky JM, et al.: Immunogenicity of the quadrivalent human papillomavirus (type 6/11/16/18) vaccine in males 16 to 26 years old. Clinical and Vaccine Immunology 2012; 19: 261-7. 17. Garland SM, Cheung TH, McNeill S, et al.: Safety and immunogenicity of a 9-valent HPV vaccine in females 12-26 years of age who previously received the quadrivalent HPV vaccine. Vaccine 2015; 33: 6855-64. 18. Lv H, Wang S, Liang Z, et al.: Immunogenicity and safety of the 9-valent human papillomavirus vaccine in Chinese females 9-45 years of age: A phase 3 open-label study. Vaccine 2022; 40: 3263-71. 19. Joura EA, Ulied A, Vandermeulen C, et al.: Immunogenicity and safety of a nine-valent human papillomavirus vaccine in women 27-45years of age compared to women 16-26years of age: An open-label phase 3 study. Vaccine 2021; 39: 2800-9.      1. Paavonen J, Naud P, Salmeron J, et al.: Efficacy of human papillomavirus (HPV)-16/18 AS04-adjuvanted vaccine against cervical infection and precancer caused by oncogenic HPV types (PATRICIA): final analysis of a double-blind, randomised study in young women. The Lancet 2009; 374: 301-14. 2. Naud PS, Roteli-Martins CM, De Carvalho NS, et al.: Sustained efficacy, immunogenicity, and safety of the HPV-16/18 AS04-adjuvanted vaccine: Final analysis of a long-term follow-up study up to 9.4 years post-vaccination. Human Vaccines and Immunotherapeutics 2014; 10: 2147-62. 3. Grimaldi-Bensouda L, Rossignol M, Kone-Paut I, et al.: Risk of autoimmune diseases and human papilloma virus (HPV) vaccines: Six years of case-referent surveillance. Journal of autoimmunity 2017; 79: 84-90. 4. Vesikari T, Brodszki N, Van Damme P, et al.: A Randomized, Double-Blind, Phase III Study of the Immunogenicity and Safety of a 9-Valent Human Papillomavirus L1 Virus-Like Particle Vaccine (V503) Versus Gardasil in 9-15-Year-Old Girls. Pediatric Infectious Disease Journal 2015; 34: 992-8. 5. Lin CJ, Zimmerman RK, Nowalk MP, Huang HH, Raviotta JM: Randomized controlled trial of two dosing schedules for human papillomavirus vaccination among college age males. Vaccine 2014; 32: 693-9. 6. Dorton BJ, Vitonis AF, Feldman S: Comparing Cervical Cytology and Histology Among Human Papillomavirus-Vaccinated and -Unvaccinated Women in an Academic Colposcopy Clinic. Obstetrics and gynecology 2015; 126: 785-91. 7. Paavonen J, Jenkins D, Bosch FX, et al.: Efficacy of a prophylactic adjuvanted bivalent L1 virus-like-particle vaccine against infection with human papillomavirus types 16 and 18 in young women: an interim analysis of a phase III double-blind, randomised controlled trial. Lancet 2007; 369: 2161-70. 8. Slade BA, Leidel L, Vellozzi C, et al.: Postlicensure safety surveillance for quadrivalent human papillomavirus recombinant vaccine. JAMA 2009; 302: 750-7. 9. Goldstone SE, Giuliano AR, Palefsky JM, et al.: Efficacy, immunogenicity, and safety of a quadrivalent HPV vaccine in men: results of an open-label, long-term extension of a randomised, placebo-controlled, phase 3 trial. The Lancet Infectious diseases 2022; 22: 413-25. 10. Szarewski A, Poppe WA, Skinner SR, et al.: Efficacy of the human papillomavirus (HPV)-16/18 AS04-adjuvanted vaccine in women aged 15-25 years with and without serological evidence of previous exposure to HPV-16/18. International journal of cancer 2012; 131: 106-16.      1. Apter D, Wheeler CM, Paavonen J, et al.: Efficacy of human papillomavirus 16 and 18 (HPV-16/18) AS04-adjuvanted vaccine against cervical infection and precancer in young women: Final event-driven analysis of the randomized, double-blind PATRICIA trial. Clinical and Vaccine Immunology 2015; 22: 361-73. 2. Sankaranarayanan R, Prabhu PR, Pawlita M, et al.: Immunogenicity and HPV infection after one, two, and three doses of quadrivalent HPV vaccine in girls in India: A multicentre prospective cohort study. The Lancet Oncology 2016; 17: 67-77. 3. Gilca V, Sauvageau C, Boulianne N, et al.: Immunogenicity of quadrivalent HPV and combined hepatitis A and B vaccine when co-administered or administered one month apart to 9-10 year-old girls according to 0-6 month schedule. Human Vaccines and Immunotherapeutics 2014; 10: 2438-45. 4. Schwarz TF, Huang LM, Lin TY, et al.: Long-term immunogenicity and safety of the HPV-16/18 AS04-adjuvanted vaccine in 10- to 14-year-old girls: open 6-year follow-up of an initial observer-blinded, randomized trial. Pediatric infectious disease journal 2014; 33: 1255-61. 5. Villa LL, Costa RLR, Petta CA, et al.: Prophylactic quadrivalent human papillomavirus (types 6, 11, 16, and 18) L1 virus-like particle vaccine in young women: A randomised double-blind placebo-controlled multicentre phase II efficacy trial. Lancet Oncology 2005; 6: 271-8. 6. Brown DR, Castellsague X, Ferris D, et al.: Human papillomavirus seroprevalence and seroconversion following baseline detection of nine human papillomavirus types in young women. Tumour Virus Research 2022; 13: 200236. 7. Wheeler CM, Bautista OM, Tomassini JE, Nelson M, Sattler CA, Barr E: Safety and immunogenicity of co-administered quadrivalent human papillomavirus (HPV)-6/11/16/18 L1 virus-like particle (VLP) and hepatitis B (HBV) vaccines. Vaccine 2008; 26: 686-96. 8. Reisinger KS, Block SL, Collins-Ogle M, et al.: Safety, tolerability, and immunogenicity of gardasil given concomitantly with Menactra and Adacel. Pediatrics 2010; 125: 1142-51. 9. Arredondo JL, Villagomez Martinez SM, Concepcion Morales M, et al.: Immunogenicity and safety of a tetravalent dengue vaccine and a bivalent HPV vaccine given concomitantly or sequentially in girls aged 9 to 14 years in Mexico. Vaccine 2021; 39: 3388-96. 10. Moscicki AB, Wheeler CM, Romanowski B, et al.: Immune responses elicited by a fourth dose of the HPV-16/18 AS04-adjuvanted vaccine in previously vaccinated adult women. Vaccine 2012; 31: 234-41. 11. Welby S, Rosillon D, Feng Y, Borys D: Progression from human papillomavirus (HPV) infection to cervical lesion or clearance in women (18-25 years): Natural history study in the control arm subjects of AS04-HPV-16/18 vaccine efficacy study in China between 2008 and 2016. Expert Review of Vaccines 2022; 21: 407-13. 12. Dobson SRM, McNeil S, Dionne M, et al.: Immunogenicity of 2 doses of HPV vaccine in younger adolescents vs 3 doses in young women: A randomized clinical trial. JAMA 2013; 309: 1793-802. 13. Van Damme P, Olsson SE, Block S, et al.: Immunogenicity and safety of a 9-valent HPV vaccine. Pediatrics 2015; 136: e28-e39. 14. Safaeian M, Porras C, Pan Y, et al.: Durable antibody responses following one dose of the bivalent human papillomavirus L1 virus-like particle vaccine in the Costa Rica vaccine trial. Cancer Prevention Research 2013; 6: 1242-50. 15. Anonymous: Sustained efficacy and immunogenicity of the human papillomavirus (HPV)-16/18 AS04-adjuvanted vaccine: analysis of a randomised placebo-controlled trial up to 6.4 years. The Lancet 2009; 374: 1975-85. 16. Mikamo H, Yamagishi Y, Murata S, et al.: Efficacy, safety, and immunogenicity of a quadrivalent HPV vaccine in Japanese men: A randomized, Phase 3, placebo-controlled study. Vaccine 2019; 37: 1651-8. 17. Bornstein J, Roux S, Petersen LK, et al.: Three-year follow-up of 2-dose versus 3-dose HPV vaccine. Pediatrics 2021; 147: e20194035. 18. Goncalves AK, Giraldo PC, Farias KJ, et al.: Characterization of Immunoglobulin A/G Responses During 3 Doses of the Human Papillomavirus-16/18 ASO4-Adjuvanted Vaccine. Sexually transmitted diseases 2016; 43: 335-9. 19. Sankaranarayanan R, Joshi S, Muwonge R, et al.: Can a single dose of human papillomavirus (HPV) vaccine prevent cervical cancer? Early findings from an indian study. Vaccine 2018; 36: 4783-91. 20. Li R, Li Y, Radley D, et al.: Safety and immunogenicity of a vaccine targeting human papillomavirus types 6, 11, 16 and 18: A randomized, double-blind, placebo-controlled trial in Chinese males and females. Vaccine 2012; 30: 4284-91. 21. Roteli-Martins CM, Naud P, De Borba P, et al.: Sustained immunogenicity and efficacy of the HPV-16/18 AS04-adjuvanted vaccine: up to 8.4 years of follow-up. Human vaccines & immunotherapeutics 2012; 8: 390-7. 22. Pedersen C, Petaja T, Strauss G, et al.: Immunization of Early Adolescent Females with Human Papillomavirus Type 16 and 18 L1 Virus-Like Particle Vaccine Containing AS04 Adjuvant. Journal of Adolescent Health 2007; 40: 564-71. 23. Harper DM, Franco EL, Wheeler C, et al.: Efficacy of a bivalent L1 virus-like particle vaccine in prevention of infection with human papillomavirus types 16 and 18 in young women: a randomised controlled trial. Lancet (London, England) 2004; 364: 1757-65. 24. Garland SM, Anagani M, Bhatla N, et al.: Immunogenicity and safety of quadrivalent and 9-valent human papillomavirus vaccines in Indian clinical trial participants. Human Vaccines and Immunotherapeutics 2022; 18: 2105067. 25. Ferris D, Samakoses R, Block SL, et al.: Long-term study of a quadrivalent human papillomavirus vaccine. Pediatrics 2014; 134: e657-e65. 26. National Institute of Hygiene and Epidemiology, Vietnam: A Non-inferiority Study Comparing the Immunogenicity of a Standard or an Extended Three-dose Nonavalent Human Papillomavirus Vaccine Schedule Between High-risk Women Aged 18-26 Years and Age-matched Women in the General Population. <https://clinicaltrials.gov/study/NCT06681636> 2024. 27. Dalla Valle D, Benoni R, Soriolo N, et al. Safety profile assessment of HPV4 and HPV9 vaccines through the passive surveillance system of the Veneto Region (Italy) between 2008 and 2022: A 15-year retrospective observational study. Vaccine X. 2024 Jun 21;19:100511. 28. Liu Q, Liang G, Song Y. Adverse events following 9-valent human papillomavirus vaccine (GARDASIL® 9) reported to the Vaccine Adverse Event Reporting System (VAERS), 2015-2024. Hum Vaccin Immunother. 2025 Dec;21(1):2530831. |
| **Wrong publication type  (n=4)** | 1. Ogilvie G, Sauvageau C, M DI, et al.: Immunogenicity of 2 vs 3 doses of the quadrivalent human papillomavirus vaccine in girls aged 9 to 13 years after 60 months. JAMA - Journal of the American Medical Association 2017; 317: 1687-8. 2. Keam SJ, Harper DM: Human papillomavirus types 16 and 18 vaccine (recombinant, AS04 adjuvanted, adsorbed) [CervarixTM]. Drugs 2008; 68: 359-72. 3. Borja-Hart NL, Benavides S, Christensen C: Human papillomavirus vaccine safety in pediatric patients: an evaluation of the Vaccine Adverse Event Reporting System. The Annals of pharmacotherapy 2009; 43: 356-9. 4. Chandra S, Cooke K, Kamath, et al. M: Human papillomavirus vaccination in solid organ transplant patients. Journal of Lower Genital Tract Disease 2024; 28(3 Supplement): S15 |

# **Risk of Bias assessments (ROBINS-I): comparisons 1-3**

**Supplement Table S8: Risk of Bias (ROBINS-I)**

| **Study** | **Outcomes** | **1. Bias due to confounding** | **2. Bias in selection of participants into the study** | **3. Bias in classification of interventions** | **4. Bias due to deviations from intended intervention** | **5. Bias due to missing data** | **6. Bias in measurement of outcomes** | **7. Bias in selection of the reported result** | **Overall risk of bias** |
| --- | --- | --- | --- | --- | --- | --- | --- | --- | --- |
| **Vaccinated immunocompromised group compared to unvaccinated immunocompromised control group with the same disease or condition (comparison 1)** | | | | | | | | | |
| Silverberg 2020 | CIN2+/CIN3+ | Serious  (Insufficient information on confounding variables for sub-sample of interest; analysis to control differences described; probably not all relevant confounders considered) | Serious  (Selection of participants retrospective; selection of participants into the study was probably related to intervention or outcome (due to case-control design) | Moderate  (Vaccination status prospectively retrieved from electronic health records; unclear time points of vaccinations; participants received probably the same vaccine type and number of doses) | Low  (We cannot exclude deviations from intended intervention. However, no major deviations are expected in the context of this study) | Moderate  (Insufficient information reported on missing data) | Moderate  (Retrospective study; assessment probably appropriate and comparable between groups; unclear if follow-up differed between groups) | Moderate  (No protocol; selection of the reported result unlikely, since the outcome is commonly reported) | Serious |
| **Vaccinated immunocompromised group compared to other vaccinated immunocompromised control group with a different disease or condition that affects the immune system (comparison 2)** | | | | | | | | | |
| Nelson 2016 | Immunogenicity outcomes | Serious  (Confounding variables measured; probably not all relevant confounders considered) | Serious  (Selection of some participants retrospective; selection of participants into the study was probably related to intervention or outcome; not all participants followed from the start of the intervention (i.e. sometimes pre-vaccinated) | Moderate  (Vaccination status for retrospective group probably from medical records (verified by physician); unclear if time points of vaccination differ between participants; vaccine types do not differ between participants; doses do not differ between participants) | Moderate  (We cannot exclude deviations from intended intervention. However, no major deviations are expected in the context of this study; participants with incomplete vaccination were excluded from analyses) | Serious  (Considerable amount of data missing; reasons for missing participant data insufficiently described; proportion of participants between groups different) | Serious  (Assessment appropriate and comparable between groups; follow-up time different between groups) | Moderate  (No protocol; selection of the reported result unlikely, since the outcome is commonly reported) | Serious |
| Nailescu 2020 | Immunogenicity outcomes | Serious  (Confounding variables measured; differences in sex and race; analysis to control differences described; probably not all relevant confounders considered) | Low  (Selection of participants prospective; selection of participants into the study probably not related to intervention or outcome; participants probably followed from the start of the intervention) | Low  (Intervention groups clearly defined; vaccination status prospectively retrieved and probably recorded by study team) | Moderate  (We cannot exclude deviations from intended intervention. However, no major deviations are expected in the context of this study; participants with incomplete vaccination were excluded from analyses) | Moderate  (Insufficient information reported on missing data) | Low  (Unclear blinding of outcome assessments; assessment appropriate and comparable between groups; follow-up time similar between groups) | Moderate  (No protocol; selection of the reported result unlikely, since the outcome is commonly reported) | Serious |
| Nailescu 2020 | Serious adverse events | Serious  (Confounding variables measured; differences in sex and race; analysis to control differences described; probably not all relevant confounders considered) | Low  Selection of participants prospective; selection of participants into the study probably not related to intervention or outcome; participants probably followed from the start of the intervention) | Low  (Intervention groups clearly defined; vaccination status prospectively retrieved and probably recorded by study team) | Moderate  (We cannot exclude deviations from intended intervention. However, no major deviations are expected in the context of this study; participants with incomplete vaccination were excluded from analyses) | Moderate  (Insufficient information reported on missing data) | Serious  (Unclear blinding of outcome assessments; subjective outcome; assessment appropriate and comparable between groups; follow-up time similar between groups) | Moderate  (No protocol; selection of the reported result unlikely, since the outcome is commonly reported) | Serious |
| **Vaccinated immunocompromised group compared to vaccinated healthy control group (comparison 3)** | | | | | | | | | |
| Dhar 2017 | Immunogenicity outcomes | Critical  (Study compares participants to historic control group; considerable confounding expected) | No further assessment due to considerable confounding | No further assessment due to considerable confounding | No further assessment due to considerable confounding | No further assessment due to considerable confounding | No further assessment due to considerable confounding | No further assessment due to considerable confounding | Critical |
| Dhar 2017; Dhar 2018 | Serious adverse events | Critical  (Study compares participants to historic control group; considerable confounding expected) | No further assessment due to considerable confounding | No further assessment due to considerable confounding | No further assessment due to considerable confounding | No further assessment due to considerable confounding | No further assessment due to considerable confounding | No further assessment due to considerable confounding | Critical |
| Esposito 2014 | Immunogenicity outcomes | Serious  (Confounding variables measured; probably not all relevant confounders considered) | Low  (Selection of participants prospective; selection of participants into the study probably not related to intervention or outcome; participants probably followed from the start of the intervention) | Low  (Intervention groups clearly defined; vaccination status prospectively retrieved and probably recorded by study team) | Low  (We cannot exclude deviations from intended intervention. However, no major deviations are expected in the context of this study) | Moderate  (Insufficient information reported on missing data) | Low  (Unclear blinding of outcome assessments; assessment appropriate and comparable between groups; follow-up time similar between groups) | Moderate  (No protocol; selection of the reported result unlikely, since the outcome is commonly reported) | Serious |
| Esposito 2014 | Serious adverse events | Serious  (Confounding variables measured; probably not all relevant confounders considered) | Low  (Selection of participants prospective; selection of participants into the study probably not related to intervention or outcome; participants probably followed from the start of the intervention) | Low  (Intervention groups clearly defined; vaccination status prospectively retrieved and probably recorded by study team) | Low  (We cannot exclude deviations from intended intervention. However, no major deviations are expected in the context of this study) | Moderate  (Insufficient information reported on missing data) | Serious  (Unclear blinding of outcome assessments; subjective measurement; follow-up time probably similar between groups) | Moderate  (No protocol; selection of the reported result unlikely, since the outcome is commonly reported) | Serious |
| Grein 2020a | Immunogenicity outcomes | Serious  (Confounding variables measured; some participants in the intervention group were seropositive at baseline, while we had no information for the control group; not all relevant confounders considered) | Serious  (Selection of some participants retrospective; selection of participants into the study was probably related to intervention or outcome; not all participants followed from the start of the intervention (i.e. sometimes pre-vaccinated) | Serious  (Vaccination status for retrospective group probably self-reported; unclear if time points of vaccination differ between participants; vaccine types do not differ between participants; doses differ between participants) | Low  (We cannot exclude deviations from intended intervention. However, no major deviations are expected in the context of this study) | Serious  (Data missing; reasons for missing participant data insufficiently described; analysis to address missing data probably insufficient) | Low  (Unclear blinding of outcome assessments; assessment appropriate and comparable between groups; follow-up time similar between groups) | Moderate  (No protocol; selection of the reported result unlikely, since the outcome is commonly reported) | Serious |
| Grein 2020a | Serious adverse events | Serious  (Confounding variables measured; some participants in the intervention group were seropositive at baseline, while we had no information for the control group; not all relevant confounders considered) | Serious  (Selection of some participants retrospective; selection of participants into the study was probably related to intervention or outcome; not all participants followed from the start of the intervention (i.e. sometimes pre-vaccinated)) | Serious  (Vaccination status for retrospective group probably self-reported; unclear if time points of vaccination differ between participants; vaccine types do not differ between participants; doses differ between participants) | Low  (We cannot exclude deviations from intended intervention. However, no major deviations are expected in the context of this study) | Serious  (Data missing; reasons for missing participant data insufficiently described; analysis to address missing data probably insufficient) | Serious  (Unclear blinding of outcome assessments; subjective measurement; follow-up time probably similar between groups) | Moderate  (No protocol; selection of the reported result unlikely, since the outcome is commonly reported) | Serious |
| Grein 2020b | Immunogenicity outcomes | Critical  (Confounding variables measured; differences in age and seropositivity at baseline between groups; no analysis to control differences described; probably not all relevant confounders considered) | No further assessment due to considerable confounding | No further assessment due to considerable confounding | No further assessment due to considerable confounding | No further assessment due to considerable confounding | No further assessment due to considerable confounding | No further assessment due to considerable confounding | Critical |
| Grein 2020b | Serious adverse events | Critical  (Confounding variables measured; differences in age and seropositivity at baseline between groups; no analysis to control differences described; probably not all relevant confounders considered) | No further assessment due to considerable confounding | No further assessment due to considerable confounding | No further assessment due to considerable confounding | No further assessment due to considerable confounding | No further assessment due to considerable confounding | No further assessment due to considerable confounding | Critical |
| Heijstek 2014 | Immunogenicity outcomes | Serious  (Confounding variables measured; probably not all relevant confounders considered) | Low  (Selection of participants prospective; selection of participants into the study was probably not related to intervention or outcome; participants probably followed from the start of the intervention) | Low  (Intervention groups clearly defined; vaccination status prospectively retrieved and probably recorded by study team) | Moderate  (We cannot exclude deviations from intended intervention. However, no major deviations are expected in the context of this study; participants with postponed vaccination were excluded from analyses) | Serious  (Data missing; reasons for missing participant data insufficiently described) | Low  (Unclear blinding of outcome assessments; assessment appropriate and comparable between groups; follow-up time similar between groups) | Moderate  (Registry entry (prospective) available; study reports measurement of immunogenicity differently to study registry; however, selection of reported results is unlikely) | Serious |
| Heijstek 2014 | Serious adverse events | Serious  (Confounding variables measured; probably not all relevant confounders considered) | Low  (Selection of participants prospective; selection of participants into the study was probably not related to intervention or outcome; participants probably followed from the start of the intervention) | Low  (Intervention groups clearly defined; vaccination status prospectively retrieved and probably recorded by study team) | Moderate (We cannot exclude deviations from intended intervention. However, no major deviations are expected in the context of this study; participants with postponed vaccination were excluded from analyses) | Serious  (Data missing; reasons for missing participant data insufficiently described) | Serious  (Unclear blinding of outcome assessments; subjective measurement; follow-up time probably similar between groups) | Moderate  (Registry entry (prospective) available; study reports AEs but does not give further information; however, selection of reported results is unlikely) | Serious |
| Jacobson 2013 | Immunogenicity outcomes | Critical  (Study compares participants to historic control group; considerable confounding expected) | No further assessment due to considerable confounding | No further assessment due to considerable confounding | No further assessment due to considerable confounding | No further assessment due to considerable confounding | No further assessment due to considerable confounding | No further assessment due to considerable confounding | Critical |
| Jacobson 2013 | Serious adverse events | Critical  (Study compares participants to historic control group; considerable confounding expected) | No further assessment due to considerable confounding | No further assessment due to considerable confounding | No further assessment due to considerable confounding | No further assessment due to considerable confounding | No further assessment due to considerable confounding | No further assessment due to considerable confounding | Critical |
| Kitano 2023 | Immunogenicity outcomes | Serious  (Confounding variables measured; some participants in the intervention group were seropositive at baseline, while we had no information for the control group; probably not all relevant confounders considered) | Serious  (Selection of some participants retrospective; selection of participants into the study was probably related to intervention or outcome; not all participants followed from the start of the intervention (i.e. sometimes pre-vaccinated) | Moderate  (Vaccination status for retrospective group probably from medical records; unclear if time points of vaccination differ between participants; vaccine types do not differ between participants; doses do not differ between participants) | Moderate  (We cannot exclude deviations from intended intervention. However, no major deviations are expected in the context of this study; participants with incomplete vaccination were excluded from analyses) | Serious  (Data missing; reasons for missing participant data insufficiently described; analysis to address missing data probably insufficient) | Serious  (assessment appropriate and comparable between groups; follow-up time different between groups) | Moderate  (No protocol; selection of the reported result unlikely, since the outcome is commonly reported) | Serious |
| Kitano 2023 | Serious adverse events | Serious  (Confounding variables measured; some participants in the intervention group were seropositive at baseline, while we had no information for the control group; probably not all relevant confounders considered) | Serious  (Selection of some participants retrospective; selection of participants into the study was probably related to intervention or outcome; not all participants followed from the start of the intervention (i.e. sometimes pre-vaccinated) | Moderate  (Vaccination status for retrospective group probably from medical records; unclear if time points of vaccination differ between participants; vaccine types do not differ between participants; doses do not differ between participants) | Moderate  (We cannot exclude deviations from intended intervention. However, no major deviations are expected in the context of this study; participants with incomplete vaccination were excluded from analyses) | Moderate  (Insufficient information reported on missing data) | Serious  (Unclear blinding of outcome assessments; subjective measurement; follow-up time probably similar between groups) | Moderate  (No protocol; selection of the reported result unlikely, since the outcome is commonly reported) | Serious |
| Landier 2022 | Immunogenicity outcomes | Critical  (Study compares participants to historic control group; considerable confounding expected) | No further assessment due to considerable confounding | No further assessment due to considerable confounding | No further assessment due to considerable confounding | No further assessment due to considerable confounding | No further assessment due to considerable confounding | No further assessment due to considerable confounding | Critical |
| Landier 2022 | Serious adverse events | Critical  (Study compares participants to historic control group; considerable confounding expected) | No further assessment due to considerable confounding | No further assessment due to considerable confounding | No further assessment due to considerable confounding | No further assessment due to considerable confounding | No further assessment due to considerable confounding | No further assessment due to considerable confounding | Critical |
| Miyaji 2024 | Immunogenicity outcomes | Critical  (Confounding variables measured; differences in ethnicity, sexual history, contraception, comorbidities and seropositivity at baseline between groups; no analysis to control differences described; probably not all relevant confounders considered) | No further assessment due to considerable confounding | No further assessment due to considerable confounding | No further assessment due to considerable confounding | No further assessment due to considerable confounding | No further assessment due to considerable confounding | No further assessment due to considerable confounding | Critical |
| Miyaji 2024 | Serious adverse events | Critical  (Confounding variables measured; differences in ethnicity, sexual history, contraception, comorbidities and seropositivity at baseline between groups; no analysis to control differences described; probably not all relevant confounders considered) | No further assessment due to considerable confounding | No further assessment due to considerable confounding | No further assessment due to considerable confounding | No further assessment due to considerable confounding | No further assessment due to considerable confounding | No further assessment due to considerable confounding | Critical |
| Mok 2013 | Immunogenicity outcomes | Serious  (Confounding variables measured; probably not all relevant confounders considered) | Low  (Selection of participants prospective; selection of participants into the study probably not related to intervention or outcome; participants probably followed from the start of the intervention) | Low  (Vaccination status prospectively retrieved and probably recorded from study team; time points of vaccination alike between participants; participants received probably the same vaccine type and number of doses) | Low  (We cannot exclude deviations from intended intervention. However, no major deviations are expected in the context of this study) | Serious  (Data missing; reasons for missing participant data insufficiently described; no analysis to address missing data probably insufficient) | Low  (Unclear blinding of outcome assessments; assessment appropriate and comparable between groups; follow-up time similar between groups) | Moderate  (Registry entry (prospective) available; reporting of registry entry insufficient; selection of the reported result unlikely, since the outcome is commonly reported) | Serious |
| Mok 2013 | Serious adverse events | Serious  (Confounding variables measured; probably not all relevant confounders considered) | Low  (Selection of participants prospective; selection of participants into the study probably not related to intervention or outcome; participants probably followed from the start of the intervention) | Low  (Vaccination status prospectively retrieved and probably recorded from study team; time points of vaccination alike between participants; participants received probably the same vaccine type and number of doses) | Low  (We cannot exclude deviations from intended intervention. However, no major deviations are expected in the context of this study) | Moderate  (Insufficient information reported on missing data) | Serious  (Unclear blinding of outcome assessments; subjective measurement; follow-up time probably similar between groups) | Moderate  (Registry entry (prospective) available; reporting of registry entry insufficient; selection of the reported result unlikely, since the outcome is commonly reported) | Serious |
| Nelson 2016 | Serious adverse events | Critical  (Study compares participants to historic control group; considerable confounding expected) | No further assessment due to considerable confounding | No further assessment due to considerable confounding | No further assessment due to considerable confounding | No further assessment due to considerable confounding | No further assessment due to considerable confounding | No further assessment due to considerable confounding | Critical |
| Sauter 2021 | Immunogenicity outcomes | Critical  Insufficient information on confounding variables for sub-sample of interest; study reporting and design indicates risk of considerable confounding | No further assessment due to considerable confounding | No further assessment due to considerable confounding | No further assessment due to considerable confounding | No further assessment due to considerable confounding | No further assessment due to considerable confounding | No further assessment due to considerable confounding | Critical |
| Stratton 2020 | Immunogenicity outcomes | Serious  (Confounding variables measured; differences in sexually activity, education and ethnicity at baseline between groups, but deemed not related to immunogenicity parameters; probably not all relevant confounders considered) | Low  (Selection of participants prospective; selection of participants into the study probably not related to intervention or outcome; participants probably followed from the start of the intervention) | Low  (Vaccination status prospectively retrieved and probably recorded from study team; time points of vaccination alike between participants; participants received probably the same vaccine type and number of doses) | Low  (We cannot exclude deviations from intended intervention. However, no major deviations are expected in the context of this study) | Low  (Data reasonable complete; number of participants with missing outcome data small) | Low  (Unclear blinding of outcome assessments; assessment appropriate and comparable between groups; follow-up time similar between groups) | Moderate  (Protocol (retrospective) and registry entry (prospective) available; study report measurement of immunogenicity differs to study registry; however, selection of reported results is unlikely) | Serious |
| Stratton 2020 | Serious adverse events | Serious  (Confounding variables measured; differences in sexually activity, education and ethnicity at baseline between groups, but deemed not related to immunogenicity parameters; probably not all relevant confounders considered) | Low  (Selection of participants prospective; selection of participants into the study probably not related to intervention or outcome; participants probably followed from the start of the intervention) | Low  (Vaccination status prospectively retrieved and probably recorded from study team; time points of vaccination alike between participants; participants received probably the same vaccine type and number of doses) | Low  (We cannot exclude deviations from intended intervention. However, no major deviations are expected in the context of this study) | Low  (Data reasonable complete; number of participants with missing outcome data small) | Serious  (Unclear blinding of outcome assessments; subjective measurement; follow-up time probably similar between groups) | Moderate  (Protocol (retrospective) and registry entry (prospective) available; reporting of registry entry insufficient; however, selection of reported results is unlikely | Serious |

CIN 2+: cervical intraepithelial neoplasia grade 2+; CIN 3+: cervical intraepithelial neoplasia grade 3+

# **Additional study characteristics: comparisons 1-3**

**Supplement Table S9: Additional study characteristics**

| **Study** | **DoI** | **Clinical condition intervention** | **Clinical condition control** | **Age (years)** | **Sex (%)** | **Immunosuppressive medication at baseline** | **HPV vaccination status at baseline** | **Seropositivity status at baseline** |
| --- | --- | --- | --- | --- | --- | --- | --- | --- |
| **Vaccinated immunocompromised group compared to unvaccinated immunocompromised control group with the same disease or condition (comparison 1)** | | | | | | | | |
| Grönlund 2016 | Interests declared | Autoimmune disease | Autoimmune disease (49 different disease, as further defined in the supplement of Grönlund 2026) | All participants:  10-14: 19,847 15-19: 14,909 20-24: 14,932 25-30: 20,577 | Female (100) | NR | Study excluded participants with a history of HPV vaccination. | NR |
| Silverberg 2020 | No interests declared | Ever prior solid organ transplant, immunosuppressive therapy, HIV-infected^§^ | Ever prior solid organ transplant, immunosuppressive therapy, HIV-infected^*^ | All participants (mean): 26.3 | Female (100) | **Cases:**   - One recent (<18 months) immunosuppressive medication: 470 - Two or more medications: 30   **Controls:**   - One immunosuppressive medication: 2565 - Two or more immunosuppressive medication: 100 | Study only included women eligible for catch-up HPV vaccine since its availability in 2006. | NR |
| **Vaccinated immunocompromised group compared to other vaccinated immunocompromised control group with a different disease or condition that affects the immune system (comparison 2)** | | | | | | | | |
| Nailescu 2020 | No interests declared | Dialysis, transplant recipients (kidney, liver) | Chronic kidney disease (CKD) | All participants (mean, SD): 13.6 (2.6) | Female (100) | Prednisone, Tacrolimus, Mycophenolate mofetil or Azathioprine: 38 (61.3%) | Study excluded participants with a history of HPV vaccination. | All participants were seronegative at baseline for all vaccine HPV types. |
| Nelson 2016 | NR | Dialysis, transplant recipients (kidney) | CKD | CKD (mean, range): 15.2 (11-21),  Dialysis (mean, range): 15.3 (12-18),  Transplant (mean, range): 16.8 (11-21) | Female (100) | **Immunocompromised group (CKD):**   - Prednisone: 1 (4%) - Tacrolimus: 1 (4%) - Rapamycin: 0 (0%) - Cyclosporin: 2 (8%) - Mycophenolate mofetil: 2 (8%) - Abatacept: 0 (0%) - Leflunomide: 0 (0%)   **Immunocompromised group (dialysis):**   - Prednisone: 4 (44%) - Tacrolimus: 2 (22%) - Rapamycin: 1 (11%) - Cyclosporin: 0 (0%) - Mycophenolate mofetil: 1 (11%) - Abatacept: 0 (0%) - Leflunomide: 0 (0%)   **Immunocompromised group (post kidney transplantation):**   - Prednisone: 19 (83%) - Tacrolimus: 17 (74%) - Rapamycin: 3 (13%) - Cyclosporin: 1 (4%) - Mycophenolate mofetil: 15 (65%) - Abatacept: 1 (4%) - Leflunomide: 1 (4%) | Study included those who had been previously vaccinated or started the vaccination series with their primary care physician within 2 years before the enrolment period were eligible for inclusion. | Immunocompromised group: 3/44 (6.8%) for one of the HPV genotypes included in the vaccine |
| **Vaccinated immunocompromised group compared to vaccinated healthy control group (comparison 3)** | | | | | | | | |
| Alter 2014 | NR | Fanconi anaemia (FA) | Healthy participants | Overall (vaccinated) FA: (median, range): 22 (12-59)  DBA: 17 (16-20)  DC: 18 (13-26)  SDS: 22 TAR: NA | FA:  female (≈50)^*^, male (≈50)^*^  DBA:  female (40.5), male: (59.5)  DC:  female (≈30)^*^, male (≈70)^*^  SDS:  female (57.1), male (42.9)  TAR:  female (50), male (50) | NR | NR | NR |
| Dhar 2017 | No interests declared | Systemic lupus erythematosus (SLE) | Healthy participants | SLE: (mean): 38.1 | Female (100) | **Information from inclusion criteria:**   - Prednisone dose <15 mg/day, and hydroxychloroquine dose <400 mg/day | Study excluded participants with a history of HPV vaccination. | 18/34 (52.9%) for HPV 6, 7/34 (20.6%) for HPV 11, 15/34 (44.1%) for HPV 16 and 7/34 (20.6%) for HPV 18. Only seronegative participants at baseline were assessed for immunogenicity outcomes. No information for healthy participants. |
| Esposito 2014 | No interests declared | Juvenile idiopathic arthritis (JIA) | Healthy participants | JIA:  (median, range):15 (12-25);  healthy (median, range): 15 (12-25) | Female (100) | **Immunocompromised group:**   - Non-steroidal anti-inflammatory drugs: 10 (47.6%) - Methotrexate: 5 (23.8%) - Etanercept: 6 (28.6%) | NR | All participants were seronegative at baseline for all vaccine HPV types. |
| Grein 2020a | No interests declared | Childhood SLE | Healthy participants | Childhood SLE: (median, min, max): 11.8 (1-18)  Healthy: (median, min, max): 15.5 (9-19) | Female (100) | **Immunocompromised group:**   - Prednisone: 133 (60.7%) - Azathioprine: 196 (89.5%) - Mycophenolate: 73 (33.3%) - Methotrexate: 16 (7.3%) - Cyclosporine: 13 (5.9%) - Cyclophosphamide: 10 (4.5%) - No medication: 8 (3.6%) | **Immunocompromised group:**   - 5 participants received the first dose of the HPV vaccine before the study (remaining doses within study). - Control group: 2 participants received the first and second dose of the HPV vaccine before the study (remaining doses within study). | **Immunocompromised group:**  37/176 (21%) for HPV 16, 28/176 (16%) for HPV 18  **Control group:**  2/39 (5%) for HPV 16, 1/39 (3%) for HPV 18 |
| Grein 2020b | No interests declared | Juvenile dermatomyositis (JDM) | Healthy participants | JDM: (range): 9-20 | Female (100) | **Immunocompromised group:**   - 35 (74.5%) of the participants used at least one immunosuppressive medication | **Immunocompromised group:**   - 33 participants received the first dose of the HPV vaccine before the study (remaining doses within study). - 18 participants received the first and second dose of the HPV vaccine before the study (remaining doses within study).   **Control group:**   - 2 participants received the first and second dose of the HPV vaccine before the study (remaining doses within study). | **Immunocompromised group:**  10/37 (27%) for HPV 16), 9/37 (24.3%) for HPV 18  **Control group:**  2/39 (5%) for HPV 16, 1/39 (2%) for HPV 18 |
| Gomez- Lobo 2014 | NR | Transplant recipients | Healthy participants | Kidney recipients: (median, range): 14 (11-19) Liver recipients recruited: (median, range): 16 (13-17) | Kidney recipients: female (30%),  male (70%) Liver recipients recruited:  female (100) | **Immunocompromised group (kidney recipients recruited):**   - Tacrolimus: 12 (60%) - Cyclosporine: 1 (5%) - Sirolimus: 1 (5%) - Mycophenolate mofetil: 14 (70%) - Prednisone: 9 (45%)   **Immunocompromised group (liver recipients recruited):**   - Tacrolimus: 3 (60%) - Sirolimus: 1 (20%) - Mycophenolate mofetil: 1 (20%) | NR | All eight liver and kidney transplant recipients with complete vaccination and available results were seronegative at baseline. |
| Heijstek 2014 | Interests declared | JIA | Healthy participants | JIA: (mean, SD):  14.1 (1.6);  Healthy: (mean, SD):14.3 (1.2) | Female (100) | **Immunocompromised group:**   - Methotrexate: 24 (36%) - Non-steroidal anti-inflammatory drugs: 37 (54%) - Other disease modifying antirheumatic drugs: 6 (9%) - Anti-TNFα treatment: 9 (13%) - Anti-IL1 treatment: 1 (1%) - Oral steroids: 0 (0%) | Study excluded participants with a history of HPV vaccination. | **Immunocompromised group:**  2/68 (3%) for HPV 16, 1/68 (1%) for HPV 18  **Control group:**  0/55 (0%) for HPV 16, 1/55 (2%) for HPV 18 |
| Jacobson 2013 | Interests declared | Inflammatory bowel disease (IBD) | Healthy participants | IBD: (median, min, max):  prospectively included 15 (9, 26)  retrospectively included: 18 (14, 26)  Healthy: (range): 9-15 and 15-26 | Female (100) | **Immunocompromised group (prospective intervention group):**   - TNF-alpha inhibitor: 19 (51%) - Immunomodulator: 18 (49%) | The prospective cohort had not previously received HPV immunization. The previously immunized cohort consisted of patients who had already received the 3-dose Gardasil HPV vaccine series. | **Immunocompromised group (prospective intervention group, all age groups):**  2/37 (5.4%) for HPV 6. No information for healthy participants. |
| Kitano 2023 | Interests declared | Transplant recipients | Healthy participants | Kidney: (median,10th-90th percentile): 14 (13.6-16.4) Liver: (median,10th-90th percentile):12.5 (4.8-16.1) Healthy: (median,10th-90th percentile): 16 (14-17.5) | Female (100) | **Immunocompromised group (kidney transplant):**   - One agent: 0 (0%) - Two agents: 1 (14%) - Three agents: 6 (86%) - Sirolimus: 1 (14%) - Tacrolimus: 6 (86%) - Mycophenolate mofetil: 7 (100%) - Steroid: 6 (86%)   **Immunocompromised group (liver transplant):**   - One agent: 9 (90%) - Two agents: 1 (10%) - Three agents: 0 (0%) - Sirolimus: 0 (0%) - Tacrolimus: 10 (100%) - Mycophenolate mofetil: 1 (10%) - Steroid: 0 (0%) | Study excluded participants with incomplete vaccination at baseline. | **Immunocompromised group:**  2/8 (25%) for HPV 6, 11, 16 and 18. No information on healthy participants |
| Landier 2022 | Interests declared | Survivors of cancer§ | Healthy participants | Survivors of cancer: (mean, SD): 15.6 (4.6) | Survivors of cancer:  Female (42)*,  Male (58)* | **Immunocompromised group:**  **Previous treatments:**   - Chemotherapy: 414 (95%) - Radiation: 157 (36%)   **Other:**   - Haematopoietic stem cell transplant: 62 (14%) | Study excluded participants with a history of HPV vaccination. | **Immunocompromised group:**  5/453 (1.1%) for all HPV types. Only seronegative participants at baseline were assessed for immunogenicity outcomes. No information for healthy participants. |
| Miyaji 2024 | Interests declared | Transplant recipients (kidney, kidney + pancreas, liver, lung, heart | Healthy participants | Transplant: (mean, SD): 35.0 (6.8)  Healthy (mean, SD): 32.5 (6.3) | Female (100) | **Immunocompromised group:**   - Immunosuppressive therapy: 125 (100%) - Mycophenolate motefil (MMF), corticosteroids and tacrolimus: 65* (52%) - Azathioprine, corticosteroids and tacrolimus: 14* (11.2%) - MMF, corticosteroids and cyclosporine: 10* (8.0%) | Study excluded participants with a history of HPV vaccination. | **Immunocompromised group:**  19/105 (18.1%) for HPV 6, 27/105 (25.7%) for HPV 11, 28/105 (26.7%) for HPV 16, 16/105 (15.2%) for HPV 18, 9/105 (8.6%) for HPV 31, 11/105 (10.5%) for HPV 33, 24/105 (22.9%) for HPV 52, 17/105 (16.2%) for HPV 58  **Control group:**  28/119 (23.5%) for HPV 6, 11/119 (9.2%) for HPV 11, 32/119 (26.9%) for HPV 16, 24/119 (20.2%) for HPV 18, 13/119 (10.9%) for HPV 31, 12/119 (10.1%) for HPV 33, 23/119 (19.3%) for HPV 52, 15/119 (12.6%) for HPV 58. Only seronegative participants at baseline were assessed for immunogenicity outcomes. |
| Mok 2013 | No interests declared | SLE | Healthy participants | SLE: (mean, SD): 25.8 (3.9) Healthy: (mean, SD): 25.8 (3.9) | Female (100) | **Immunocompromised group:**   - Prednisolone: 35 (70%) - Hydroxychloroquine: 33 (66%) - Azathioprine: 24 (48%) - Mycophenolate mofetil: 9 (18%) - Ciclosporin A: 2 (4%) - Tacrolimus: 5 (10%) - Methotrexate: 3 (6%) | Study excluded participants with a history of HPV vaccination. | Only seronegative participants at baseline were assessed for immunogenicity outcomes. Control group had detectable titres at baseline for HPV 18. |
| Nelson 2016 | NR | CKD, Dialysis, transplant recipients (kidney) | Healthy participants | CKD: (mean, range): 15.2 (11-21),  Dialysis: (mean, range): 15.3 (12-18),  Transplant: (mean, range): 16.8 (11-21) | Female (100) | **Immunocompromised group (CKD):**   - Prednisone: 1 (4%) - Tacrolimus: 1 (4%) - Rapamycin: 0 (0%) - Cyclosporin: 2 (8%) - Mycophenolate mofetil: 2 (8%) - Abatacept: 0 (0%) - Leflunomide: 0 (0%)   **Immunocompromised group (dialysis):**   - Prednisone: 4 (44%) - Tacrolimus: 2 (22%) - Rapamycin: 1 (11%) - Cyclosporin: 0 (0%) - Mycophenolate mofetil: 1 (11%) - Abatacept: 0 (0%) - Leflunomide: 0 (0%)   **Immunocompromised group (post kidney transplantation):**   - Prednisone: 19 (83%) - Tacrolimus: 17 (74%) - Rapamycin: 3 (13%) - Cyclosporin: 1 (4%) - Mycophenolate mofetil: 15 (65%) - Abatacept: 1 (4%) - Leflunomide: 1 (4%) | Study included those who had been vaccinated previously or started the vaccination series with their primary care physician within 2 years before the enrolment period. | **Immunocompromised group:**  3/44 (6.8%) for one of the HPV genotypes included in the vaccine. No information for healthy participants. |
| Sauter 2021 | Interests declared | FA | Healthy participants | FA:  ≤ 11: 93 12-15: 32 16-20: 25 ≥21: 62  Healthy:  ≤ 11: 32 12-15: 25 16-20: 13 ≥21: 41 | Female (55.1)  Male (44.9) | NR | NR | **Immunocompromised group:**  Only seronegative participants at baseline were assessed for immunogenicity outcomes. 48 participants of the overall sample described in the study. |
| Stratton 2020 | Interests declared | Allogeneic hematopoietic stem cell transplant recipients (post-HSCT) | Healthy participants | Receiving immunosuppression medication (median, range): 34.3 (18.3-48.1)   Not receiving immunosuppression medication (median, range): 32.2 (18.3-49.9)  Healthy (median, range): 32.9 (23.0-45.8) | Female (100%) | **Immunocompromised group:**   - Receiving immunosuppression: 23 (52.3%) - Rituximab: 8 (18.2%), 2 (5%) during the study | Study excluded women post-transplant with a history of prior HPV vaccination. | **Immunocompromised group:**  9/44 (20.5%) for HPV 6, 6/44 (13.6%) for HPV 11, 15/44 (34.1%) for HPV16, 18/44 (40.1%) for HPV 18  **Control group:**  2/20 (10%) for HPV6, 3/20 (15%) for HPV 11, 9/20 (45%) for HPV16, 14/20 (40.1%) for HPV 18 |

CKD: chronic kidney disease; DBA: Diamond Blackfan anaemia, DC: dyskeratosis congenital; DoI: declaration of interests; FA: Fanconi anaemia; HIV: human immunodeficiency viruses; HPV: human papillomavirus; IBD: inflammatory bowel disease; JDM: juvenile dermatomyositis; JIA: juvenile idiopathic arthritis; N: number of participants; NR: not reported; NRSI: non-randomised studies of interventions; ; post-HSCT: allogeneic hematopoietic stem cell transplant; SD: standard deviations, SDS: Shwachman Diamond syndrome; SLE: systemic lupus erythematosus; TAR: thrombocytopenia-absent radius; TNF: tumor necrosis factor*Of participants that received at least one dose
^§^ Including: leukaemia, lymphoma, solid tumor

# **Antibody titres: comparison 2**

Supplement Table S10: Comparison 2 - GMT and GMR of HPV 16 at 7 months

| **Study** | **Clinical condition, intervention, measurement unit** | **IG: N** | **IG: GMT** | **IG: dispersion measure GMT** | **CG: N** | **CG: GMT** | **CG: dispersion measure GMT** | **GMR  (95%-CI)** |
| --- | --- | --- | --- | --- | --- | --- | --- | --- |
| Nailescu 2020 | Chronic kidney disease (CKD): CKD vs. dialysis patients; mMU/mL | 18 | Median: 5639.5; Mean: 4390.79 | IQR: 934-9189 | 29 | Median 1581.5; Mean: 1709.62 | IQR: 436-3404 | GMR_Median_: 3.57  GMR_Mean_: 2.57 |
| Nailescu 2020 | Chronic kidney disease (CKD): CKD vs. transplant patients; mMU/mL | 18 | Median 5639.5; Mean: 4390.79 | IQR: 934-9189 | 29 | Median 436; Mean: 508.43 | IQR: 74-4316 | GMR_Median_: 12.94  GMR_Mean_: 8.64 |

CG: control group; CI: confidence interval; CKD: chronic kidney disease; GMR: geometric mean/median ratio; GMT: geometric mean/median titre; IG: intervention group; IQR: inter-quartile range; mMU/mL: milli-Merck Units per millilitre; N: number

**Supplement Table S11: Comparison 2 - GMT and GMR of HPV 18 at 7 months**

| **Study** | **Clinical condition, intervention, measurement unit** | **IG: N** | **IG: GMT** | **IG: dispersion measure GMT** | **CG: N** | **CG: GMT** | **CG: dispersion measure GMT** | **GMR (95%-CI)** |
| --- | --- | --- | --- | --- | --- | --- | --- | --- |
| Nailescu 2020 | Chronic kidney disease (CKD): CKD vs. dialysis patients; mMU/mL | 18 | Median: 1406.5; Mean: 1039.62 | IQR: 150-5121 | 29 | Median 331.5; Mean: 266.45 | IQR: 436-3404 | Median GMR_Median_: 4.24  GMR_Mean_: 3.90 |
| Nailescu 2020 | Chronic kidney disease (CKD): CKD vs. transplant patients; mMU/mL | 18 | Median 1406.5; Mean: 1039.62 | IQR: 150-5121 | 29 | Median 52; Mean: 91.30 | IQR: 74-4316 | GMR_Median_: 27.05  GMR_Mean_: 11.39 |

CG: control group; CI: confidence interval; CKD: chronic kidney disease; GMR: geometric mean/median ratio; GMT: geometric mean/median titre; IG: intervention group; IQR: inter-quartile range; mMU/mL: milli-Merck Units per millilitre; N: number

# **Serious adverse events: comparisons 2-3**

**Supplement Table S12: Serious adverse events**

| **Study** | **Participants, HPV vaccine** | **SAE information** | **Time point, dose** | **IG: Number of SAE** | **IG: total number** | **CG: Number of SAE** | **CG: total number** |
| --- | --- | --- | --- | --- | --- | --- | --- |
| Dhar 2017 (including results from Dhar 2018) | SLE, 4v | All SAEs not related to vaccine or SLE, and all resolved | 4–6 months safety follow-up after the third dose | 9 | 34 | NR | NR |
| Esposito 2014 | JIA, 2v | SAEs, no definition | 14 days after first dose | 0 | 21 | 0 | 21 |
|  |  |  | 14 days after second dose | 0 | 21 | 0 | 21 |
|  |  |  | 14 days after third dose | 0 | 21 | 0 | 21 |
| Grein 2020a | SLE, 4v | SAEs, no definition;  death; not related to vaccination | After first dose within 14 days (at baseline) | 2 | 201 | 0 | 38 |
|  |  | SAEs, no definition;  death; not related to vaccination | After second dose within 14 days (Month 1 or 2) | 2 | 210 | 0 | 38 |
|  |  | SAEs, no definition | After third dose within days (Month 6) | 0 | 180 | 0 | 35 |
| Grein 2020b | JDM, 4v | SAEs following vaccination defined as life-threatening, requires in-patient hospitalization or prolongation of existing hospitalization, results in persistent or significant disability/incapacity, requires intervention to prevent permanent impairment or damage | After first dose within 14 days (at baseline) | 0 | 40 | 0 | 38 |
|  |  |  | After second dose within 14 days (Month 1 or 2) | 0 | 41 | 0 | 38 |
|  |  |  | After third dose within days (Month 6) | 0 | 40 | 0 | 35 |
| Heijstek 2014 | JIA, 4v | SAEs, no definition; majority were preplanned interventions, diagnostic hospital admissions for pre-existing complaints or adverse events associated with the treatment of JIA disease; therefore, all SAEs judged to be unrelated to HPV vaccination | 14 days after each vaccine dose | 11 | 68 | 1 | 55 |
| Jacobson 2013 | IBD, 4v | Admitted to hospital/ went to emergency dept.: Exacerbation of inflammatory bowel disease; unlikely related to the vaccine | Timing relative to dose: Day 3 and 8 | 2 | from 32-35 | NR | NR |
|  |  | Admitted to hospital/ went to emergency dept.: Pneumonia; unlikely related to the vaccine | Timing relative to dose: Week 3 | 1 | from 32-35 | NR | NR |
|  |  | Admitted to hospital/ went to emergency dept.: Endometriosis with an ovarian torsion; unrelated to the vaccine | Timing relative to dose: Day 2 | 1 | from 32-35 | NR | NR |
|  |  | Admitted to hospital/ went to emergency dept.: Sinus pain and ED visit; unrelated to the vaccine | Timing relative to dose: Week 3 | 1 | from 32-35 | NR | NR |
| Kitano 2023 | Transplant, 4v | SAEs defined as adverse events of special interest in previous relevant articles (i.e., autoimmune disease, acute disseminated encephalomyelitis, complex regional pain syndrome, Guillain-Barré syndrome, multiple sclerosis, postural orthostatic tachycardia syndrome and premature ovarian insufficiency) for dose 1-3 | 7 days after vaccination | 0 | 17 | 0 | 19 |
| Landier 2022 | Cancer survivors, 9v | SAEs (all) defined as death, life-threatening conditions, unplanned admission to hospital for longer than 24 h, persistent or substantial disability, second cancer, or other medical event that was deemed by the investigator to jeopardise participant health—were reported in real time until month 24. | 24 months, ≥1 dose | 12 | 182 | NR | NR |
|  |  | From those 12 SAEs: vaccine-related only | 24 months, ≥1 dose | 1 | 182 | NR | NR |
|  |  | From those 12 SAE: death | 24 months, ≥1 dose | 2 | 182 | NR | NR |
|  | Cancer survivors, 4v | Serious AE (all) | 24 months, ≥1 dose | 20 | 253 | NR | NR |
|  |  | From those 20 SAE: vaccine-related only | 24 months, ≥1 dose | 0 | 253 | NR | NR |
|  |  | From those 10 SAEs: death | 24 months, ≥1 dose | 0 | 253 | NR | NR |
| Miyaji 2024 (including results from Moreira dos Santos 2024) | Transplant, 4v | SAE (such as transplanted organ rejection, incl. mortality), | After any dose, full study period | 2 | NR | 0 | NR |
|  |  | SAE after 4 doses, negative after 3 doses | After 4 doses, full study period | 0 | 23 | NA | NA |
|  |  | SAE after 4 doses, positive after 3 doses | After 4 doses, full study period | 0 | 5 | NA | NA |
| Mok 2013 (including results from Mok 2018) | SLE, 4v | SAEs, no definition | Within 12 months, ≥1 dose | 0 | 50 | 0 | 50 |
| Nailescu 2020 | CKD vs. dialysis | SAEs, no definition | Full study period | 0 | 18 | 0 | 18 |
|  | CKD vs. transplant | SAEs, no definition | Full study period | 0 | 18 | 0 | 29 |
| Stratton 2020 | Post-HSCT, on immunosuppressive drugs, 4v | SAE, no definition (same control group as for subgroup below) | For 5 days after each dose vaccination | 0 | 23 | 0 | 20 |
|  | Post-HSCT, not on immunosuppressive drugs, 4v | SAE, no definition (same control group as for subgroup above) | For 5 days after each dose vaccination | 0 | 23 | 0 | 20 |

CKD: chronic kidney disease; CG: control group; HPV: human papilloma virus; IBD: Inflammatory bowel disease; IG: intervention group; JDM: juvenile dermatomyositis; JIA: juvenile idiopathic arthritis; NR: not reported; post-HSCT: allogeneic hematopoietic stem cell transplant; SAE: serious adverse event; SLE: systemic lupus erythematosus; 4v: quadrivalent; 9v: nonavalent

# **Local, systemic and additional adverse events: comparisons 2-3 and single-arm studies**

**Supplement Table S13: Local, systemic and additional adverse events across vaccine types**

| **Population, study** | **AE, dose, time point** | **N IG** | **Total IG** | **% IG** | **N CG** | **Total CG** | **% CG** |
| --- | --- | --- | --- | --- | --- | --- | --- |
| **Bivalent vaccine, local adverse event** | | | | | | | |
| Juvenile idiopathic arthritis,  Esposito 2014;  Heijstek 2014 | Any local event, first dose, 14 days after first dose | 9 | 21 | 42.9 | 10 | 21 | 47.6 |
|  | Any local event, second dose, 14 days after second dose | 9 | 21 | 42.9 | 9 | 21 | 42.9 |
|  | Any local event, third dose, 14 days after third dose | 6 | 21 | 28.6 | 8 | 21 | 38.1 |
|  | Bruise, any dose, 14 days after each vaccine dose | 14 | 54 | 25.9 | 39 | 44 | 88.6 |
|  | Oedema, any dose, 14 days after each vaccine dose | 25 | 54 | 46.3 | 18 | 44 | 40.9 |
|  | Oedema, first dose, 14 days after first dose | 5 | 21 | 23.8 | 8 | 21 | 38.1 |
|  | Oedema, second dose, 14 days after second dose | 6 | 21 | 28.6 | 7 | 21 | 33.3 |
|  | Oedema, third dose, 14 days after third dose | 5 | 21 | 23.8 | 6 | 21 | 28.6 |
|  | Erythema, any dose, 14 days after each vaccine dose | 20 | 54 | 37.0 | 43 | 44 | 97.7 |
|  | Erythema, first dose, 14 days after first dose | 2 | 21 | 9.5 | 4 | 21 | 19.0 |
|  | Erythema, second dose, 14 days after second dose | 3 | 21 | 14.3 | 5 | 21 | 23.8 |
|  | Erythema, third dose, 14 days after third dose | 2 | 21 | 9.5 | 3 | 21 | 14.3 |
|  | Induration, any dose, 14 days after each vaccine dose | 26 | 54 | 48.1 | 21 | 44 | 47.7 |
|  | Pain, any dose, 14 days after each vaccine dose | 52 | 54 | 96.3 | 44 | 44 | 100.0 |
|  | Pain, first dose, 14 days after first dose | 9 | 21 | 42.9 | 7 | 21 | 33.3 |
|  | Pain, second dose, 14 days after second dose | 9 | 21 | 42.9 | 6 | 21 | 28.6 |
|  | Pain, third dose, 14 days after third dose | 4 | 21 | 19.0 | 4 | 21 | 19.0 |
| **Bivalent vaccine, systemic adverse event** | | | | | | | |
| Juvenile idiopathic arthritis,  Esposito 2014;  Heijstek 2014 | At least one systemic event, 14 days after first dose | 3 | 21 | 14.3 | 2 | 21 | 9.5 |
|  | At least one systemic event, 14 days after second dose | 1 | 21 | 4.8 | 1 | 21 | 4.8 |
|  | At least one systemic event, 14 days after third dose | 1 | 21 | 4.8 | 1 | 21 | 4.8 |
|  | Arthralgia (newly onset symptoms or worsening of pre to existing symptoms), 14 days after each vaccine dose | 11 | 54 | 20.4 | 6 | 44 | 13.6 |
|  | Fatigue (newly onset symptoms or worsening of pre to existing symptoms), 14 days after each vaccine dose | 30 | 54 | 55.6 | 22 | 44 | 50.0 |
|  | Fever (>38.5ºC), 14 days after each vaccine dose | 6 | 54 | 11.1 | 3 | 44 | 6.8 |
|  | Fever (≥38°C), 14 days after first dose | 0 | 21 | 0.0 | 0 | 21 | 0.0 |
|  | Fever (≥38°C), 14 days after second dose | 0 | 21 | 0.0 | 0 | 21 | 0.0 |
|  | Fever (≥38°C), 14 days after third dose | 0 | 21 | 0.0 | 0 | 21 | 0.0 |
|  | Headache, 14 days after first dose | 1 | 21 | 4.8 | 0 | 21 | 0.0 |
|  | Headache, 14 days after second dose | 0 | 21 | 0.0 | 0 | 21 | 0.0 |
|  | Headache, 14 days after third dose | 0 | 21 | 0.0 | 0 | 21 | 0.0 |
|  | Headache (newly onset symptoms or worsening of pre to existing symptoms), 14 days after each vaccine dose | 22 | 54 | 40.7 | 22 | 44 | 50.0 |
|  | Malaise, 14 days after first dose | 2 | 21 | 9.5 | 1 | 21 | 4.8 |
|  | Malaise, 14 days after second dose | 1 | 21 | 4.8 | 1 | 21 | 4.8 |
|  | Malaise, 14 days after third dose | 1 | 21 | 4.8 | 1 | 21 | 4.8 |
|  | Myalgia (newly onset symptoms or worsening of pre to existing symptoms), 14 days after each vaccine dose | 29 | 54 | 53.7 | 19 | 44 | 43.2 |
|  | Rash, 14 days after first dose | 0 | 21 | 0.0 | 0 | 21 | 0.0 |
|  | Rash, 14 days after second dose | 0 | 21 | 0.0 | 0 | 21 | 0.0 |
|  | Rash, 14 days after third dose | 0 | 21 | 0.0 | 0 | 21 | 0.0 |
|  | Rash (newly onset symptoms or worsening of pre to existing symptoms), 14 days after each vaccine dose | 11 | 54 | 20.4 | 6 | 44 | 13.6 |
|  | Syncope after vaccination (newly onset symptoms or worsening of pre to existing symptoms), 14 days after each vaccine dose | 1 | 54 | 1.9 | 0 | 44 | 0.0 |
|  | Vomiting/iarrhoea, 14 days after first dose | 1 | 21 | 4.8 | 1 | 21 | 4.8 |
|  | Vomiting/diarrhoea, 14 days after second dose | 0 | 21 | 0.0 | 0 | 21 | 0.0 |
|  | Vomiting/diarrhoea, 14 days after third dose | 0 | 21 | 0.0 | 0 | 21 | 0.0 |
| **Quadrivalent vaccine, local adverse events** | | | | | | | |
| Chronic kidney disease, dialysis and kidney transplant, Nelson 2016 | Bruising, probably any dose, time point NR | 1 | 57 | 1.8 | NA | NA | NA |
|  | Pain, probably any dose, time point NR | 8 | 57 | 14.0 | NA | NA | NA |
| Fanconi anaemia  not on immunosuppression, Stratton 2020 | Oedema: Swelling (1 inch) at injection site, any dose, 5 days after each vaccination (last dose month 6) | 0 | 21 | 0.0 | 4 | 20 | 20.0 |
|  | Oedema: Swelling (2 inches) at injection site, any dose, 5 days after each vaccination (last dose month 6) | 0 | 21 | 0.0 | 0 | 20 | 0.0 |
|  | Oedema: Swelling (3 inches) at injection site, any dose, 5 days after each vaccination (last dose month 6) | 0 | 21 | 0.0 | 0 | 20 | 0.0 |
|  | Oedema: Swelling (>3 inches) at injection site,  any dose, 5 days after each vaccination (last dose month 6) | 0 | 21 | 0.0 | 0 | 20 | 0.0 |
|  | Erythema (mild: 0-1 inch) at injection site, any dose, 5 days after each vaccination (last dose month 6) | 2 | 21 | 9.5 | 5 | 20 | 25.0 |
|  | Erythema (moderate: 2 inches) at injection site, any dose, 5 days after each vaccination (last dose month 6) | 0 | 21 | 0.0 | 0 | 20 | 0.0 |
|  | Erythema (severe: ≥3 inches) at injection site,  any dose, 5 days after each vaccination (last dose month 6) | 0 | 21 | 0.0 | 0 | 20 | 0.0 |
|  | Pruritus (mild) at injection site, any dose, 5 days after each vaccination (last dose month 6) | 0 | 21 | 0.0 | 0 | 20 | 0.0 |
|  | Pruritus (moderate or severe) at injection site, any dose, 5 days after each vaccination (last dose month 6) | 0 | 21 | 0.0 | 0 | 20 | 0.0 |
|  | Other local event (bruise, reaction to bandage adhesive, rash) at injection site, any dose, 5 days after each vaccination (last dose month 6) | 3 | 21 | 14.3 | 2 | 20 | 10.0 |
|  | Pain (mild) at injection site, any dose, 5 days after each vaccination (last dose month 6) | 12 | 21 | 57.1 | 14 | 20 | 70.0 |
|  | Pain (moderate) at injection site, any dose, 5 days after each vaccination (last dose month 6) | 2 | 21 | 9.5 | 6 | 20 | 30.0 |
|  | Pain (severe) at injection site, any dose, 5 days after each vaccination (last dose month 6) | 0 | 21 | 0.0 | 0 | 20 | 0.0 |
| Fanconi anaemia  on immunosuppression, Stratton 2020 | Oedema: Swelling (1 inch) at injection site, any dose, 5 days after each vaccination (last dose month 6) | 4 | 23 | 17.4 | 4 | 20 | 20.0 |
|  | Oedema: Swelling (2 inches) at injection site, any dose, 5 days after each vaccination (last dose month 6) | 1 | 23 | 4.3 | 0 | 20 | 0.0 |
|  | Oedema: Swelling (3 inches) at injection site, any dose, 5 days after each vaccination (last dose month 6) | 1 | 23 | 4.3 | 0 | 20 | 0.0 |
|  | Oedema: Swelling (>3 inches) at injection site, any dose, 5 days after each vaccination (last dose month 6) | 0 | 23 | 0.0 | 0 | 20 | 0.0 |
|  | Erythema (mild: 0-1 inch) at injection site, any dose, 5 days after each vaccination (last dose month 6) | 4 | 23 | 17.4 | 5 | 20 | 25.0 |
|  | Erythema (moderate: 2 inches) at injection site, any dose, 5 days after each vaccination (last dose month 6) | 0 | 23 | 0.0 | 0 | 20 | 0.0 |
|  | Erythema (severe: ≥3 inches) at injection site, any dose, 5 days after each vaccination (last dose month 6) | 2 | 23 | 8.7 | 0 | 20 | 0.0 |
|  | Pruritus (mild) at injection site, any dose, 5 days after each vaccination (last dose month 6) | 3 | 23 | 13.0 | 0 | 20 | 0.0 |
|  | Pruritus (moderate or severe) at injection site, any dose, 5 days after each vaccination (last dose month 6) | 0 | 23 | 0.0 | 0 | 20 | 0.0 |
|  | Other local event (bruise, reaction to bandage adhesive, rash) at injection site, any dose, 5 days after each vaccination (last dose month 6) | 1 | 23 | 4.3 | 2 | 20 | 10.0 |
|  | Pain (mild) at injection site, any dose, 5 days after each vaccination (last dose month 6) | 11 | 23 | 47.8 | 14 | 20 | 70.0 |
|  | Pain (moderate) at injection site, any dose, 5 days after each vaccination (last dose month 6) | 5 | 23 | 21.7 | 6 | 20 | 30.0 |
|  | Pain (severe) at injection site, any dose, 5 days after each vaccination (last dose month 6) | 0 | 23 | 0.0 | 0 | 20 | 0.0 |
| Inflammatory bowel disease, Jacobson 2013 | Oedema, after dose 1 (day 1) | 0 | 35 | 0.0 | NA | NA | NA |
|  | Oedema, after dose 2 (month 2) | 3 | 32 | 9.4 | NA | NA | NA |
|  | Oedema, after dose 3 (month 6) | 2 | 33 | 6.1 | NA | NA | NA |
|  | Erythema, after dose 1 (day 1) | 0 | 35 | 0.0 | NA | NA | NA |
|  | Erythema, after dose 2 (month 2) | 3 | 32 | 9.4 | NA | NA | NA |
|  | Erythema, after dose 3 (month 6) | 6 | 33 | 18.2 | NA | NA | NA |
|  | Itching, after dose 1 (day 1) | 0 | 35 | 0.0 | NA | NA | NA |
|  | Itching, after dose 2 (month 2) | 0 | 32 | 0.0 | NA | NA | NA |
|  | Itching, after dose 3 (month 6) | 2 | 33 | 6.1 | NA | NA | NA |
|  | Pain, after dose 1 (day 1) | 17 | 35 | 48.6 | NA | NA | NA |
|  | Pain, after dose 2 (month 2) | 15 | 32 | 46.9 | NA | NA | NA |
|  | Pain, after dose 3 (month 6) | 17 | 33 | 51.5 | NA | NA | NA |
|  | Swelling and severe pain in arm (minor adverse event), day 3 relative to dose | 1 | NR | NA | NA | NA | NA |
| Juvenile dermatomyositis, Grein 2020b | Bruise, after first dose within 14 days (at baseline) | 0 | 40 | 0.0 | 0 | 38 | 0.0 |
|  | Bruise, after second dose within 14 days (month 1 or 2) | 1 | 41 | 2.4 | 1 | 38 | 2.6 |
|  | Bruise, after third dose within 14 days(month 6) | 0 | 40 | 0.0 | 5 | 35 | 14.3 |
|  | Oedema, after first dose within 14 days (at baseline) | 5 | 40 | 12.5 | 5 | 38 | 13.2 |
|  | Oedema, after second dose within 14 days (month 1 or 2) | 4 | 41 | 9.8 | 8 | 38 | 21.1 |
|  | Oedema, after third dose within 14 days (month 6) | 1 | 40 | 2.5 | 8 | 35 | 22.9 |
|  | Redness, after first dose within 14 days (at baseline) | 5 | 40 | 12.5 | 4 | 38 | 10.5 |
|  | Redness, after second dose within 14 days (month 1 or 2) | 2 | 41 | 4.9 | 3 | 38 | 7.9 |
|  | Redness, after third dose within 14 days (month 6) | 0 | 40 | 0.0 | 2 | 35 | 5.7 |
|  | Induration, after first dose within 14 days (at baseline) | 6 | 40 | 15.0 | 9 | 38 | 23.7 |
|  | Induration, after second dose within 14 days (month 1 or 2) | 4 | 41 | 9.8 | 4 | 38 | 10.5 |
|  | Induration, after third dose within 14 days (month 6) | 4 | 40 | 10.0 | 5 | 35 | 14.3 |
|  | Pain, after first dose within 14 days (at baseline) | 22 | 40 | 55.0 | 23 | 38 | 60.5 |
|  | Pain, after second dose within 14 days (month 1 or 2) | 19 | 41 | 46.3 | 23 | 38 | 60.5 |
|  | Pain, after third dose within 14 days (month 6) | 16 | 40 | 40.0 | 19 | 35 | 54.3 |
| Survivors of cancer, Landier 2022 | Oedema, days 1-5 post any dose (with available safety data) | 15 | 253 | 5.9 | 1722 | 8181 | 21.0 |
|  | Erythema, days 1-5 post any dose (with available safety data) | 15 | 253 | 5.9 | 1774 | 8181 | 21.7 |
|  | Pain, days 1-5 post any dose (with available safety data) | 90 | 253 | 35.6 | 6168 | 8181 | 75.4 |
| Systemic lupus erythematosus,  Dhar 2017;  Mok 2013 | Any AE: Vaccine site reaction, mostly pain, across all time points | NA | NA | 62.0 | NA | NA | 83.90 |
|  | Gastrointestinal (events; none related to vaccine or SLE), across all time points | 49 (events) | 34 | NA | NA | NA | NA |
|  | Erythema and pain at injection site, probably after the vaccine, time point unclear | 3 | 50 | 6.0 | 2 | 50 | 4.0 |
|  | Dermatologic (events; none related to vaccine or SLE) | 45  (events) | 34 | NA | NA | NA | NA |
|  | Rash, probably after the vaccine, time point unclear | 1 | 50 | 2.0 | 0 | 50 | 0.0 |
|  | Rash, during study period | 4 | 27 | 14.8 | NA | NA | NA |
| Systemic lupus erythematosus (children) Grein 2020a | Bruise, after first dose within 14 days (at baseline) | 11 | 179 | 6.1 | 0 | 38 | 0.0 |
|  | Bruise, after second dose within 14 days (month 1 or 2) | 8 | 182 | 4.4 | 1 | 38 | 2.6 |
|  | Bruise, after third dose within 14 days (month 6) | 4 | 194 | 2.1 | 5 | 35 | 14.3 |
|  | Oedema, after first dose within 14 days (at baseline) | 21 | 179 | 11.7 | 5 | 38 | 13.2 |
|  | Oedema, after second dose within 14 days (month 1 or 2) | 30 | 182 | 16.5 | 8 | 38 | 21.1 |
|  | Oedema, after third dose within 14 days (month 6) | 25 | 194 | 12.9 | 8 | 35 | 22.9 |
|  | Redness, after first dose within 14 days (at baseline) | 9 | 179 | 5.0 | 4 | 38 | 10.5 |
|  | Redness, after second dose within 14 days (month 1 or 2) | 14 | 182 | 7.7 | 3 | 38 | 7.9 |
|  | Redness, after third dose within 14 days (month 6) | 9 | 194 | 4.6 | 2 | 35 | 5.7 |
|  | Induration, after first dose within 14 days (at baseline) | 27 | 179 | 15.1 | 9 | 38 | 23.7 |
|  | Induration, after second dose within 14 days (month 1 or 2) | 36 | 182 | 19.8 | 4 | 38 | 10.5 |
|  | Induration, after third dose within 14 days (month 6) | 29 | 194 | 14.9 | 5 | 35 | 14.3 |
|  | Pain, after first dose within 14 days (at baseline) | 109 | 179 | 60.9 | 23 | 38 | 60.5 |
|  | Pain, after second dose within 14 days (month 1 or 2) | 87 | 182 | 47.8 | 23 | 38 | 60.5 |
|  | Pain, after third dose within 14 days (month 6) | 71 | 194 | 36.6 | 19 | 35 | 54.3 |
| Transplant recipients, Gomez-Lobo 2014; Kitano 2023; Kumar 2013; MacIntyre 2016; Miyaji 2024; Moreira dos Santos 2024 | Acute rejection, 1 year after enrolment | 2 | 45 | 4.4 | NA | NA | NA |
|  | Pain at injection site for dose 1-3, 7 days after vaccination | 2 (events) | 23 (doses) | 8.7 | 0 (events) | 57 (doses) | 0.0 |
|  | Swelling and pain at the injection site, any dose, time point: NR | 3 | NR | NA | NA | NA | NA |
|  | Tenderness at the injection site, 48h and 7 days after 1 dose | 10 | 45 | 22.2 | NA | NA | NA |
|  | Tenderness at the injection site, 48h and 7 days after 2 doses | 1 | 45 | 2.2 | NA | NA | NA |
|  | Local adverse events within 2 weeks from baseline vaccination | 16 | 57 | 28.1 | NA | NA | NA |
|  | Local adverse events within 2 weeks from dose 2 vaccination at month 2 | 10 | 55 | 18.2 | NA | NA | NA |
|  | Local adverse events within 2 weeks from dose 2 vaccination at month 6 | 8 | 52 | 15.4 | NA | NA | NA |
|  | Solicited adverse events: Local pain (grade 1) after 1 dose | 34 | 125 | 27.2 | 51 | 132 | 38.6 |
|  | Solicited adverse events: Local pain (grade 1) after 2 doses | 24 | 121 | 19.8 | 36 | 127 | 28.3 |
|  | Solicited adverse events: Local pain (grade 1) after 3 doses | 21 | 113 | 18.6 | 34 | 124 | 27.4 |
|  | Solicited adverse events: Local pain (grade 2) after 1 dose | 3 | 125 | 2.4 | 6 | 132 | 4.5 |
|  | Solicited adverse events: Local pain (grade 2) after 2 doses | 1 | 121 | 0.8 | 10 | 127 | 7.9 |
|  | Solicited adverse events: Local pain (grade 2) after 3 doses | 3 | 113 | 2.7 | 4 | 124 | 3.2 |
|  | Solicited adverse events: Local pain (grade 3) after 1 dose | 2 | 125 | 1.6 | 1 | 132 | 0.8 |
|  | Solicited adverse events: Local pain (grade 3) after 2 doses | 0 | 121 | 0.0 | 0 | 127 | 0.0 |
|  | Solicited adverse events: Local pain (grade 3) after 3 doses | 0 | 113 | 0.0 | 0 | 124 | 0.0 |
|  | Solicited adverse events: Local pain (grade 4) after 1 dose | 0 | 125 | 0.0 | 0 | 132 | 0.0 |
|  | Solicited adverse events: Local pain (grade 4) after 2 doses | 0 | 121 | 0.0 | 0 | 127 | 0.0 |
|  | Solicited adverse events: Local pain (grade 4) after 3 doses | 0 | 113 | 0.0 | 0 | 124 | 0.0 |
|  | Solicited adverse events: Local erythema (grade 1) after 1 dose | 3 | 125 | 2.4 | 7 | 132 | 5.3 |
|  | Solicited adverse events: Local erythema (grade 1) after 2 doses | 7 | 121 | 5.8 | 1 | 127 | 0.8 |
|  | Solicited adverse events: Local erythema (grade 1) after 3 doses | 5 | 113 | 4.4 | 5 | 124 | 4.0 |
|  | Solicited adverse events: Local erythema (grade 2) after 1 dose | 2 | 125 | 1.6 | 3 | 132 | 2.3 |
|  | Solicited adverse events: Local erythema (grade 2) after 2 doses | 0 | 121 | 0.0 | 1 | 127 | 0.8 |
|  | Solicited adverse events: Local erythema (grade 2) after 3 doses | 0 | 113 | 0.0 | 1 | 124 | 0.8 |
|  | Solicited adverse events: Local erythema (grade 3) after 1 dose | 0 | 125 | 0.0 | 0 | 132 | 0.0 |
|  | Solicited adverse events: Local erythema (grade 3) after 2 doses | 0 | 121 | 0.0 | 0 | 127 | 0.0 |
|  | Solicited adverse events: Local erythema (grade 3) after 3 doses | 0 | 113 | 0.0 | 0 | 124 | 0.0 |
|  | Solicited adverse events: Local erythema (grade 4) after 1 dose | 0 | 125 | 0.0 | 0 | 132 | 0.0 |
|  | Solicited adverse events: Local erythema (grade 4) after 2 doses | 0 | 121 | 0.0 | 0 | 127 | 0.0 |
|  | Solicited adverse events: Local erythema (grade 4) after 3 doses | 0 | 113 | 0.0 | 0 | 124 | 0.0 |
|  | Solicited adverse events: Local oedema (grade 1) after 1 dose | 4 | 125 | 3.2 | 10 | 132 | 7.6 |
|  | Solicited adverse events: Local oedema (grade 1) after 2 doses | 5 | 121 | 4.1 | 3 | 127 | 2.4 |
|  | Solicited adverse events: Local oedema (grade 1) after 3 doses | 4 | 113 | 3.5 | 5 | 124 | 4.0 |
|  | Solicited adverse events: Local oedema (grade 2) after 1 dose | 2 | 125 | 1.6 | 2 | 132 | 1.5 |
|  | Solicited adverse events: Local oedema (grade 2) after 2 doses | 1 | 121 | 0.8 | 2 | 127 | 1.6 |
|  | Solicited adverse events: Local oedema (grade 2) after 3 doses | 1 | 113 | 0.9 | 5 | 124 | 4.0 |
|  | Solicited adverse events: Local oedema (grade 3) after 1 dose | 0 | 125 | 0.0 | 0 | 132 | 0.0 |
|  | Solicited adverse events: Local oedema (grade 3) after 2 doses | 0 | 121 | 0.0 | 0 | 127 | 0.0 |
|  | Solicited adverse events: Local oedema (grade 3) after 3 doses | 0 | 113 | 0.0 | 0 | 124 | 0.0 |
|  | Solicited adverse events: Local oedema (grade 4) after 1 dose | 0 | 125 | 0.0 | 0 | 132 | 0.0 |
|  | Solicited adverse events: Local oedema (grade 4) after 2 doses | 0 | 121 | 0.0 | 0 | 127 | 0.0 |
|  | Solicited adverse events: Local oedema (grade 4) after 3 doses | 0 | 113 | 0.0 | 0 | 124 | 0.0 |
|  | Pain at injection site (grade 1), 30 min after the injection of dose 4 | 3 | 23 | 13.0 | NA | NA | NA |
|  | Burning (grade 1), 30 min after the injection of dose 4 | 1 | 23 | 4.3 | NA | NA | NA |
|  | Pain at injection site, 1-2 days after 4 doses | 2 | 23 | 8.7 | NA | NA | NA |
|  | Pain at injection site (grade 1), lasting for 2 days after injection after 4 doses | 1 | 5 | 20.0 | NA | NA | NA |
|  | Unsolicited adverse events: Equimosis at administration site after 1 dose | 2 | NR | NA | 1 | NR | NA |
|  | Unsolicited adverse events: Equimosis at administration site after 2 doses | 0 | NR | NA | 1 | NR | NA |
|  | Unsolicited adverse events: Equimosis at administration site after 3 doses | 1 | NR | NA | 1 | NR | NA |
|  | Unsolicited adverse events: Diffuse pruritus after 1 dose | 1 | NR | NA | 2 | NR | NA |
|  | Unsolicited adverse events: Diffuse pruritus after 2 doses | 1 | NR | NA | 0 | NR | NA |
|  | Unsolicited adverse events: Diffuse pruritus after 3 doses | 2 | NR | NA | 0 | NR | NA |
|  | Unsolicited adverse events: Heat at administration site after 1 dose | 0 | NR | NA | 0 | NR | NA |
|  | Unsolicited adverse events: Heat at administration site after 2 doses | 0 | NR | NA | 0 | NR | NA |
|  | Unsolicited adverse events: Heat at administration site after 3 doses | 1 | NR | NA | 0 | NR | NA |
|  | Unsolicited adverse events: Pruritus after 1 dose | 3 | NR | NA | 1 | NR | NA |
|  | Unsolicited adverse events: Pruritus after 2 doses | 1 | NR | NA | 0 | NR | NA |
|  | Unsolicited adverse events: Pruritus after 3 doses | 1 | NR | NA | 0 | NR | NA |
|  | Pruritus (grade 1), 30 min after the injection of dose 4 | 1 | 23 | 4.3 | NA | NA | NA |
| **Quadrivalent HPV vaccine, systemic adverse events** | | | | | | | |
| Chronic kidney disease, dialysis and kidney transplant, Nailescu 2020; Nelson 2016 | Acute rejection, probably any dose, time point NR | 2 | 23 | 8.6 | NA | NA | NA |
|  | Acute rejection period, 6 months (following last dose) | 2 | 29 | 6.9 | NA | NA | NA |
|  | Headache, probably any dose, time point NR | 2 | 57 | 3.5 | NA | NA | NA |
| Fanconi anaemia  not on immunosuppression, Stratton 2020 | Fatigue or Flu-like symptom (includes any reported fatigue, dizziness, systemic weakness, aches, malaise, lethargy or nausea), 5 days after each vaccination (last dose month 6) | 4 | 21 | 19.0 | 4 | 20 | 20.0 |
|  | Headache, 5 days after each vaccination (last dose month 6) | 5 | 21 | 23.8 | 1 | 20 | 5.0 |
|  | Other event (bladder pain, back pain, oedema, difficulty sleeping), 5 days after each vaccination (last dose month 6) | 2 | 21 | 9.5 | 0 | 20 | 0.0 |
|  | 5 days after each vaccination (last dose month 6) | 0 | 21 | 0.0 | 0 | 20 | 0.0 |
| Fanconi anaemia on immunosuppression, Stratton 2020 | Fatigue or flu-like symptom (includes any reported fatigue, dizziness, systemic weakness, aches, malaise, lethargy or nausea), 5 days after each vaccination (last dose month 6) | 7 | 23 | 30.4 | 4 | 20 | 20.0 |
|  | Headache, 5 days after each vaccination (last dose month 6) | 1 | 23 | 4.3 | 1 | 20 | 5.0 |
|  | Other event (bladder pain, back pain, oedema, difficulty sleeping), 5 days after each vaccination (last dose month 6) | 2 | 23 | 8.7 | 0 | 20 | 0.0 |
|  | 5 days after each vaccination (last dose month 6) | 2 | 23 | 8.7 | 0 | 20 | 0.0 |
| Inflammatory bowel disease, Jacobson 2013 | Abdominal pain (minor adverse event), week 2, (relative to dose) | 3 | NR | NA | NA | NA | NA |
|  | Allergic asthma wheezing, after dose 1 (day 1) | 0 | 35 | 0.0 | NA | NA | NA |
|  | Allergic asthma wheezing, after dose 2 (month 2) | 0 | 32 | 0.0 | NA | NA | NA |
|  | Allergic asthma wheezing, after dose 3 (month 6) | 0 | 33 | 0.0 | NA | NA | NA |
|  | Dizziness, after dose 1 (day 1) | 0 | 35 | 0.0 | NA | NA | NA |
|  | Dizziness, after dose 2 (month 2) | 0 | 32 | 0.0 | NA | NA | NA |
|  | Dizziness, after dose 3 (month 6) | 0 | 33 | 0.0 | NA | NA | NA |
|  | Fatigue, after dose 1 (day 1) | 1 | 35 | 2.9 | NA | NA | NA |
|  | Fatigue, after dose 2 (month 2) | 3 | 32 | 9.4 | NA | NA | NA |
|  | Fatigue, after dose 3 (month 6) | 1 | 33 | 3.0 | NA | NA | NA |
|  | Headache, after dose 1 (day 1) | 3 | 35 | 8.6 | NA | NA | NA |
|  | Headache, after dose 2 (month 2) | 3 | 32 | 9.4 | NA | NA | NA |
|  | Headache, after dose 3 (month 6) | 2 | 33 | 6.1 | NA | NA | NA |
|  | Hives, after dose 1 (day 1) | 0 | 35 | 0.0 | NA | NA | NA |
|  | Hives, after dose 2 (month 2) | 0 | 32 | 0.0 | NA | NA | NA |
|  | Hives, after dose 3 (month 6) | 0 | 33 | 0.0 | NA | NA | NA |
|  | Low grade fever, after dose 1 (day 1) | 0 | 35 | 0.0 | NA | NA | NA |
|  | Low grade fever, after dose 2 (month 2) | 0 | 32 | 0.0 | NA | NA | NA |
|  | Low grade fever, after dose 3 (month 6) | 0 | 33 | 0.0 | NA | NA | NA |
|  | Migraine (minor adverse event), month 3 | 1 | NR | NA | NA | NA | NA |
|  | Nausea, after dose 1 (day 1) | 1 | 35 | 2.9 | NA | NA | NA |
|  | Nausea, after dose 2 (month 2) | 0 | 32 | 0.0 | NA | NA | NA |
|  | Nausea, after dose 3 (month 6) | 3 | 33 | 9.1 | NA | NA | NA |
|  | Rash on their chin (minor adverse event), day 2 (relative to dose) | 1 | NR | NA | NA | NA | NA |
|  | Respiratory distress, after dose 1 (day 1) | 0 | 35 | 0.0 | NA | NA | NA |
|  | Respiratory distress, after dose 2 (month 2) | 0 | 32 | 0.0 | NA | NA | NA |
|  | Respiratory distress, after dose 3 (month 6) | 0 | 33 | 0.0 | NA | NA | NA |
| Juvenile dermatomyositis, Grein 2020b | Fainting, after first dose within 14 days (at baseline) | 0 | 40 | 0.0 | 0 | 38 | 0.0 |
|  | Fainting, after second dose within 14 days (month 1 or 2) | 0 | 41 | 0.0 | 0 | 38 | 0.0 |
|  | Fainting, after third dose within 14 days (month 6) | 0 | 40 | 0.0 | 0 | 35 | 0.0 |
|  | Fatigue, after first dose within 14 days (at baseline) | 6 | 40 | 15.0 | 7 | 38 | 18.4 |
|  | Fatigue, after second dose within 14 days (month 1 or 2) | 4 | 41 | 9.8 | 7 | 38 | 18.4 |
|  | Fatigue, after third dose within 14 days (month 6) | 4 | 40 | 10.0 | 5 | 35 | 14.3 |
|  | Fever, after first dose within 14 days (at baseline) | 1 | 40 | 2.5 | 0 | 38 | 0.0 |
|  | Fever, after second dose within 14 days (month 1 or 2) | 1 | 41 | 2.4 | 0 | 38 | 0.0 |
|  | Fever, after third dose within 14 days (month 6) | 0 | 40 | 0.0 | 1 | 35 | 2.9 |
|  | Headache, after first dose within 14 days (at baseline) | 9 | 40 | 22.5 | 10 | 38 | 26.3 |
|  | Headache, after second dose within 14 days (month 1 or 2) | 10 | 41 | 24.4 | 10 | 38 | 26.3 |
|  | Headache, after third dose within 14 days (month 6) | 6 | 40 | 15.0 | 7 | 35 | 20.0 |
|  | Initial or worsened articular pain, after first dose within 14 days (at baseline) | 1 | 40 | 2.5 | 0 | 38 | 0.0 |
|  | Initial or worsened articular pain, after second dose within 14 days (month 1 or 2) | 1 | 41 | 2.4 | 0 | 38 | 0.0 |
|  | Initial or worsened articular pain, after third dose within 14 days (month 6) | 0 | 40 | 0.0 | 0 | 35 | 0.0 |
|  | Initial or worsened muscular pain, after first dose within 14 days (at baseline) | 2 | 40 | 5.0 | 2 | 38 | 5.3 |
|  | Initial or worsened muscular pain, after second dose within 14 days (month 1 or 2) | 2 | 41 | 4.9 | 1 | 38 | 2.6 |
|  | Initial or worsened muscular pain, after third dose within 14 days (month 6) | 0 | 40 | 0.0 | 0 | 35 | 0.0 |
|  | Itchiness, after first dose within 14 days (at baseline) | 1 | 40 | 2.5 | 1 | 38 | 2.6 |
|  | Itchiness, after second dose within 14 days (month 1 or 2) | 3 | 41 | 7.3 | 1 | 38 | 2.6 |
|  | Itchiness, after third dose within 14 days (month 6) | 7 | 40 | 17.5 | 0 | 35 | 0.0 |
|  | Nausea, after first dose within 14 days (at baseline) | 9 | 40 | 22.5 | 1 | 38 | 2.6 |
|  | Nausea, after second dose within 14 days (month 1 or 2) | 1 | 41 | 2.4 | 2 | 38 | 5.3 |
|  | Nausea, after third dose within 14 days (month 6) | 2 | 40 | 5.0 | 4 | 35 | 11.4 |
|  | New cutaneous abnormalities (patients described new rash on face or on body, that subsided in a maximum of 4 days), after first dose within 14 days (at baseline) | 2 | 40 | 5.0 | 0 | 38 | 0.0 |
|  | New cutaneous abnormalities (patients described new rash on face or on body, that subsided in a maximum of 4 days), after second dose within 14 days (month 1 or 2) | 1 | 41 | 2.4 | 0 | 38 | 0.0 |
|  | New cutaneous abnormalities (patients described new rash on face or on body, that subsided in a maximum of 4 days), after third dose within 14 days (month 6) | 1 | 40 | 2.5 | 0 | 35 | 0.0 |
|  | Vomiting, after first dose within 14 days (at baseline) | 2 | 40 | 5.0 | 0 | 38 | 0.0 |
|  | Vomiting, after second dose within 14 days (month 1 or 2) | 0 | 41 | 0.0 | 0 | 38 | 0.0 |
|  | Vomiting, after third dose within 14 days (month 6) | 0 | 40 | 0.0 | 0 | 35 | 0.0 |
| Survivors of cancer, Landier 2022 | Dizziness, 15 days post any dose | 47 | 253 | 18.6 | NR | 8181 | NA |
|  | Fatigue, 15 days post any dose | 10 | 253 | 4.0 | 670 | 10115 | 6.6 |
|  | Fever ≥37.8°C, 1-5 days post any dose | 53 | 253 | 20.9 | NR | 8181 | NA |
|  | Headache, 15 days post any dose | 29 | 253 | 11.5 | 403 | 8181 | 4.9 |
|  | Nausea, 15 days post any dose | 47 | 253 | 18.6 | NR | 8181 | NA |
| Systemic lupus erythematosus Mok 2013 | Headache, any dose, within 12 months | 1 | 50 | 2.0 | 1 | 50 | 2.0 |
|  | Nausea, any dose, within 12 months | 1 | 50 | 2.0 | 0 | 50 | 0.0 |
| Systemic lupus erythematosus (children), Grein 2020a | Articular pain, after first dose within 14 days (at baseline) | 21 | 179 | 11.7 | 3 | 38 | 7.9 |
|  | Articular pain, after second dose within 14 days (month 1 or 2) | 28 | 182 | 15.4 | 3 | 38 | 7.9 |
|  | Articular pain, after third dose within 14 days (month 6) | 16 | 194 | 8.2 | 2 | 35 | 5.7 |
|  | Fainting, after first dose within 14 days (at baseline) | 0 | 179 | 0.0 | 0 | 38 | 0.0 |
|  | Fainting, after second dose within 14 days (month 1 or 2) | 0 | 182 | 0.0 | 0 | 38 | 0.0 |
|  | Fainting, after third dose within 14 days (month 6) | 0 | 194 | 0.0 | 0 | 35 | 0.0 |
|  | Fatigue, after first dose within 14 days (at baseline) | 48 | 179 | 26.8 | 7 | 38 | 18.4 |
|  | Fatigue, after second dose within 14 days (month 1 or 2) | 37 | 182 | 20.3 | 7 | 38 | 18.4 |
|  | Fatigue, after third dose within 14 days (month 6) | 26 | 194 | 13.4 | 5 | 35 | 14.3 |
|  | Fever, after first dose within 14 days (at baseline) | 3 | 179 | 1.7 | 0 | 38 | 0.0 |
|  | Fever, after second dose within 14 days (month 1 or 2) | 2 | 182 | 1.1 | 0 | 38 | 0.0 |
|  | Fever, after third dose within 14 days (month 6) | 2 | 194 | 1.0 | 1 | 35 | 2.9 |
|  | Headache, after first dose within 14 days (at baseline) | 60 | 179 | 33.5 | 10 | 38 | 26.3 |
|  | Headache, after second dose within 14 days (month 1 or 2) | 42 | 182 | 23.1 | 10 | 38 | 26.3 |
|  | Headache, after third dose within 14 days (month 6) | 30 | 194 | 15.5 | 7 | 35 | 20.0 |
|  | Itchiness, after first dose within 14 days (at baseline) | 9 | 179 | 5.0 | 1 | 38 | 2.6 |
|  | Itchiness, after second dose within 14 days (month 1 or 2) | 3 | 182 | 1.6 | 1 | 38 | 2.6 |
|  | Itchiness, after third dose within 14 days (month 6) | 1 | 194 | 0.5 | 0 | 35 | 0.0 |
|  | Muscular pain, after first dose within 14 days (at baseline) | 26 | 179 | 14.5 | 4 | 38 | 10.5 |
|  | Muscular pain, after second dose within 14 days (month 1 or 2) | 19 | 182 | 10.4 | 4 | 38 | 10.5 |
|  | Muscular pain, after third dose within 14 days (month 6) | 18 | 194 | 9.3 | 2 | 35 | 5.7 |
|  | Nausea, after first dose within 14 days (at baseline) | 28 | 179 | 15.6 | 1 | 38 | 2.6 |
|  | Nausea, after second dose within 14 days (month 1 or 2) | 26 | 182 | 14.3 | 2 | 38 | 5.3 |
|  | Nausea, after third dose within 14 days (month 6) | 14 | 194 | 7.2 | 4 | 35 | 11.4 |
|  | Skin abnormalities, after first dose within 14 days (at baseline) | 9 | 179 | 5.0 | 0 | 38 | 0.0 |
|  | Skin abnormalities, after second dose within 14 days (month 1 or 2) | 3 | 182 | 1.6 | 0 | 38 | 0.0 |
|  | Skin abnormalities, after third dose within 14 days (month 6) | 0 | 194 | 0.0 | 0 | 35 | 0.0 |
|  | Vomiting, after first dose within 14 days (at baseline) | 6 | 179 | 3.4 | 0 | 38 | 0.0 |
|  | Vomiting, after second dose within 14 days (month 1 or 2) | 5 | 182 | 2.7 | 0 | 38 | 0.0 |
|  | Vomiting, after third dose within 14 days (month 6) | 1 | 194 | 0.5 | 0 | 35 | 0.0 |
| Transplant recipients, Gomez-Lobo 2014; Kumar 2013; MacIntyre 2016; Miyaji 2024; Moreira dos Santos 2024 | Dizziness, 48h and 7 days after 1 dose | 1 | 45 | 2.2 | NA | NA | NA |
|  | Fatigue, 48h and 7 days after 1 dose | 4 | 45 | 8.9 | NA | NA | NA |
|  | Fever, 48h and 7 days after 1 dose | 1 | 45 | 2.2 | NA | NA | NA |
|  | Fever, 48h and 7 days after 2 doses | 1 | 45 | 2.2 | NA | NA | NA |
|  | Fever, any dose, time point NR | 4 | NR | NA | NA | NA | NA |
|  | Diarrhoea, any dose, time point NR | 1 | NR | NA | NA | NA | NA |
|  | Headache, any dose, time point NR | 1 | NR | NA | NA | NA | NA |
|  | Headache, 48h and 7 days after 1 dose | 1 | 45 | 2.2 | NA | NA | NA |
|  | Systemic adverse events, within 2 weeks from baseline vaccination | 9 | 57 | 15.8 | NA | NA | NA |
|  | Systemic adverse events, within 2 weeks from dose 2 vaccination at month 2 | 7 | 55 | 12.7 | NA | NA | NA |
|  | Systemic adverse events, within 2 weeks from dose 2 vaccination at month 6 | 3 | 52 | 5.8 | NA | NA | NA |
|  | Solicited adverse events: Myalgia (grade 1) after 1 dose | 11 | 125 | 8.8 | 14 | 132 | 10.6 |
|  | Solicited adverse events: Myalgia (grade 1) after 2 doses | 6 | 121 | 5.0 | 5 | 127 | 3.9 |
|  | Solicited adverse events: Myalgia (grade 1) after 3 doses | 4 | 113 | 3.5 | 5 | 124 | 4.0 |
|  | Solicited adverse events: Myalgia (grade 2) after 1 dose | 1 | 125 | 0.8 | 1 | 132 | 0.8 |
|  | Solicited adverse events: Myalgia (grade 2) after 2 doses | 3 | 121 | 2.5 | 3 | 127 | 2.4 |
|  | Solicited adverse events: Myalgia (grade 2) after 3 doses | 4 | 113 | 3.5 | 5 | 124 | 4.0 |
|  | Solicited adverse events: Myalgia (grade 3) after 1 dose | 1 | 125 | 0.8 | 2 | 132 | 1.5 |
|  | Solicited adverse events: Myalgia (grade 3) after 2 doses | 1 | 121 | 0.8 | 0 | 127 | 0.0 |
|  | Solicited adverse events: Myalgia (grade 3) after 3 doses | 1 | 113 | 0.9 | 0 | 124 | 0.0 |
|  | Solicited adverse events: Myalgia (grade 4) after 1 dose | 1 | 125 | 0.8 | 0 | 132 | 0.0 |
|  | Solicited adverse events: Myalgia (grade 4) after 2 doses | 1 | 121 | 0.8 | 0 | 127 | 0.0 |
|  | Solicited adverse events: Myalgia (grade 4) after 3 doses | 0 | 113 | 0.0 | 0 | 124 | 0.0 |
|  | Solicited adverse events: Headache (grade 1) after 1 dose | 22 | 125 | 17.6 | 20 | 132 | 15.2 |
|  | Solicited adverse events: Headache (grade 1) after 2 doses | 4 | 121 | 3.3 | 17 | 127 | 13.4 |
|  | Solicited adverse events: Headache (grade 1) after 3 doses | 6 | 113 | 5.3 | 11 | 124 | 8.9 |
|  | Solicited adverse events: Headache (grade 2) after 1 dose | 4 | 125 | 3.2 | 10 | 132 | 7.6 |
|  | Solicited adverse events: Headache (grade 2) after 2 doses | 8 | 121 | 6.6 | 5 | 127 | 3.9 |
|  | Solicited adverse events: Headache (grade 2) after 3 doses | 3 | 113 | 2.7 | 6 | 124 | 4.8 |
|  | Solicited adverse events: Headache (grade 3) after 1 dose | 3 | 125 | 2.4 | 5 | 132 | 3.8 |
|  | Solicited adverse events: Headache (grade 3) after 2 doses | 1 | 121 | 0.8 | 0 | 127 | 0.0 |
|  | Solicited adverse events: Headache (grade 3) after 3 doses | 3 | 113 | 2.7 | 2 | 124 | 1.6 |
|  | Solicited adverse events: Headache (grade 4) after 1 dose | 1 | 125 | 0.8 | 0 | 132 | 0.0 |
|  | Solicited adverse events: Headache (grade 4) after 2 doses | 2 | 121 | 1.7 | 0 | 127 | 0.0 |
|  | Solicited adverse events: Headache (grade 4) after 3 doses | 0 | 113 | 0.0 | 0 | 124 | 0.0 |
|  | Solicited adverse events: Nausea (grade 1) after 1 dose | 10 | 125 | 8.0 | 5 | 132 | 3.8 |
|  | Solicited adverse events: Nausea (grade 1) after 2 doses | 5 | 121 | 4.1 | 1 | 127 | 0.8 |
|  | Solicited adverse events: Nausea (grade 1) after 3 doses | 4 | 113 | 3.5 | 1 | 124 | 0.8 |
|  | Solicited adverse events: Nausea (grade 2) after 1 dose | 1 | 125 | 0.8 | 3 | 132 | 2.3 |
|  | Solicited adverse events: Nausea (grade 2) after 2 doses | 2 | 121 | 1.7 | 0 | 127 | 0.0 |
|  | Solicited adverse events: Nausea (grade 2) after 3 doses | 2 | 113 | 1.8 | 2 | 124 | 1.6 |
|  | Solicited adverse events: Nausea (grade 3) after 1 dose | 1 | 125 | 0.8 | 1 | 132 | 0.8 |
|  | Solicited adverse events: Nausea (grade 3) after 2 doses | 0 | 121 | 0.0 | 0 | 127 | 0.0 |
|  | Solicited adverse events: Nausea (grade 3) after 3 doses | 2 | 113 | 1.8 | 0 | 124 | 0.0 |
|  | Solicited adverse events: Nausea (grade 4) after 1 dose | 0 | 125 | 0.0 | 0 | 132 | 0.0 |
|  | Solicited adverse events: Nausea (grade 4) after 2 doses | 0 | 121 | 0.0 | 0 | 127 | 0.0 |
|  | Solicited adverse events: Nausea (grade 4) after 3 doses | 0 | 113 | 0.0 | 0 | 124 | 0.0 |
|  | Solicited adverse events: Vomiting (grade 1) after 1 dose | 1 | 125 | 0.8 | 0 | 132 | 0.0 |
|  | Solicited adverse events: Vomiting (grade 1) after 2 doses | 2 | 121 | 1.7 | 0 | 127 | 0.0 |
|  | Solicited adverse events: Vomiting (grade 1) after 3 doses | 0 | 113 | 0.0 | 0 | 124 | 0.0 |
|  | Solicited adverse events: Vomiting (grade 2) after 1 dose | 0 | 125 | 0.0 | 2 | 132 | 1.5 |
|  | Solicited adverse events: Vomiting (grade 2) after 2 doses | 0 | 121 | 0.0 | 0 | 127 | 0.0 |
|  | Solicited adverse events: Vomiting (grade 2) after 3 doses | 0 | 113 | 0.0 | 1 | 124 | 0.8 |
|  | Solicited adverse events: Vomiting (grade 3) after 1 dose | 1 | 125 | 0.8 | 1 | 132 | 0.8 |
|  | Solicited adverse events: Vomiting (grade 3) after 2 doses | 0 | 121 | 0.0 | 0 | 127 | 0.0 |
|  | Solicited adverse events: Vomiting (grade 3) after 3 doses | 2 | 113 | 1.8 | 0 | 124 | 0.0 |
|  | Solicited adverse events: Vomiting (grade 4) after 1 dose | 0 | 125 | 0.0 | 0 | 132 | 0.0 |
|  | Solicited adverse events: Vomiting (grade 4) after 2 doses | 1 | 121 | 0.8 | 0 | 127 | 0.0 |
|  | Solicited adverse events: Vomiting (grade 4) after 3 doses | 0 | 113 | 0.0 | 0 | 124 | 0.0 |
|  | Solicited adverse events: Malaise (grade 1) after 1 dose | 8 | 125 | 6.4 | 10 | 132 | 7.6 |
|  | Solicited adverse events: Malaise (grade 1) after 2 doses | 1 | 121 | 0.8 | 5 | 127 | 3.9 |
|  | Solicited adverse events: Malaise (grade 1) after 3 doses | 1 | 113 | 0.9 | 3 | 124 | 2.4 |
|  | Solicited adverse events: Malaise (grade 2) after 1 dose | 0 | 125 | 0.0 | 4 | 132 | 3.0 |
|  | Solicited adverse events: Malaise (grade 2) after 2 doses | 4 | 121 | 3.3 | 2 | 127 | 1.6 |
|  | Solicited adverse events: Malaise (grade 2) after 3 doses | 3 | 113 | 2.7 | 6 | 124 | 4.8 |
|  | Solicited adverse events: Malaise (grade 3) after 1 dose | 2 | 125 | 1.6 | 2 | 132 | 1.5 |
|  | Solicited adverse events: Malaise (grade 3) after 2 doses | 0 | 121 | 0.0 | 1 | 127 | 0.8 |
|  | Solicited adverse events: Malaise (grade 3) after 3 doses | 1 | 113 | 0.9 | 0 | 124 | 0.0 |
|  | Solicited adverse events: Malaise (grade 4) after 1 dose | 0 | 125 | 0.0 | 0 | 132 | 0.0 |
|  | Solicited adverse events: Malaise (grade 4) after 2 doses | 0 | 121 | 0.0 | 0 | 127 | 0.0 |
|  | Solicited adverse events: Malaise (grade 4) after 3 doses | 0 | 113 | 0.0 | 0 | 124 | 0.0 |
|  | Solicited adverse events: Somnolence (grade 1) after 1 dose | 13 | 125 | 10.4 | 21 | 132 | 15.9 |
|  | Solicited adverse events: Somnolence (grade 1) after 2 doses | 4 | 121 | 3.3 | 10 | 127 | 7.9 |
|  | Solicited adverse events: Somnolence (grade 1) after 3 doses | 1 | 113 | 0.9 | 6 | 124 | 4.8 |
|  | Solicited adverse events: Somnolence (grade 2) after 1 dose | 1 | 125 | 0.8 | 3 | 132 | 2.3 |
|  | Solicited adverse events: Somnolence (grade 2) after 2 doses | 1 | 121 | 0.8 | 2 | 127 | 1.6 |
|  | Solicited adverse events: Somnolence (grade 2) after 3 doses | 0 | 113 | 0.0 | 5 | 124 | 4.0 |
|  | Solicited adverse events: Somnolence (grade 3) after 1 dose | 1 | 125 | 0.8 | 2 | 132 | 1.5 |
|  | Solicited adverse events: Somnolence (grade 3) after 2 doses | 0 | 121 | 0.0 | 0 | 127 | 0.0 |
|  | Solicited adverse events: Somnolence (grade 3) after 3 doses | 3 | 113 | 2.7 | 1 | 124 | 0.8 |
|  | Solicited adverse events: Somnolence (grade 4) after 1 dose | 0 | 125 | 0.0 | 0 | 132 | 0.0 |
|  | Solicited adverse events: Somnolence (grade 4) after 2 doses | 0 | 121 | 0.0 | 0 | 127 | 0.0 |
|  | Solicited adverse events: Somnolence (grade 4) after 3 doses | 0 | 113 | 0.0 | 0 | 124 | 0.0 |
|  | Solicited adverse events: Dizziness (grade 1) after 1 dose | 6 | 125 | 4.8 | 4 | 132 | 3.0 |
|  | Solicited adverse events: Dizziness (grade 1) after 2 doses | 2 | 121 | 1.7 | 0 | 127 | 0.0 |
|  | Solicited adverse events: Dizziness (grade 1) after 3 doses | 3 | 113 | 2.7 | 2 | 124 | 1.6 |
|  | Solicited adverse events: Dizziness (grade 2) after 1 dose | 0 | 125 | 0.0 | 1 | 132 | 0.8 |
|  | Solicited adverse events: Dizziness (grade 2) after 2 doses | 1 | 121 | 0.8 | 4 | 127 | 3.1 |
|  | Solicited adverse events: Dizziness (grade 2) after 3 doses | 0 | 113 | 0.0 | 0 | 124 | 0.0 |
|  | Solicited adverse events: Dizziness (grade 3) after 1 dose | 0 | 125 | 0.0 | 2 | 132 | 1.5 |
|  | Solicited adverse events: Dizziness (grade 3) after 2 doses | 1 | 121 | 0.8 | 0 | 127 | 0.0 |
|  | Solicited adverse events: Dizziness (grade 3) after 3 doses | 2 | 113 | 1.8 | 0 | 124 | 0.0 |
|  | Solicited adverse events: Dizziness (grade 4) after 1 dose | 0 | 125 | 0.0 | 0 | 132 | 0.0 |
|  | Solicited adverse events: Dizziness (grade 4) after 2 doses | 0 | 121 | 0.0 | 0 | 127 | 0.0 |
|  | Solicited adverse events: Dizziness (grade 4) after 3 doses | 0 | 113 | 0.0 | 0 | 124 | 0.0 |
|  | Solicited adverse events: Fever (grade 1) after 1 dose | 1 | 125 | 0.8 | 1 | 132 | 0.8 |
|  | Solicited adverse events: Fever (grade 1) after 2 doses | 2 | 121 | 1.7 | 2 | 127 | 1.6 |
|  | Solicited adverse events: Fever (grade 1) after 3 doses | 0 | 113 | 0.0 | 1 | 124 | 0.8 |
|  | Solicited adverse events: Fever (grade 2) after 1 dose | 1 | 125 | 0.8 | 0 | 132 | 0.0 |
|  | Solicited adverse events: Fever (grade 2) after 2 doses | 0 | 121 | 0.0 | 0 | 127 | 0.0 |
|  | Solicited adverse events: Fever (grade 2) after 3 doses | 0 | 113 | 0.0 | 0 | 124 | 0.0 |
|  | Solicited adverse events: Fever (grade 3) after 1 dose | 0 | 125 | 0.0 | 1 | 132 | 0.8 |
|  | Solicited adverse events: Fever (grade 3) after 2 doses | 0 | 121 | 0.0 | 0 | 127 | 0.0 |
|  | Solicited adverse events: Fever (grade 3) after 3 doses | 0 | 113 | 0.0 | 0 | 124 | 0.0 |
|  | Solicited adverse events: Fever (grade 4) after 1 dose | 0 | 125 | 0.0 | 0 | 132 | 0.0 |
|  | Solicited adverse events: Fever (grade 4) after 2 doses | 0 | 121 | 0.0 | 0 | 127 | 0.0 |
|  | Solicited adverse events: Fever (grade 4) after 3 doses | 0 | 113 | 0.0 | 0 | 124 | 0.0 |
|  | Headache and diarrhea after dose 4 | 1 | 23 | 4.3 | NA | NA | NA |
|  | Headache, joint pain, myalgia, tiredness, and nausea, 7 days after dose 4 | 1 | 5 | 20.0 | NA | NA | NA |
|  | Unsolicited adverse events: Malaise after 1 dose | 1 | NR | NA | 2 | NR | NA |
|  | Unsolicited adverse events: Malaise after 2 doses | 0 | NR | NA | 0 | NR | NA |
|  | Unsolicited adverse events: Malaise after 3 doses | 0 | NR | NA | 0 | NR | NA |
|  | Unsolicited adverse events: Abdominal pain after 1 dose | 2 | NR | NA | 2 | NR | NA |
|  | Unsolicited adverse events: Abdominal pain after 2 doses | 3 | NR | NA | 1 | NR | NA |
|  | Unsolicited adverse events: Abdominal pain after 3 doses | 0 | NR | NA | 0 | NR | NA |
|  | Unsolicited adverse events: Diarrhea after 1 dose | 6 | NR | NA | 6 | NR | NA |
|  | Unsolicited adverse events: Diarrhea after 2 doses | 3 | NR | NA | 11 | NR | NA |
|  | Unsolicited adverse events: Diarrhea after 3 doses | 7 | NR | NA | 3 | NR | NA |
|  | Unsolicited adverse events: Exanthema after 1 dose | 2 | NR | NA | 3 | NR | NA |
|  | Unsolicited adverse events: Exanthema after 2 doses | 1 | NR | NA | 1 | NR | NA |
|  | Unsolicited adverse events: Exanthema after 3 doses | 1 | NR | NA | 2 | NR | NA |
|  | Unsolicited adverse events: Flu-like symptoms after 1 dose | 10 | NR | NA | 2 | NR | NA |
|  | Unsolicited adverse events: Flu-like symptoms after 2 doses | 3 | NR | NA | 1 | NR | NA |
|  | Unsolicited adverse events: Flu-like symptoms after 3 doses | 1 | NR | NA | 0 | NR | NA |
|  | Unsolicited adverse events: Lipothymia after 1 dose | 1 | NR | NA | 1 | NR | NA |
|  | Unsolicited adverse events: Lipothymia after 2 doses | 1 | NR | NA | 0 | NR | NA |
|  | Unsolicited adverse events: Lipothymia after 3 doses | 0 | NR | NA | 0 | NR | NA |
|  | Unsolicited adverse events: Wheezing after 1 dose | 1 | NR | NA | 0 | NR | NA |
|  | Unsolicited adverse events: Wheezing after 2 doses | 2 | NR | NA | 0 | NR | NA |
|  | Unsolicited adverse events: Wheezing after 3 doses | 2 | NR | NA | 1 | NR | NA |
| **Quadrivalent vaccine, additional adverse events** | | | | | | | |
| Inflammatory bowel disease, Jacobson 2013 | Asthma-related (minor AE), day 5 (relative to dose) | 1 | NR | NA | NA | NA | NA |
|  | Axillary abscess, day 9 (relative to vaccine) | 1 | NR | NA | NA | NA | NA |
|  | Leg pain, day 1 (relative to dose) | 1 | NR | NA | NA | NA | NA |
|  | Rectal bleeding and diarrhoea, days 2 and 23 (relative to dose) | 2 | NR | NA | NA | NA | NA |
| Survivors of cancer, Landier 2022 | >1 AE (any type), across all time points | 129 | 253 | 51.0 | NR | NR | NR |
| Systemic lupus erythematosus, Dhar 2017;  Mok 2013 | Irregular menses, any dose, within 12 months | 1 | 50 | 2.0 | 1 | 50 | 2.0 |
|  | General disorders (events; none related to vaccine or SLE), any dose, across all time points | 45 (events) | 34 | NA | NA | NA | NA |
|  | Musculoskeletal (events; none related to vaccine or SLE), any dose, across all time points | 106 (events) | 34 | NA | NA | NA | NA |
|  | Nervous system (mostly headaches) (events; none related to vaccine or SLE), any dose, across all time points | 98 (events) | 34 | NA | NA | NA | NA |
|  | Total (events; none related to vaccine or SLE), any dose, across all time points | 493 (events) | 33 | NA | NA | NA | NA |
|  | Upper respiratory tract infection, any dose, within 12 months | 1 | 50 | 2.0 | 1 | 50 | 2.0 |
| Transplant recipients, Gomez-Lobo 2014; Miyaji 2024; Moreira dos Santos 2024 | Acne, probably any dose, time point NR | 1 | NR | NA | NA | NA | NA |
|  | Cough, probably any dose, time point NR | 1 | NR | NA | NA | NA | NA |
|  | Pneumonia, probably any dose, time point NR | 1 | NR | NA | NA | NA | NA |
|  | Unsolicited adverse events: Altered menstruation after 1 dose | 3 | NR | NA | 5 | NR | NA |
|  | Unsolicited adverse events: Altered menstruation after 2 doses | 1 | NR | NA | 0 | NR | NA |
|  | Unsolicited adverse events: Altered menstruation after 3 doses | 0 | NR | NA | 0 | NR | NA |
|  | Unsolicited adverse events: Lip or eyelid oedema after 1 dose | 2 | NR | NA | 1 | NR | NA |
|  | Unsolicited adverse events: Lip or eyelid oedema after 2 doses | 1 | NR | NA | 1 | NR | NA |
|  | Unsolicited adverse events: Lip or eyelid oedema after 3 doses | 1 | NR | NA | 3 | NR | NA |
|  | Unsolicited adverse events: Artralgia after 1 dose | 1 | NR | NA | 0 | NR | NA |
|  | Unsolicited adverse events: Artralgia after 2 doses | 1 | NR | NA | 0 | NR | NA |
|  | Unsolicited adverse events: Artralgia after 3 doses | 1 | NR | NA | 0 | NR | NA |
|  | Unsolicited adverse events: Menstrual cramps after 1 dose | 2 | NR | NA | 0 | NR | NA |
|  | Unsolicited adverse events: Menstrual cramps after 2 doses | 0 | NR | NA | 1 | NR | NA |
|  | Unsolicited adverse events: Menstrual cramps after 3 doses | 0 | NR | NA | 0 | NR | NA |
|  | Unsolicited adverse events: Other events after 1 dose | 11 | NR | NA | 10 | NR | NA |
|  | Unsolicited adverse events: Other events after 2 doses | 1 | NR | NA | 3 | NR | NA |
|  | Unsolicited adverse events: Other events after 3 doses | 0 | NR | NA | 2 | NR | NA |
|  | Any AE, 7 days after administration 4 doses | 3 | 23 | 13.0 | NA | NA | NA |
| **Nonavalent vaccine, local adverse events** | | | | | | | |
| Survivors of cancer, Landier 2022 | Oedema, any dose, days 1-5 post any dose (with available safety data) | 15 | 182 | 8.2 | 5698 | 15776 | 36.1 |
|  | Erythema, any dose, days 1-5 post any dose (with available safety data) | 17 | 182 | 9.3 | 4859 | 15776 | 30.8 |
|  | Pain, any dose, days 1-5 post any dose (with available safety data) | 84 | 182 | 46.2 | 13118 | 15776 | 83.2 |
| Transplant recipients, Boey 2021 | Bruise, any dose, days 1–5 following any vaccination visit | 0 | 170 | 0.0 | NA | NA | NA |
|  | Oedema, any dose, days 1–5 following any vaccination visit | 14 | 170 | 8.2 | NA | NA | NA |
|  | Oedema: swelling, mild (0 to ≤2.5 cm), any dose, days 1–5 following any vaccination visit | 13 | 170 | 7.6 | NA | NA | NA |
|  | Oedema: swelling, moderate (>2.5 to ≤5.0 cm), any dose, days 1–5 following any vaccination visit | 2 | 170 | 1.2 | NA | NA | NA |
|  | Oedema: swelling, severe (<5.0 cm), any dose, days 1–5 following any vaccination visit | 1 | 170 | 0.6 | NA | NA | NA |
|  | Erythema, any dose, days 1–5 following any vaccination visit | 10 | 170 | 5.9 | NA | NA | NA |
|  | Erythema, mild (0 to ≤2.5 cm) any dose, days 1–5 following any vaccination visit | 9 | 170 | 5.3 | NA | NA | NA |
|  | Erythema, moderate (>2.5 to ≤5.0 cm), any dose, days 1–5 following any vaccination visit | 10 | 170 | 5.9 | NA | NA | NA |
|  | Erythema, severe (<5.0 cm), any dose, days 1–5 following any vaccination visit | 0 | 170 | 0.0 | NA | NA | NA |
|  | Induration, any dose, days 1–5 following any vaccination visit | 0 | 170 | 0.0 | NA | NA | NA |
|  | Pruritus, any dose, days 1–5 following any vaccination visit | 3 | 170 | 1.8 | NA | NA | NA |
|  | Other, any dose, days 1–5 following any vaccination visit | 98 | 170 | 57.6 | NA | NA | NA |
|  | Other, any dose, days 1–5 following any vaccination visit | 14 | 170 | 8.2 | NA | NA | NA |
|  | Pain, any dose, days 1–5 following any vaccination visit | 93 | 170 | 54.7 | NA | NA | NA |
|  | Pain, mild, any dose, days 1–5 following any vaccination visit | 93 | 170 | 54.7 | NA | NA | NA |
|  | Pain, moderate, any dose, days 1–5 following any vaccination visit | 15 | 170 | 8.8 | NA | NA | NA |
|  | Pain, severe, any dose, days 1–5 following any vaccination visit | 0 | 170 | 0.0 | NA | NA | NA |
| **Nonavalent HPV vaccine, systemic adverse events** | | | | | | | |
| Survivors of cancer, Landier 2022 | Dizziness, 15 days post any dose | 2 | 182 | 1.1 | 355 | 15776 | 2.3 |
|  | Fatigue, 15 days post any dose | 24 | 182 | 13.2 | 294 | 15776 | 1.9 |
|  | Fever (≥37.8°C), 1-5 days post any dose | 12 | 182 | 6.6 | 661 | 9354 | 7.1 |
|  | Headache, 15 days post any dose | 33 | 182 | 18.1 | 2090 | 15776 | 13.2 |
|  | Nausea, 15 days post any dose | 21 | 182 | 11.5 | 503 | 15776 | 3.2 |
| Transplant recipients, Boey 2021, Miyaji 2024, Moreira dos Santos 2024 | All systemic events, days 1–15 following any vaccination visit | 74 | 170 | 43.5 | NA | NA | NA |
|  | Vaccine-related systemic events, days 1–15 following any vaccination visit | 35 | 170 | 20.6 | NA | NA | NA |
|  | Dizziness, vaccine-related, days 1–15 following any vaccination visit | 0 | 170 | 0.0 | NA | NA | NA |
|  | Fatigue, vaccine-related, days 1–15 following any vaccination visit | 5 | 170 | 2.9 | NA | NA | NA |
|  | Headache, vaccine-related, days 1–15 following any vaccination visit | 14 | 170 | 8.2 | NA | NA | NA |
|  | Nausea, vaccine-related, days 1–15 following any vaccination visit | 4 | 170 | 2.4 | NA | NA | NA |
|  | Pyrexia (≥37.8°C), days 1–15 following any vaccination visit | 2 | 170 | 1.2 | NA | NA | NA |
|  | Other vaccine-related systemic events, days 1–15 following any vaccination visit | 23 | 170 | 13.5 | 23 | 170 | 13.5 |

AE: adverse event; CG: control group; IG: intervention group; N: number; NA: not applicable; NR: not reported; SLE: Systemic lupus erythematosus

# **Additional summary of findings tables: comparison 3**

**Supplement Table S14:** **Summary of findings, vaccinated immunocompromised group vs. vaccinated healthy control group (comparison 3)
Outcome: Seropositivity, 12 months and more**

| **HPV type** | **Studies (participants)** | **Relative effect (95% CI)** | **Absolute effects (95% CI)** | | | **Certainty** | **Interpretation** |
| --- | --- | --- | --- | --- | --- | --- | --- |
|  |  |  | **Vaccinated healthy control group** | **Vaccinated immunocompromised group** | **Risk difference** |  |  |
| **Participants: Fanconi anaemia (FA)** | | | | | | | |
| **HPV 16** | 1 NRSI (60 participants in intervention group, 21 in control group), Sauter 2021 | **RR 0.98** (0.83 to 1.15) | 905 per 1,000 | **887 per 1,000** (751 to 1,000) | **18 fewer per 1,000** (from 154 fewer to 136 more) | ⨁◯◯◯ Very low^a,b^ | The evidence is of very low certainty about the effect of HPV vaccination on seropositivity rates for HPV 16. There may be little to no difference in seropositivity rates for HPV 16 between Fanconi anaemia and healthy participants. |
| **HPV 18** | 1 NRSI (60 participants in intervention group, 21 in control group), Sauter 2021 | **RR 0.81** (0.66 to 1.00) | 905 per 1,000 | **733 per 1,000** (597 to 905) | **172 fewer per 1000** (from 308 fewer to 0 fewer) | ⨁◯◯◯ Very low^a, c^ | The evidence is of very low certainty about the effect of HPV vaccination on seropositivity rates for HPV 18. There may be reduced seropositivity rates to no differences in Fanconi anaemia compared to healthy participants. |
| **Participants:** **Juvenile idiopathic arthritis (JIA)** | | | | | | | |
| **HPV 16** | 1 NRSI (43 participants in intervention group, 44 in control group), Heijstek 2014 | **RR 0.98** (0.93 to 1.02) | 1,000 per 1,000 | **980 per 1,000** (930 to 1,000) | **20 fewer per 1,000** (from 70 fewer to 20 more) | ⨁⨁◯◯ Low^d,b^ | The evidence is of low certainty, but suggests that there may be little to no difference in seropositivity rates for HPV 16 between JIA and healthy participants. |
| **HPV 18** | 1 NRSI (43 participants in intervention group, 44 in control group), Heijstek 2014 | **RR 0.98** (0.93 to 1.02) | 1,000 per 1,000 | **980 per 1,000** (930 to 1,000) | **20 fewer per 1,000** (from 70 fewer to 20 more) | ⨁⨁◯◯ Low^d,b^ | The evidence is of low certainty, but suggests that there may be little to no difference in seropositivity rates for HPV 18 between JIA and healthy participants. |
| **Participants: Systemic lupus erythematosus (SLE)** | | | | | | | |
| **HPV 16** | 1 NRSI (39 participants in intervention group, 44 in control group), Mok 2013 | **RR 0.97** (0.89 to 1.06) | 977 per 1,000 | **948 per 1,000** (870 to 1,000) | **29 fewer per 1,000** (from 107 fewer to 59 more) | ⨁⨁◯◯ Low^d,b^ | The evidence is of low certainty, but suggests that there may be little to no difference in seropositivity rates for HPV 16 between SLE and healthy participants. |
| **HPV 18** | 1 NRSI (38 participants in intervention group, 40 in control group), Mok 2013 | **RR 0.95** (0.75 to 1.21) | 800 per 1,000 | **760 per 1,000** (600 to 968) | **40 fewer per 1,000** (from 200 fewer to 168 more) | ⨁◯◯◯ Very low^d,c^ | The evidence is of very low certainty about the effect of HPV vaccination on seropositivity rates for HPV 18. There may be reduced or slightly increased seropositivity rates in SLE compared to healthy participants. |
| **Participants: Transplant recipients (kidney)** | | | | | | | |
| **HPV 16** | 1 NRSI (6 participants in intervention group, 13 in control group), Kitano 2023 | **RR 0.69** (0.41 to 1.16) | 1,000 per 1,000 | **690 per 1,000** (410 to 1,000) | **310 fewer per 1,000** (from 590 fewer to 160 more) | ⨁◯◯◯ Very low^d,c^ | The evidence is of very low certainty about the effect of HPV vaccination on seropositivity rates for HPV 16. There may be reduced or slightly increased seropositivity rates in kidney transplant compared to healthy participants. |
| **HPV 18** | 1 NRSI (6 participants in intervention group, 13 in control group), Kitano 2023 | **RR 0.69** (0.41 to 1.16) | 1,000 per 1,000 | **690 per 1,000** (410 to 1,000) | **310 fewer per 1,000** (from 590 fewer to 160 more) | ⨁◯◯◯ Very low^d,c^ | The evidence is of very low certainty about the effect of HPV vaccination on seropositivity rates for HPV 18. There may be reduced or slightly increased seropositivity rates in kidney transplant compared to healthy participants. |
| **Participants: Transplant recipients (liver)** | | | | | | | |
| **HPV 16** | 1 NRSI (6 participants in intervention group, 13 in control group), Kitano 2023 | **RR 1.00** (0.78 to 1.28) | 1,000 per 1,000 | **1000 per 1,000** (780 to 1,000) | **0 fewer per 1,000** (from 220 fewer to 280 more) | ⨁◯◯◯ Very low^d,c^ | The evidence is of very low certainty about the effect of HPV vaccination on seropositivity rates for HPV 16. There may be reduced or increased seropositivity rates in liver transplant compared to healthy participants |
| **HPV 18** | 1 NRSI (6 participants in intervention group, 13 in control group), Kitano 2023 | **RR 0.85** (0.61 to 1.17) | 1,000 per 1,000 | **850 per 1,000** (610 to 1,000) | **150 fewer per 1,000** (from 390 fewer to 170 more) | ⨁◯◯◯ Very low^d,c^ | The evidence is of very low certainty about the effect of HPV vaccination on seropositivity rates for HPV 18. There may be reduced or slightly increased seropositivity rates in liver transplant compared to healthy participants |

CI: confidence interval; FA: Fanconi anaemia; HPV: human papillomavirus; IBD: inflammatory bowel disease; JIA: juvenile idiopathic arthritis; NRSI: non-randomised studies of interventions; RR: risk ratio; SLE: systemic lupus erythematosus

^a^ Risk of bias downgraded by two levels: due to very serious concerns regarding confounding.
^b^ Imprecision downgraded by one level: due to absolute differences that indicate fewer or more events and imprecision due to a small sample size.

^c^ Imprecision downgraded by two levels: due to absolute differences that indicate considerably fewer or more events and imprecision due to a small sample size.

^d^ Risk of bias downgraded by one level: mainly due to serious concerns regarding confounding, selection of participants into the study, deviations from intended interventions or missing outcome data.

**Supplement Table S15: Summary of findings, vaccinated immunocompromised group vs. vaccinated healthy control group (comparison 3)
Outcome: Geometric mean ratio (GMR)**

| **HPV type, follow-up** | **Studies (participants)** | **GMR (based on GMTs) (95%-CI)** | **Certainty** | **Interpretation** |
| --- | --- | --- | --- | --- |
| **Participants: Cancer survivors^*^** | | | | |
| **HPV 16, 7 months** | 1 NRSI (358 participants in intervention group, 14923 in control group), Landier 2022 | **GMR 2.59**  (2.05 to 3.26) | ⨁⨁◯◯ Low^a^ | The evidence is of low certainty, but suggests that there may be higher antibody titres (GMTs) for HPV 16 in cancer survivors compared to healthy participants. |
| **HPV 18, 7 months** | 1 NRSI (369 participants in intervention group, 15834 in control group), Landier 2022 | **GMR 2.52**  (1.94 to 3.27) | ⨁⨁◯◯ Low^a^ | The evidence is of low certainty, but suggests that there may be higher antibody titres (GMTs) for HPV 18 in cancer survivors compared to healthy participants. |
| **Participants: Fanconi anaemia (FA)** | | | | |
| **HPV 16, 12 months and more** | 1 NRSI (60 participants in intervention group, 21 in control group), Sauter 2021 | **GMR 0.59**  (0.13 to 2.64) | ⨁◯◯◯ Very low^a,b^ | The evidence is of very low certainty about the effect of HPV vaccination on GMR for HPV 16. There may be large effects in both directions. |
| **HPV 18, 12 months and more** | 1 NRSI (60 participants in intervention group, 21 in control group), Sauter 2021 | **GMR 0.56**  (0.12 to 2.58) | ⨁◯◯◯ Very low^a,b^ | The evidence is of very low certainty about the effect of HPV vaccination on GMR for HPV 18. There may be large effects in both directions. |
| **Participants: Inflammatory bowel disease (IBD)** | | | | |
| **HPV 16, 7 months** | 1 NRSI (33 participants in intervention group, 4168 in control group), Jacobson 2013 | **GMR 1.06**  (0.60 to 1.88) | ⨁⨁◯◯ Low^c,d^ | The evidence is of low certainty, but suggests that there may be a little to no difference in antibody titres (GMTs) for HPV 16 between IBD and healthy participants. |
| **HPV 18, 7 months** | 1 NRSI (33 participants in intervention group, 4493 in control group), Jacobson 2013 | **GMR 1.12**  (0.62 to 2.02) | ⨁⨁◯◯ Low^c,d^ | The evidence is of low certainty, but suggests that there may be higher antibody titres (GMTs) to no difference for HPV 18 in IBD compared to healthy participants. |
| **Participants:** **Juvenile idiopathic arthritis (JIA)** | | | | |
| **HPV 16, 7 months** | 1 NRSI (41 participants in intervention group, 41 in control group), Heijstek 2014 | **GMR 0.40**  (0.20 to 0.82) | ⨁⨁◯◯ Low^c,d^ | The evidence is of low certainty, but suggests that there may be higher antibody titres (GMTs) for HPV 16 in healthy participants compared to JIA. |
| **HPV 16, 12 months and more** | 1 NRSI (43 participants in intervention group, 44 in control group), Heijstek 2014 | **GMR 0.44**  (0.22 to 0.87) | ⨁⨁◯◯ Low^c,d^ | The evidence is of low certainty, but suggests that there may be higher antibody titres (GMTs) for HPV 16 in healthy participants compared to JIA. |
| **HPV 18, 7 months** | 1 NRSI (41 participants in intervention group, 41 in control group), Heijstek 2014 | **GMR 0.52**  (0.27 to 1.01) | ⨁⨁◯◯ Low^c,d^ | The evidence is of low certainty, but suggests that there may be higher antibody titres (GMTs) to no difference for HPV 18 in healthy participants compared to JIA. |
| **HPV 18, 12 months and more** | 1 NRSI (43 participants in intervention group, 44 in control group), Heijstek 2014 | **GMR 0.63**  (0.31 to 1.25) | ⨁⨁◯◯ Low^c,d^ | The evidence is of low certainty, but suggests that there may be higher antibody titres (GMTs) to no difference for HPV 18 in healthy participants compared to JIA. |
| **Participants: Allogeneic hematopoietic stem cell transplant (post-HSCT)** | | | | |
| **HPV 16, 7 months** | 1 NRSI (44 participants in intervention group, 20 in control group), Stratton 2020 | **GMR 0.88**  (0.40 to 1.97) | ⨁◯◯◯ Very low^c,b^ | The evidence is of very low certainty about the effect of HPV vaccination on GMR for HPV 16. There may be large effects in both directions. |
| **HPV 16, 12 months and more** | 1 NRSI (44 participants in intervention group, 20 in control group), Stratton 2020 | **GMR 0.86**  (0.40 to 1.85) | ⨁⨁◯◯ Low^c,d^ | The evidence is of low certainty, but suggests that there may be higher antibody titres (GMTs) to no difference for HPV 16 in healthy participants compared to post-HSCT. |
| **HPV 18, 7 months** | 1 NRSI (44 participants in intervention group, 20 in control group), Stratton 2020 | **GMR 0.74**  (0.37 to 1.48) | ⨁⨁◯◯ Low^c,d^ | The evidence is of low certainty, but suggests that there may be higher antibody titres (GMTs) to no difference for HPV 18 in healthy participants compared to post-HSCT. |
| **HPV 18, 12 months and more** | 1 NRSI (44 participants in intervention group, 20 in control group), Stratton 2020 | **GMR 0.88**  (0.46 to 1.70) | ⨁⨁◯◯ Low^c,d^ | The evidence is of low certainty, but suggests that there may be higher antibody titres (GMTs) to no difference for HPV 18 in healthy participants compared to post-HSCT. |
| **Participants: Systemic lupus erythematosus (SLE)** | | | | |
| **HPV 16, 7 months** | 1 NRSI (19 participants in intervention group, 657 in control group), Dhar 2017 | **GMR 1.43**  (1.02 to 2.02) | ⨁◯◯◯ Very low^a,d^ | The evidence is of very low certainty about the effect of HPV vaccination on GMR for HPV 16. There may be higher antibody titres (GMTs) to no difference for HPV 16 in SLE compared to healthy participants. |
| **HPV 18, 7 months** | 1 NRSI (27 participants in intervention group, 722 in control group), Dhar 2017 | **GMR 1.75**  (1.23 to 2.48) | ⨁◯◯◯ Very low^a,d^ | The evidence is of very low certainty about the effect of HPV vaccination on GMR for HPV 18. There may be higher antibody titres (GMTs) to no difference for HPV 18 in SLE compared to healthy participants. |
| **Participants: Transplant recipients (kidney, kidney + pancreas, liver, lung, heart)** | | | | |
| **HPV 16, 7 months** | 1 NRSI (81 participants in intervention group, 88 in control group), Miyaji 2024 | **GMR 0.13**  (0.07 to 0.25) | ⨁◯◯◯ Very low^a,e^ | The evidence is of very low certainty about the effect of HPV vaccination on GMR for HPV 16. There may be higher antibody titres (GMTs) for HPV 16 in healthy participants compared to transplant recipients. |
| **HPV 18, 7 months** | 1 NRSI (67 participants in intervention group, 96 in control group), Miyaji 2024 | **GMR 0.13**  (0.07 to 0.26) | ⨁◯◯◯ Very low^a,e^ | The evidence is of very low certainty about the effect of HPV vaccination on GMR for HPV 18. There may be higher antibody titres (GMTs) for HPV 18 in healthy participants compared to transplant recipients. |

CI: confidence interval; FA: Fanconi anaemia; GMR: geometric mean ratio; GMT: geometric mean titre; HPV: human papillomavirus; IBD: inflammatory bowel disease; JIA: juvenile idiopathic arthritis; NRSI: non-randomised studies of interventions; post-HSCT: allogeneic hematopoietic stem cell transplant; SLE: systemic lupus erythematosus

^a^ Risk of bias downgraded by two levels: due to very serious concerns regarding confounding.
^b^ Imprecision downgraded by two levels: due to considerably wide 95%-CI of the GMR and imprecision due to a small sample size.

^c^ Risk of bias downgraded by one level: mainly due to serious concerns regarding confounding, selection of participants into the study, deviations from intended interventions or missing outcome data.

^d^ Imprecision downgraded by one level: imprecision due to a small sample size and wide 95%-CI of the GMR.

^e^ Imprecision downgraded by one level: due to a small sample size.
^*^ Including: leukaemia, lymphoma, solid tumour participants.

# **Antibody titres: comparison 3**

**Supplement Table S16: Comparison 3 - GMT and GMR of HPV 16 at 7 months**

| **Study** | **Clinical condition, intervention, measurement unit** | **IG: N** | **IG: GMT** | **IG: dispersion measure GMT** | **CG: N** | **CG: GMT** | **CG: dispersion measure GMT** | **GMR (95%-CI)** |
| --- | --- | --- | --- | --- | --- | --- | --- | --- |
| Alter 2014 | Fanconi anaemia (FA) and other inherited bone marrow failure syndromes (IBMFS); quadrivalent HPV vaccine (probably, just Gardasil mentioned): 11 (92%); Bivalent HPV vaccine: 1 (8%) | Results were reported without summarizing GMTs across patients. Titres were compared to the general population. Authors’ conclusion: “Both FA and other IBMFS patients developed antibody levels following vaccination that were similar to those previously described in healthy women, and those levels appeared to be sustained out to 5 years after immunization. Thus, antibody responses to the HPV L1 VLP vaccine in patients with FA and other IBMFS appeared to be similar to the responses reported in the general population, implying potential efficacy against future infections with the HPV types contained in the vaccine.” | | | | | | |
| Dhar 2017 | SLE, quadrivalent HPV vaccine, mMU/mL | 19 | 3052.1 | Lower, upper CI: 2186.8, 4259.9 | 657 | 2129.5 | Lower, upper CI: 1962.7, 2310.5 | 1.43 (1.02; 2.02) |
| Esposito 2014 | Juvenile idiopathic arthritis (JIA), bivalent HPV vaccine, pseudovirion-based neutralization assay (PBNA); SEAP Reporter Gene Assay | 21 | 6834.38 | Range: 160-40960 | 21 | 12,177.48 | Range: 2560-40960 | 0.56 (NA) |
| Gomez-Lobo 2014 | Transplant recipients (Kidney), quadrivalent HPV vaccine, mMU/mL | 7 | 6872 | NR | 850 | 5168 | NR | 1.33 (NA) |
|  | Transplant recipients (Liver), quadrivalent HPV vaccine, mMU/mL | 1 | 824 | NR | 850 | 5168 | NR | 0.16 (NA) |
| Heijstek 2014 | JIA, bivalent HPV vaccine, LU/mL | 41 | 5498.72 | Upper CI 9590.79 | 41 | 13618.93 | Lower, upper CI: 8631.71, 20684.14 | 0.40 (0.20; 0.82) |
| Jacobson 2013 | Inflammatory bowel disease (IBD), 9-15 years, quadrivalent HPV vaccine, mMU/mL | 20 | 4155.1 | Lower, upper CI: 2339.5, 7379.7 | 915 | 4918.5 | Lower, upper CI: 4556.6, 5309.1 | 0.85 (0.47; 1.51)* |
|  | IBD, 16-26 years, quadrivalent HPV vaccine, mMU/mL | 13 | 3713.1 | Lower, upper CI: 1638.3, 8416.7 | 3253 | 2411.3 | Lower, upper CI: 2311.1, 2515.9 | 1.54 (0.68, 3.49) |
| Kitano 2023 | Transplant recipients (Kidney), quadrivalent HPV vaccine, mMU/mL | 4 | 35.7 | NR | 3 | 4391.6 | NR | 0.01 (NA) |
|  | Transplant recipients (Liver), quadrivalent HPV vaccine, mMU/mL | 6 | 3097.3 | NR | 3 | 4391.6 | NR | 0.71 (NA) |
| Landier 2022 | Survivors of cancer, female, 9-15 years, quadrivalent HPV vaccine, mMU/mL | 51 | 15209.7 | Lower, upper CI: 10152.4, 20267.1 | 915 | 4918.5 | Lower, upper CI: 4556.6, 5309.1 | 3.09 (1.76; 4.52) |
|  | Survivors of cancer, male, 9-15 years, quadrivalent HPV vaccine, mMU/mL | 65 | 16134.6 | Lower, upper CI: 11944.7, 20324.5 | 882 | 6056.5 | Lower, upper CI: 5601.3, 6548.7 | 2.66 (1.75; 3.66) |
|  | Survivors of cancer, female, 16-26 years, quadrivalent HPV vaccine, mMU/mL | 28 | 6107.3 | Lower, upper CI: 3149.1, 9065.5 | 3249 | 2409.2 | Lower, upper CI: 2309, 2513.8 | 2.53 (1.00; 4.09) |
|  | Survivors of cancer, male, 16-26 years, quadrivalent HPV vaccine, mMU/mL | 65 | 8740 | Lower, upper CI: 6000.6, 11479.5 | 1136 | 2403.3 | Lower, upper CI: 2243.4, 2574.6 | 3.64 (2.15; 5.21) |
|  | Survivors of cancer, female, 9-15 years, nonavalent HPV vaccine, mMU/mL | 41 | 11763.6 | Lower, upper CI: 8826.8, 14700.4 | 2405 | 7159.9 | Lower, upper CI: 6919.7, 7408.5 | 1.64 (1.12; 2.18) |
|  | Survivors of cancer, male, 9-15 years, nonavalent HPV vaccine, mMU/mL | 53 | 16419.6 | Lower, upper CI: 11743.7, 21095.5 | 1076 | 8444.9 | Lower, upper CI: 8054.2, 8854.5 | 1.94 (1.23; 2.68) |
|  | Survivors of cancer, female, 16-26 years, nonavalent HPV vaccine, mMU/mL | 23 | 11522.9 | Lower, upper CI: 6301.3, 16744.5 | 4361 | 3159 | Lower, upper CI: 3088.6, 3231.1 | 3.65 (1.61; 5.70) |
|  | Survivors of cancer, male, 16-26 years, nonavalent HPV vaccine, mMU/mL | 32 | 10770.4 | Lower, upper CI: 6127.8, 15412.9 | 899 | 3346 | Lower, upper CI: 3158.9, 3544.1 | 3.22 (1.46; 5.02) |
| Miyaji 2024 | Transplant (kidney, kidney + pancreas, liver, lung, heart) | 81 | 62.2 | Lower, upper CI: 35.3, 109.7 | 88 | 470 | Lower, upper CI: 366.6, 602.7 | 0.13 (0.07; 0.25) |
| Mok 2013 | SLE, quadrivalent HPV vaccine, mMU/mL | 39 | Median: 2791.1 | Upper CI: 5567.78 | 44 | Median: 3266.37 | Upper CI: 7238.7 | Median: 0.86 (NA) |
| Nelson 2016 | Chornic kidney disease (CKD), 9-15 years, quadrivalent HPV vaccine, mMU/mL | 10 | 2093 | NR | 915 | 4918 | NR | 0.43 (NA) |
|  | Dialysis, 9-15 years, quadrivalent HPV vaccine, mMU/mL | 6 | 5916 | NR | 915 | 4918 | NR | 1.20 (NA) |
|  | Transplant, 9-15 years, quadrivalent HPV vaccine, mMU/mL | 8 | 409 | NR | 915 | 4918 | NR | 0.08 (NA) |
|  | CKD, 16-26 years, quadrivalent HPV vaccine, mMU/mL | 8 | 2871 | NR | 3249 | 2409 | NR | 1.19 (NA) |
|  | Dialysis, 16-26 years, quadrivalent HPV vaccine, mMU/mL | 3 | 1340 | NR | 3249 | 2409 | NR | 0.56 (NA) |
|  | Transplant, 16-26 years, quadrivalent HPV vaccine, mMU/mL | 13 | 137 | NR | 3249 | 2409 | NR | 0.06 (NA) |
| Stratton 2020 | Allogeneic hematopoietic stem cell transplant (post-HSCT), quadrivalent HPV vaccine, on immunosuppresants, EU/mL | 23 | 1144.9 | Lower, upper CI: 303.3, 4322.3 | 20 | 2368.8 | Lower, upper CI: 1447.3, 3876.9 | 0.48 (0.11; 2.17) |
|  | Allogeneic hematopoietic stem cell transplant (post-HSCT), quadrivalent HPV vaccine, not on immunosuppresants, EU/mL | 21 | 2667.2 | Lower, upper CI: 1404.5, 5064.9 | 20 | 2368.8 | Lower, upper CI: 1447.3, 3876.9 | 1.13 (0.44; 2.90) |

CG: control group; CI: confidence interval; CKD: chronic kidney disease; EU/mL: ELISA-Unit per millilitre; FA: Fanconi anaemia; GMR: geometric mean/median ratio; GMT: geometric mean titre; HPV: human papillomavirus; IBD: inflammatory bowel disease; IBMFS: inherited bone marrow failure syndromes; IG: intervention group; JIA: juvenile idiopathic arthritis; LU/mL: Luminex Units/ml; mMU/mL: milli-Merck Units per millilitre; N: number; NA: not applicable; NR: not reported; PBNA: pseudoviron-based neutralization assay; post-HSCT: allogeneic hematopoietic stem cell transplant; SEAP: secreted embryonic alkaline phosphatase; SLE: systemic lupus erythematosus; VLP: virus-like particles.

*Due to rounding, the GMR appears slightly different in the corresponding meta-analyses.

**Supplement Table S17: Comparison 3 - GMT and GMT of HPV 18 at 7 months**

| **Study** | **Clinical condition, intervention, measurement unit** | **IG: N** | **IG: GMT** | **IG: dispersion measure GMT** | **CG: N** | **CG: GMT** | **CG: dispersion measure GMT** | **GMR (95%-CI)** |
| --- | --- | --- | --- | --- | --- | --- | --- | --- |
| Alter 2014 | Fanconi anaemia (FA) and other inherited bone marrow failure syndromes (IBMFS); Quadrivalent HPV vaccine (probably, just Gardasil mentioned): 11 (92%); Bivalent HPV vaccine: 1 (8%) | Results were reported as case series without summarizing GMTs across patients. Titres were compared to the general population. Authors’ conclusion: “Both FA and other IBMFS patients developed antibody levels following vaccination that were similar to those previously described in healthy women, and those levels appeared to be sustained out to 5 years after immunization. Thus, antibody responses to the HPV L1 VLP vaccine in patients with FA and other IBMFS appeared to be similar to the responses reported in the general population, implying potential efficacy against future infections with the HPV types contained in the vaccine.” | | | | | | |
| Dhar 2017 | SLE, quadrivalent HPV vaccine, mMU/mL | 27 | 567.7 | Lower, upper CI: 404.2, 797.4 | 722 | 324.6 | Lower, upper CI: 297.6, 354 | 1.75 (1.23;2.48) |
| Esposito 2014 | Juvenile idiopathic arthritis (JIA), bivalent HPV vaccine, pseudovirion-based neutralization assay (PBNA); SEAP Reporter Gene Assay | 21 | 5120 | Range: 640-20480 | 21 | 6347.86 | Range: 640-40960 | 0.81 (NA) |
| Gomez-Lobo 2014 | Transplant recipients (Kidney), quadrivalent HPV vaccine, mMU/mL | 7 | 1619 | NR | 850 | 1064 | NR | 1.52 (NA) |
|  | Transplant recipients (Liver), quadrivalent HPV vaccine, mMU/mL | 1 | 1616 | NR | 850 | 1064 | NR | 1.52 (NA) |
| Heijstek 2014 | JIA, bivalent HPV vaccine, LU/mL | 41 | 2926.83 | Upper CI: 4745.42 | 41 | 5589.43 | Lower, upper CI: 3584.52, 8778 | 0.52 (0.27; 0.01) |
| Jacobson 2013 | Inflammatory bowel disease (IBD), 9-15 years, quadrivalent HPV vaccine, mMU/mL | 20 | 1193.8 | Lower, upper CI: 571.1, 2495.5 | 922 | 1042.6 | Lower, upper CI: 967.6, 1123.3 | 1.15. (0.55; 2.40) |
|  | IBD, 16-26 years, quadrivalent HPV vaccine, mMU/mL | 13 | 515.5 | Lower, upper CI: 195, 1362.8 | 3571 | 475.6 | Lower, upper CI: 459.2, 492.6 | 1.08 (0.41, 2.87) |
| Kitano 2023 | Transplant recipients (Kidney), quadrivalent HPV vaccine, mMU/mL | 4 | 42.4 | NR | 3 | 902.6 | NR | 0.05 (NA) |
|  | Transplant recipients (Liver), quadrivalent HPV vaccine, mMU/mL | 6 | 835.7 | NR | 3 | 902.6 | NR | 0.93 (NA) |
| Landier 2022 | Survivors of cancer, female, 9-15, quadrivalent HPV vaccine, mMU/mL | 54 | 2638.3 | Lower, upper CI: 1792.5, 3484.1 | 922 | 1042.6 | Lower, upper CI: 967.6, 1123.3 | 2.53 (1.48; 3.66) |
|  | Survivors of cancer, male, 9-15, quadrivalent HPV vaccine, mMU/mL | 66 | 3472.2 | Lower, upper CI: 2407.9, 4536.5 | 887 | 1357.4 | Lower, upper CI: 1249.4, 1474.7 | 2.56 (1.52; 3.68) |
|  | Survivors of cancer, female, 16-26, quadrivalent HPV vaccine, mMU/mL | 30 | 1009.6 | Lower, upper CI: 582.9, 1436.3 | 3566 | 475.2 | Lower, upper CI: 458.8, 492.1 | 2.12 (1.00; 3.26) |
|  | Survivors of cancer, male, 16-26, quadrivalent HPV vaccine, mMU/mL | 68 | 1920 | Lower, upper CI: 1210.2, 2629.9 | 1175 | 402.6 | Lower, upper CI: 374.6, 432.7 | 4.77 (2.48; 7.18) |
|  | Survivors of cancer, female, 9-15, nonavalent HPV vaccine, mMU/mL | 41 | 3457.2 | Lower, upper CI: 2545, 4369.4 | 2420 | 2085.5 | Lower, upper CI: 2002.2, 2172.3 | 1.66 (1.10; 2.23) |
|  | Survivors of cancer, male, 9-15, nonavalent HPV vaccine, mMU/mL | 50 | 5559.8 | Lower, upper CI: 4081.9, 7037.7 | 1074 | 2620.4 | Lower, upper CI: 2474.3, 2775.2 | 2.12 (1.39; 2.89) |
|  | Survivors of cancer, female, 16-26, nonavalent HPV vaccine, mMU/mL | 28 | 3483.3 | Lower, upper CI: 408.6, 6557.9 | 4884 | 809.9 | Lower, upper CI: 789.2, 831.1 | 4.30 (0.00; 9.05) |
|  | Survivors of cancer, male, 16-26, nonavalent HPV vaccine, mMU/mL | 32 | 3013.4 | Lower, upper CI: 1685.1, 4341.6 | 906 | 808.2 | Lower, upper CI: 754.9, 865.4 | 3.73 (1.65; 5.89) |
| Miyaji 2024 | Transplant (kidney, kidney + pancreas, liver, lung, heart) | 67 | 18 | Lower, upper CI: 9.6, 33.8 | 96 | 135.9 | Lower, upper CI: 101.9, 181.1 | 0.13 (0.07; 0.26) |
| Mok 2013 | SLE, quadrivalent HPV vaccine, mMU/mL | 38 | Median: 562.4 | Upper limit: 1474.4 | 40 | Median: 847.7 | Upper limit: 2120.4 | Median: 0.66 (NA) |
| Nelson 2016 | Chronic kidney disease (CKD), 9-15 years, quadrivalent HPV vaccine, mMU/mL | 10 | 317 | NR | 917 | 1043 | NR | 0.30 (NA) |
|  | Dialysis, 9-15 years, quadrivalent HPV vaccine, mMU/mL | 6 | 1032 | NR | 917 | 1043 | NR | 0.99 (NA) |
|  | Transplant, 9-15 years, quadrivalent HPV vaccine, mMU/mL | 8 | 61 | NR | 917 | 1043 | NR | 0.06 (NA) |
|  | CKD, 16-26 years, quadrivalent HPV vaccine, mMU/mL | 8 | 429 | NR | 3329 | 475 | NR | 0.90 (NA) |
|  | Dialysis, 16-26 years, quadrivalent HPV vaccine, mMU/mL | 3 | 199 | NR | 3329 | 475 | NR | 0.42 (NA) |
|  | Transplant, 16-26 years, quadrivalent HPV vaccine, mMU/mL | 13 | 36 | NR | 3329 | 475 | NR | 0.08 (NA) |
| Stratton 2020 | Allogeneic hematopoietic stem cell transplant (post-HSCT), quadrivalent HPV vaccine, on immunosuppresants, EU/mL | 23 | 366.6 | Lower, upper CI: 120.6, 1115.1 | 20 | 759.5 | Lower, upper CI: 555.6, 1038.2 | 0.48 (0.15; 1.60) |
|  | Allogeneic hematopoietic stem cell transplant (post-HSCT), quadrivalent HPV vaccine, not on immunosuppresants, EU/mL | 21 | 698.3 | Lower, upper CI: 333.3, 1463.3 | 20 | 759.5 | Lower, upper CI: 555.6, 1038.2 | 0.92 (0.39; 2.18) |

CG: control group; CI: confidence interval; CKD: chronic kidney disease; EU/mL: ELISA-Unit per millilitre; FA: Fanconi anaemia; GMR: geometric mean/median ratio; GMT: geometric mean/median titre; HPV: human papillomavirus; IBD: inflammatory bowel disease; IBMFS: inherited bone marrow failure syndromes; IG: intervention group; JIA: juvenile idiopathic arthritis; LU/mL: Luminex Units/ml; mMU/mL: milli-Merck Units per millilitre; N: number; NA: not applicable; NR: not reported; PBNA: pseudoviron-based neutralization assay; post-HSCT: allogeneic hematopoietic stem cell transplant; SEAP: secreted embryonic alkaline phosphatase; SLE: systemic lupus erythematosus; VLP: virus-like particle

**Supplement Table S18: Comparison 3 - GMT and GMR of HPV 16 at 12 months and more**

| **Study** | **Clinical condition, intervention, measurement unit** | **IG: N** | **IG: GMT** | **IG: dispersion measure GMT** | **CG: N** | **CG: GMT** | **CG: dispersion measure GMT** | **GMR (95%-CI)** |
| --- | --- | --- | --- | --- | --- | --- | --- | --- |
| Heijstek 2014 | Juvenile idiopathic arthritis (JIA), bivalent HPV vaccine, LU/mL | 43 | 3452.69 | Upper CI: 6202.05 | 44 | 7896.42 | Lower, upper CI: 5635.25, 11270.49 | 0.44 (0.22; 0.87) |
| Mok 2013 | Systemic lupus erythematosus (SLE), quadrivalent HPV vaccine, mMU/mL | 39 | Median: 1065.3 | Upper limit: 2102.8 | 44 | Median: 1174.8 | Upper limit: 2202.56 | Median: 0.91 (NA) |
| Nelson 2016 | Chronic kidney disease (CKD), 9-15 years, quadrivalent HPV vaccine, mMU/mL | 11 | 1759 | NR | 214 | 944 | NR | 1.86 (NA) |
|  | Dialysis, 9-15 years, quadrivalent HPV vaccine, mMU/mL | 2 | 417 | NR | 214 | 944 | NR | 0.44 (NA) |
|  | Transplant, 9-15 years, quadrivalent HPV vaccine, mMU/mL | 5 | 156 | NR | 214 | 944 | NR | 0.17 (NA) |
|  | Chronic kidney disease (CKD), 16-26 years, quadrivalent HPV vaccine, mMU/mL | 10 | 1104 | NR | 2788 | 442 | NR | 2.50 (NA) |
|  | Dialysis, 16-26 years, quadrivalent HPV vaccine, mMU/mL | 1 | 352 | NR | 2721 | 442 | NR | 0.80 (NA) |
|  | Transplant, 16-26 years, quadrivalent HPV vaccine, mMU/mL | 3 | 133 | NR | 2721 | 442 | NR | 0.30 (NA) |
| Sauter 2021 | Fanconi anaemia (FA), HSCT, quadrivalent HPV vaccine (primarily), IU/mL | 37 | 44.97 | Lower, upper CI: 17.28, 117 | 21 | 120.8 | Lower, upper CI: 33.87, 431.1 | 0.37 (0.05; 2.97) |
|  | Fanconi anaemia (FA), no HSCT, quadrivalent HPV vaccine (primarily), IU/mL | 23 | 117.6 | Lower, upper CI: 38.26, 361.2 | 21 | 120.8 | Lower, upper CI: 33.87, 431.1 | 0.97 (0.11; 8.42) |
| Stratton 2020 | Allogeneic hematopoietic stem cell transplant (post-HSCT), quadrivalent HPV vaccine, on immunosuppresants, EU/mL | 23 | 361.6 | Lower, upper CI: 138.7, 942.8 | 20 | 667.2 | Lower, upper CI: 377.5, 1179.4 | 0.54 (0.16; 1.90)* |
|  | Allogeneic hematopoietic stem cell transplant (post-HSCT), quadrivalent HPV vaccine, not on immunosuppresants, EU/mL | 21 | 756.2 | Lower, upper CI: 449.9, 1271 | 20 | 667.2 | Lower, upper CI: 377.5, 1179.4 | 1.13 (0.44; 2.96)* |

CG: control group; CI: confidence interval; CKD: chronic kidney disease; EU/mL: ELISA-Unit per millilitre; FA: Fanconi anaemia; GMR: geometric mean/median ratio; GMT: geometric mean/median titre; HPV: human papillomavirus; HSCT: hematopoietic stem cell transplant; IBD: IG: intervention group; IU/mL: international unit per millilitre; JIA: juvenile idiopathic arthritis; LU/mL: Luminex Units/ml; mMU/mL: milli-Merck Units per millilitre; N: number; NA: not applicable; NR: not reported; post-HSCT: allogeneic hematopoietic cell transplant; SLE: systemic lupus erythematosus.
*Due to rounding, 95% CIs appear slightly different in the corresponding meta-analyses.

| **Study** | **Clinical condition, intervention, measurement unit** | **IG: N** | **IG: GMT** | **IG: dispersion measure GMT** | **CG: N** | **CG: GMT** | **CG: dispersion measure GMT** | **GMR (95%-CI)** |
| --- | --- | --- | --- | --- | --- | --- | --- | --- |
| Heijstek 2014 | Juvenile idiopathic arthritis (JIA), bivalent HPV vaccine, LU/mL | 43 | 1428.57 | Upper CI: 2632.65 | 44 | 2285.71 | Lower, upper CI: 1591.83, 3163.26 | 0.63 (0.31; 1.25) |
| Mok 2013 | Systemic lupus erythematosus (SLE), quadrivalent HPV vaccine, mMU/mL | 38 | Median: 163.7 | Upper limit: 374.5 | 40 | Median: 166.9 | Upper limit: 342.2 | Median: 0.98 (NA) |
| Nelson 2016 | Chronic kidney disease (CKD), 9-15 years, quadrivalent HPV vaccine, mMU/mL | 11 | 193 | NR | 214 | 138 | NR | 1.40 (NA) |
|  | Dialysis, 9-15 years, quadrivalent HPV vaccine, mMU/mL | 2 | 34 | NR | 214 | 138 | NR | 0.25 (NA) |
|  | Transplant, 9-15 years, quadrivalent HPV vaccine, mMU/mL | 5 | 38 | NR | 214 | 138 | NR | 0.28 (NA) |
|  | CKD, 16-26 years, quadrivalent HPV vaccine, mMU/mL | 10 | 71 | NR | 2788 | 51 | NR | 1.39 (NA) |
|  | Dialysis, 16-26 years, quadrivalent HPV vaccine, mMU/mL | 1 | 5 | NR | 2788 | 51 | NR | 0.10 (NA) |
|  | Transplant, 16-26 years, quadrivalent HPV vaccine, mMU/mL | 3 | 25 | NR | 2788 | 51 | NR | 0.49 (NA) |
| Sauter 2021 | Fanconi anaemia (FA), HSCT, quadrivalent HPV vaccine (primarily), IU/mL | 37 | 9.33 | Lower, upper CI: 3.55, 24.52 | 21 | 35.48 | Lower, upper CI: 11.68, 107.7 | 0.26 (0.04; 1.72) |
|  | Fanconi anaemia (FA), no HSCT, quadrivalent HPV vaccine (primarily), IU/mL | 23 | 44.05 | Lower, upper CI: 15.56, 124.7 | 21 | 35.48 | Lower, upper CI: 11.68, 107.7 | 1.24 (0.18; 8.44) |
| Stratton 2020 | Allogeneic hematopoietic stem cell transplant (post-HSCT), quadrivalent HPV vaccine, on immunosuppresants, EU/mL | 23 | 116.6 | Lower, upper CI: 47, 289.7 | 20 | 196.3 | Lower, upper CI: 134.7, 286 | 0.59 (0.21; 1.70) |
|  | Allogeneic hematopoietic stem cell transplant (post-HSCT), quadrivalent HPV vaccine, not on immunosuppresants, EU/mL | 21 | 222.7 | Lower, upper CI: 115.6, 429 | 20 | 196.3 | Lower, upper CI: 134.7, 286 | 1.14 (0.49; 2.64) |

**Supplement Table S19: Comparison 3 - GMT and GMR of HPV 18 at 12 months and more**

CG: control group; CI: confidence interval; CKD: chronic kidney disease; EU/mL: ELISA-Unit per millilitre; FA: Fanconi anaemia; GMR: geometric mean ratio; GMT: geometric mean titre; HPV: human papillomavirus; HSCT: hematopoietic stem cell transplant; IG: intervention group; IU/mL: international unit per millilitre; JIA: juvenile idiopathic arthritis; LU/mL: Luminex Units/ml; mMU/mL: milli-Merck Units per millilitre; N: number; NA: not applicable; NR: not reported; post-HSCT: allogeneic hematopoietic cell transplant; SLE: systemic lupus erythematosus

# **Additional study characteristics: single-arm studies**

**Supplement Table S20: Study characteristics (single-arm studies)**

| **Study** | **Country** | **Funding** | **DoI** | **Clinical condition** | **N Intervention group** | **Age (years)** | **Sex (%)** | **Intervention** | **Immunosuppressive medication at baseline** | **HPV vaccination status at baseline** |
| --- | --- | --- | --- | --- | --- | --- | --- | --- | --- | --- |
| Boey 2021 | Belgium | Industry | Interests declared | Transplant recipients | 171 | Median, range:46 (19-55) | Female (31)  Male (69) | Nonavalent HPV vaccine | **All transplant recipients:**   - One immunosuppressive agent: 2 (1.2%) - Two immunosuppressive agents: 100 (58.5%) - Three immunosuppressive agents: 69 (40.4%) - Methylprednisolone: 83 (48.5%) - Azathioprine: 26 (15.2%) - Cyclosporine: 13 (7.6%) - Tacrolimus: 125 (73.1%) - Mycophenolate mofetil: 154 (90.1%) - Sirolimus or Everolimus: 7 (4.1%) | Study excluded participants with a history of HPV vaccination. |
| Kumar 2013 | Canada | Mixed | Interests declared | Transplant recipients | 47 | Median, range: 25.9 (18-35) | Female (66)  Male (34) | Quadrivalent HPV vaccine | - Prednisone: 36 (76.6%) - Calcineurin-inhibitor: 43 (91.5%) - Mycophenolate mofetil: 42 (87.5%) - Sirolimus: 3 (6.4%) | NR |
| Liu 2018 | Canada | Industry | Interests declared | History of immune mediated diseases^*^ | NA | Range: 12-17 | Female (100) | Quadrivalent HPV vaccine | NR | Study excluded girls who received HPV vaccination before program eligibility and those whose vaccination records were either unavailable or inactive |
| MacIntyre 2016 and 2019 | Australia | Mixed | Interests declared | Immunocompromised children^§^ | 59 | Mean, range: 12.3 (5–18) | Female (44)  Male (56) | Quadrivalent HPV vaccine | - One immunosuppressive agent: 13 (22.0%) - More than one immunosuppressive agent: 24 (40.7%) | Study excluded participants with a history of HPV vaccination. |
| Praditpornsilpa 2016 | Thailand | Industry | Interests declared | Chronic kidney disease (CKD; Stage 4-5) | 60 | Mean, SD 21.5 6 (4.6) | Female (47)  Male (53) | Quadrivalent HPV vaccine | Study excluded people receiving immunosuppressive agents. | Study excluded participants with a history of HPV vaccination. |
| Soybilgic 2013 | USA | Industry | Interests declared | Systemic lupus erythematosus (SLE) | 27 | Mean: 20.5 | Female (100) | Quadrivalent HPV vaccine | - Hydroxychloroquine: 27 (100%) - Prednisone: 16 (59.2%) - Mycophenolate mofetil: 9 (33.3%) - Azathioprine: 9 (33.3%) - Methotrexate: 6 (22.2%)   In the past, 14.8% and 18.5% had received cyclophosphamide and rituximab, respectively. | Study excluded participants with a history of HPV vaccination. |

CKD: chronic kidney disease; DoI: declaration of interest; HPV: human papillomavirus; NA: not applicable; NR: not reported; SD: standard deviation; SLE: systemic lupus erythematosus
* Including: asthma, anaphylaxis and other atopic manifestations

§ Including: hematopoietic stem cell transplant (HSCT), liver transplantation, kidney transplantation, juvenile idiopathic

arthritis, inflammatory bowel disease

# **Sensitivity analyses: comparisons 2-3**

**Supplement Table S21: Sensitivity analysis**

| **Outcome/characteristics for sensitivity analyses** | **Estimate** | **Heterogeneity (I^2^)** | **Studies** | **Participants** |
| --- | --- | --- | --- | --- |
| **Seropositivity of HPV 16 at 7 months (comparison 2), Dialyses vs. CKD** | | | | |
| NRSI: Random-effect model (primary analysis) | **RR 0**.**96** (0.87 to 1.05) | 0% | 2 | 54 |
| NRSI: Fixed-effect model | **RR 0**.**96** (0.87 to 1.05) | 0% | 2 | 54 |
| **Seropositivity of HPV 18 at 7 months (comparison 2), Dialyses vs. CKD** | | | | |
| NRSI: Random-effect model (primary analysis) | **RR 0**.**98** (0.81 to 1.17) | 0% | 2 | 54 |
| NRSI: Fixed-effect model | **RR 0**.**98** (0.81 to 1.17) | 0% | 2 | 54 |
| **Seropositivity of HPV 16 at 7 months (comparison 2), Transplant vs. CKD** | | | | |
| NRSI: Random-effect model (primary analysis) | **RR 0**.**94** (0.86 to 1.03) | 0% | 2 | 78 |
| NRSI: Fixed-effect model | **RR 0**.**94** (0.86 to 1.03) | 0% | 2 | 78 |
| **Seropositivity of HPV 18 at 7 months (comparison 2), Transplant vs. CKD** | | | | |
| NRSI: Random-effect model (primary analysis) | **RR 0**.**77** (0.63 to 0.94) | 7% | 2 | 78 |
| NRSI: Fixed-effect model | **RR 0**.**78** (0.65 to 0.93) | 7% | 2 | 78 |
| **Seropositivity of HPV 16 at 7 months (comparison 3), JIA** | | | | |
| NRSI: Random-effect model (primary analysis) | **RR 1.00** (0.96 to 1.04) | 0% | 2 | 124 |
| NRSI: Fixed-effect model | **RR 1.00** (0.96 to 1.04) | 0% | 2 | 124 |
| **Seropositivity of HPV 18 at 7 months (comparison 3), JIA** | | | | |
| NRSI: Random-effect model (primary analysis) | **RR 1.00** (0.96 to 1.04) | 0% | 2 | 124 |
| NRSI: Fixed-effect model | **RR 1.00** (0.96 to 1.04) | 0% | 2 | 124 |
| **Seropositivity of HPV 16 at 7 months (comparison 3), SLE** | | | | |
| NRSI: Random-effect model (primary analysis) | **RR 0.99** (0.95 to 1.03) | 81% | 3 | 898 |
| NRSI: Fixed-effect model | **RR 1.01** (1.00 to 1.02) | 81% | 3 | 898 |
| NRSI: Random-effect model, exclusion of critical risk of bias* | **RR 0.97** (0.94 to 1.00) | 0% | 2 | 222 |
| **Seropositivity of HPV 18 at 7 months (comparison 3), SLE** | | | | |
| NRSI: Random-effect model (primary analysis) | **RR 0.94** (0.83 to 1.06) | 92% | 3 | 966 |
| NRSI: Fixed-effect model | **RR 1.03** (1.01 to 1.04) | 92% | 3 | 966 |
| NRSI: Random-effect model, exclusion of critical risk of bias* | **RR 0.90** (0.86 to 0.95) | 0% | 2 | 217 |

CKD: chronic kidney disease; HPV: human papillomavirus; JIA: juvenile idiopathic arthritis; NRSI: non-randomised studies of interventions; RR: risk ratio; SLE: systemic lupus erythematosus
* Exclusion of the study of Dhar 2017

# **Additional analyses: comparison 3**

1.
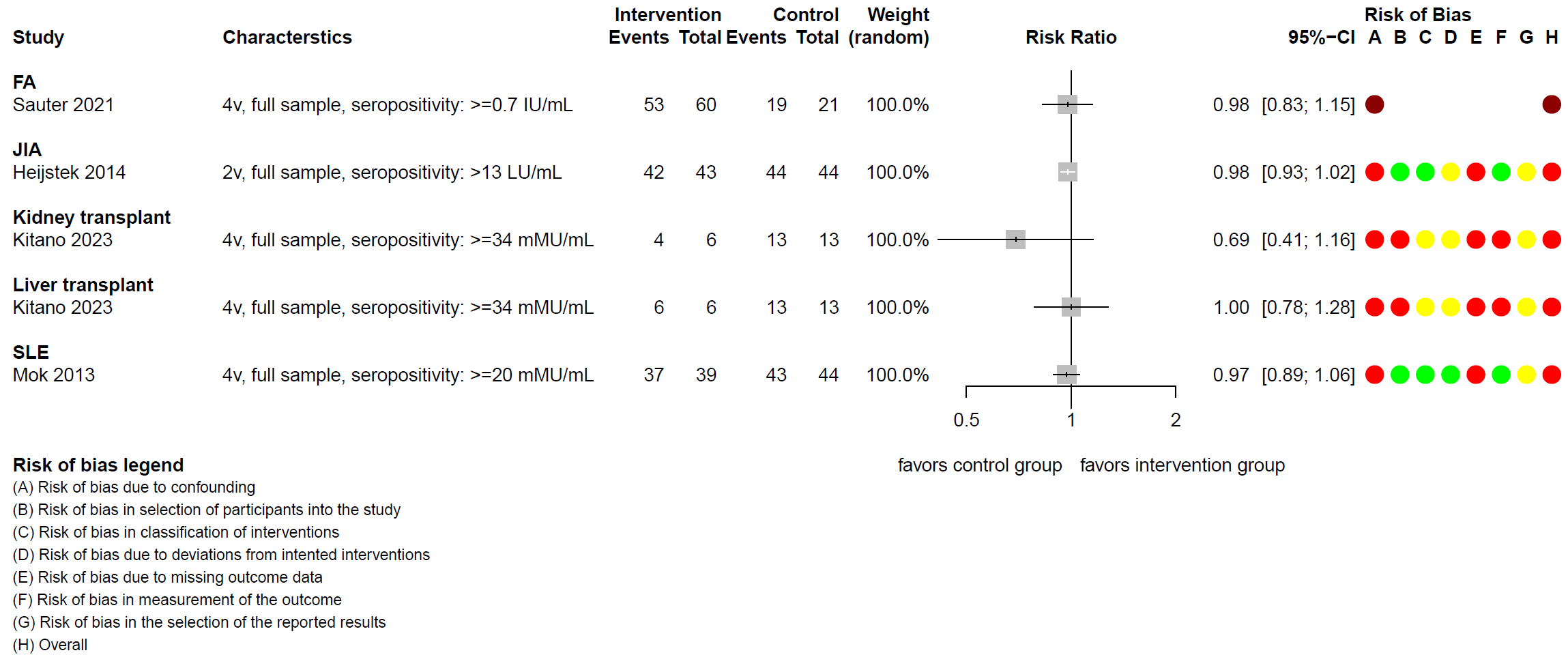
**HPV 16**
2. **HPV 18**


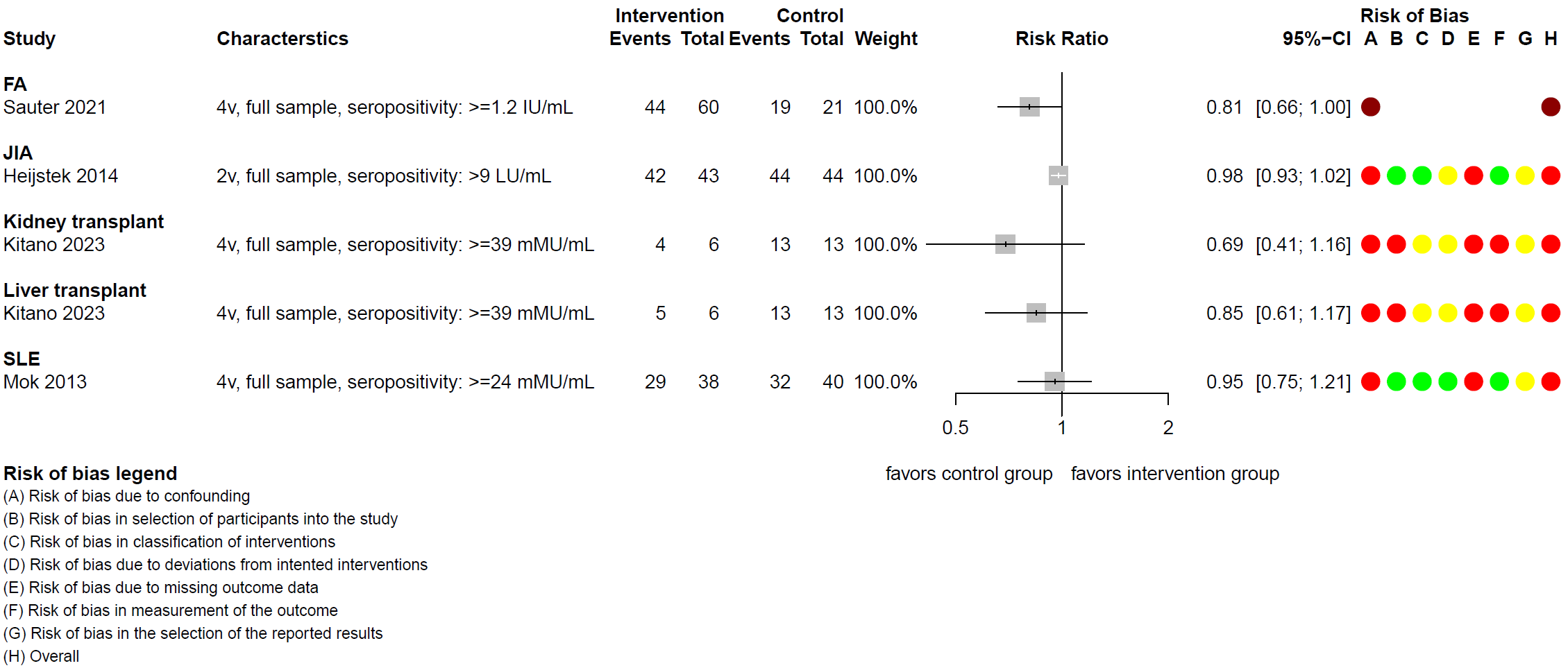


**Supplement Figure S1:** **Seropositivity of HPV 16 (A) and 18 (B) at 12 months and more (comparison 3).** Meta-analyses are presented for each immunocompromised group, alongside the risk of bias assessment. CI: confidence interval; FA: Fanconi anaemia; IU/mL: international unit per millilitre; JIA: juvenile idiopathic arthritis; LU/mL: Luminex Units per millilitre; mMU/mL: milli-Merck Units per millilitre; SLE: systemic lupus erythematosus; 2v: bivalent; 4v: quadrivalent; 9v: nonavalent

1. **HPV 16**


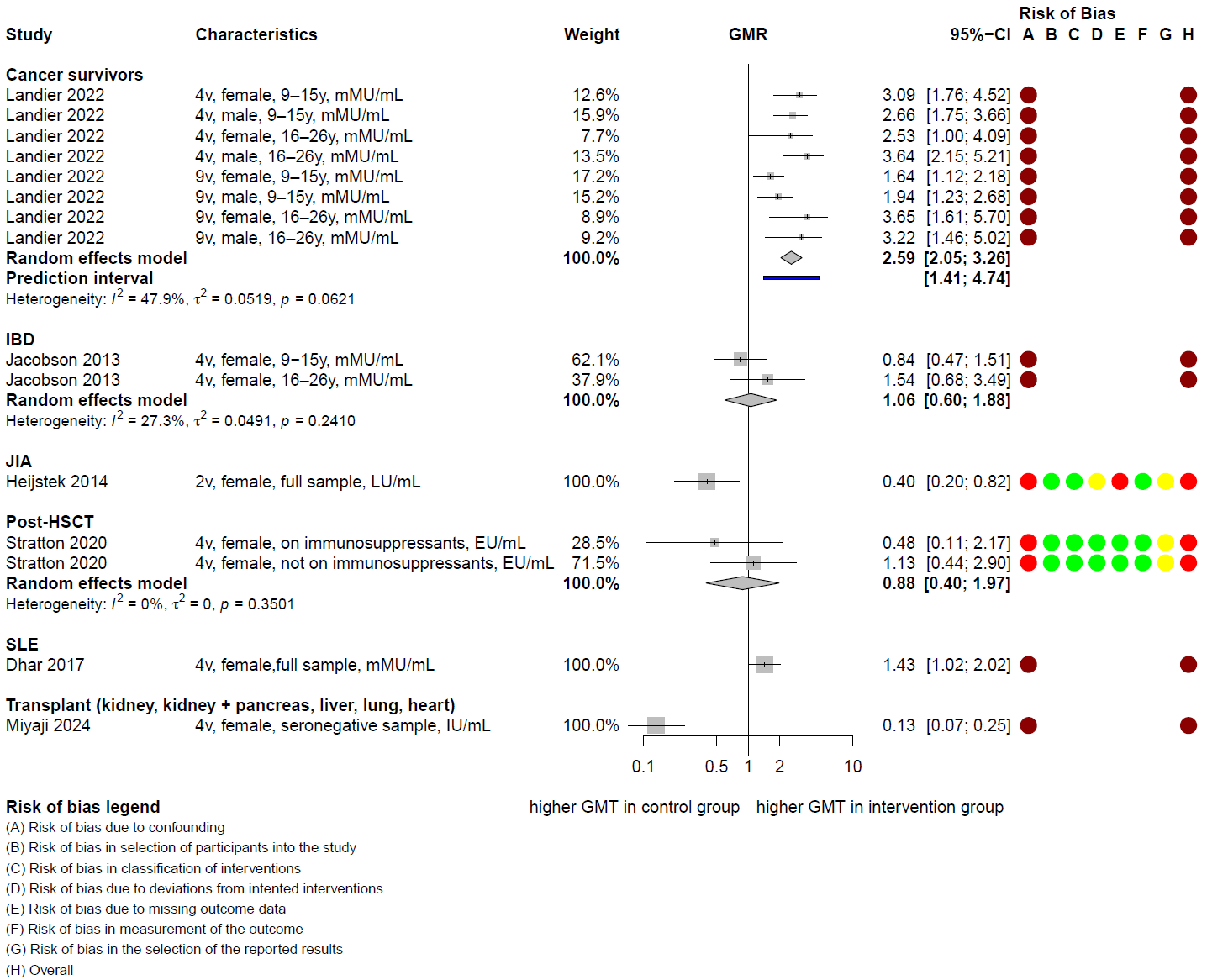


1. **HPV 18**


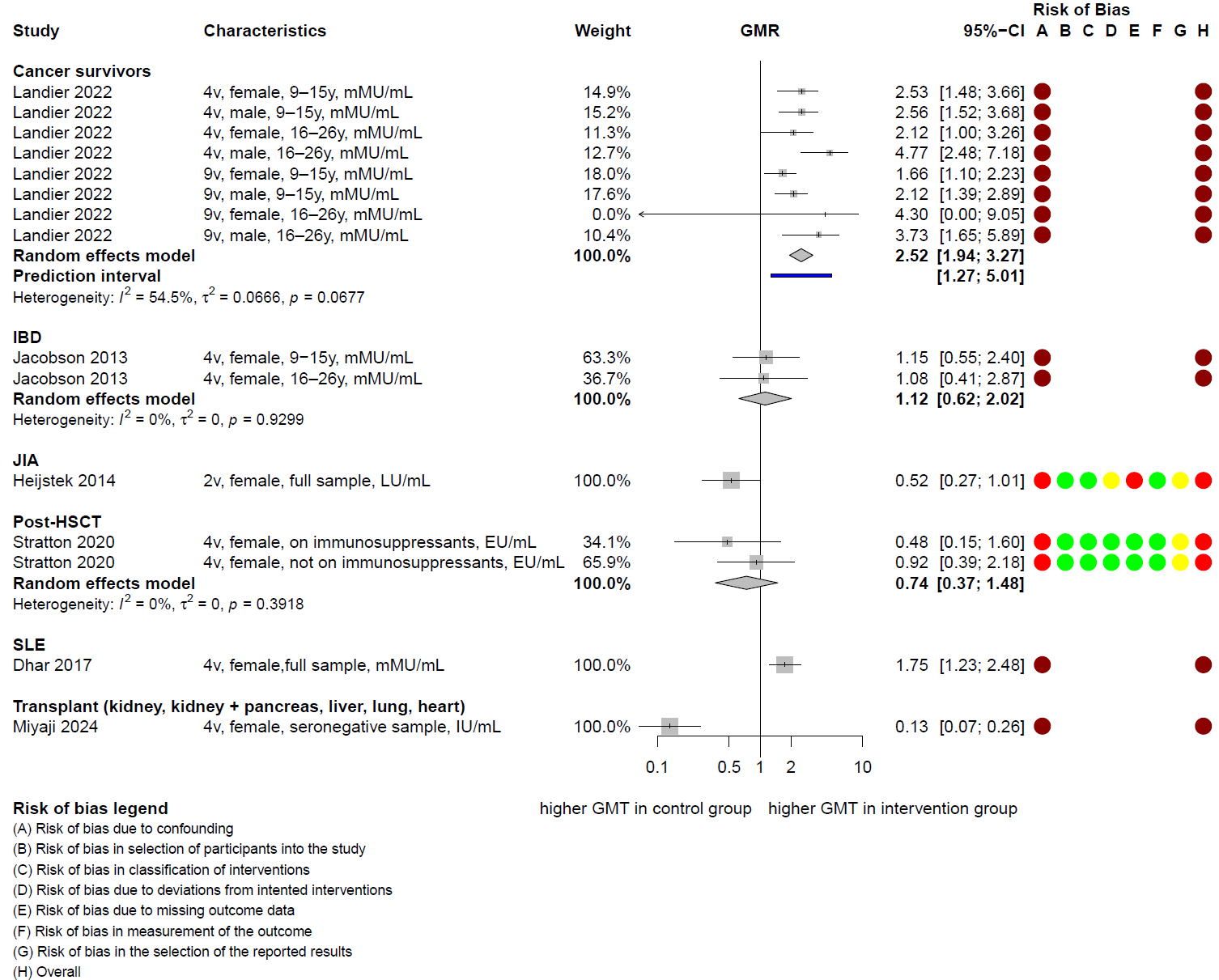
 **Supplement Figure S2: GMR of HPV 16 (A) and 18 (B) at 7 months (comparison 3)**. Meta-analyses are presented for each immunocompromised group, alongside the risk of bias assessment. CI: confidence interval; EU/mL: ELISA-Unit per millilitre; GMR: geometric mean ratio; GMT: geometric mean titre; IBD: inflammatory bowel disease; IU/mL: international unit per millilitre; JIA: juvenile idiopathic; arthritis; LU/mL: Luminex Units per millilitre; mMU/mL: milli-Merck Units per millilitre; post-HSCT: allogeneic hematopoietic stem cell transplant; SLE: systemic lupus erythematosus; y: years; 2v: bivalent; 4v: quadrivalent; 9v: nonavalent Note: The 95% CIs reported in Landier (2022) appear asymmetric, likely due to Fieller’s method used in the primary study (see <https://doi.org/10.1111/biom.13363> for details)

1. **HPV 16**


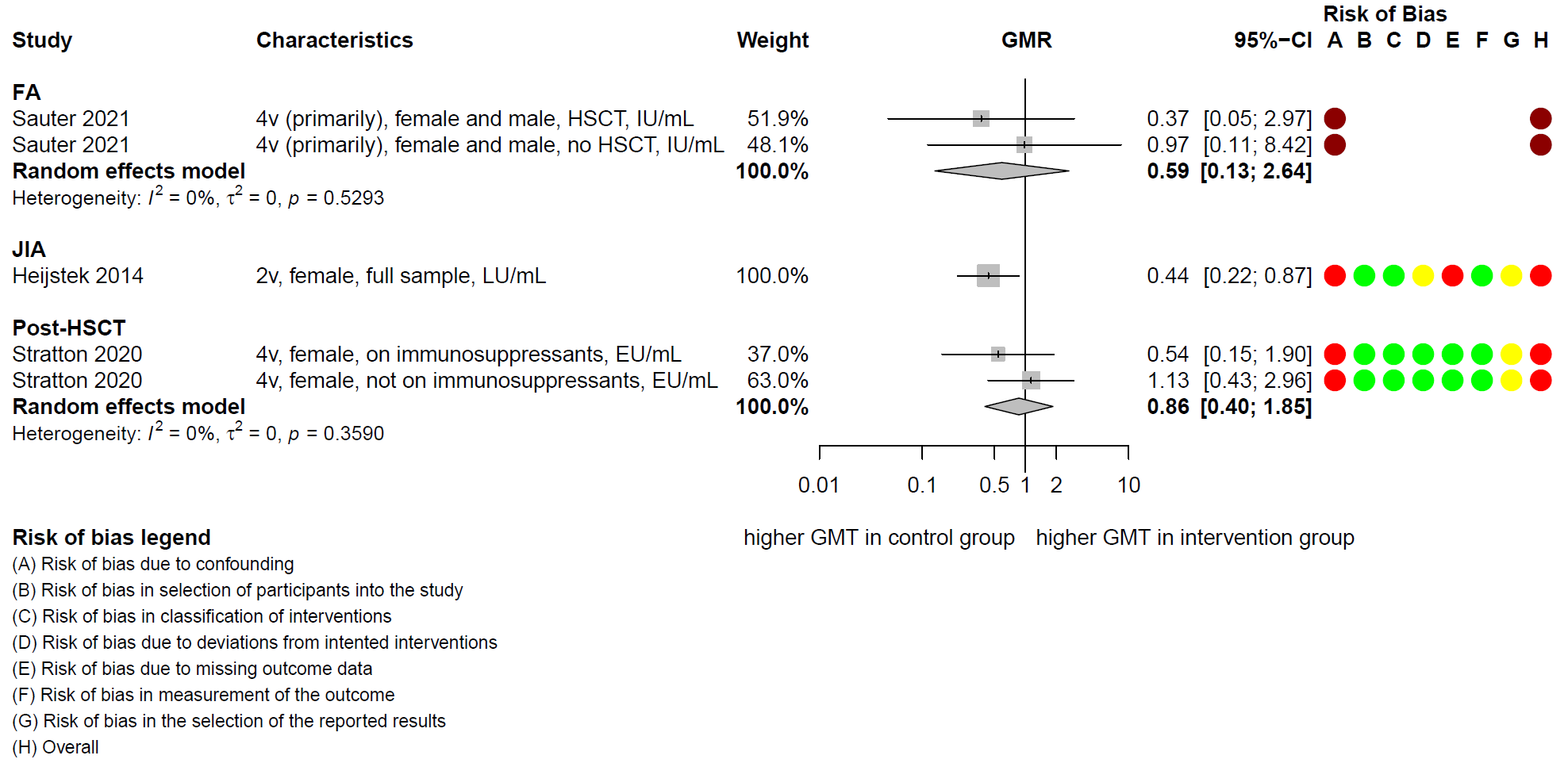


1. **HPV 18**
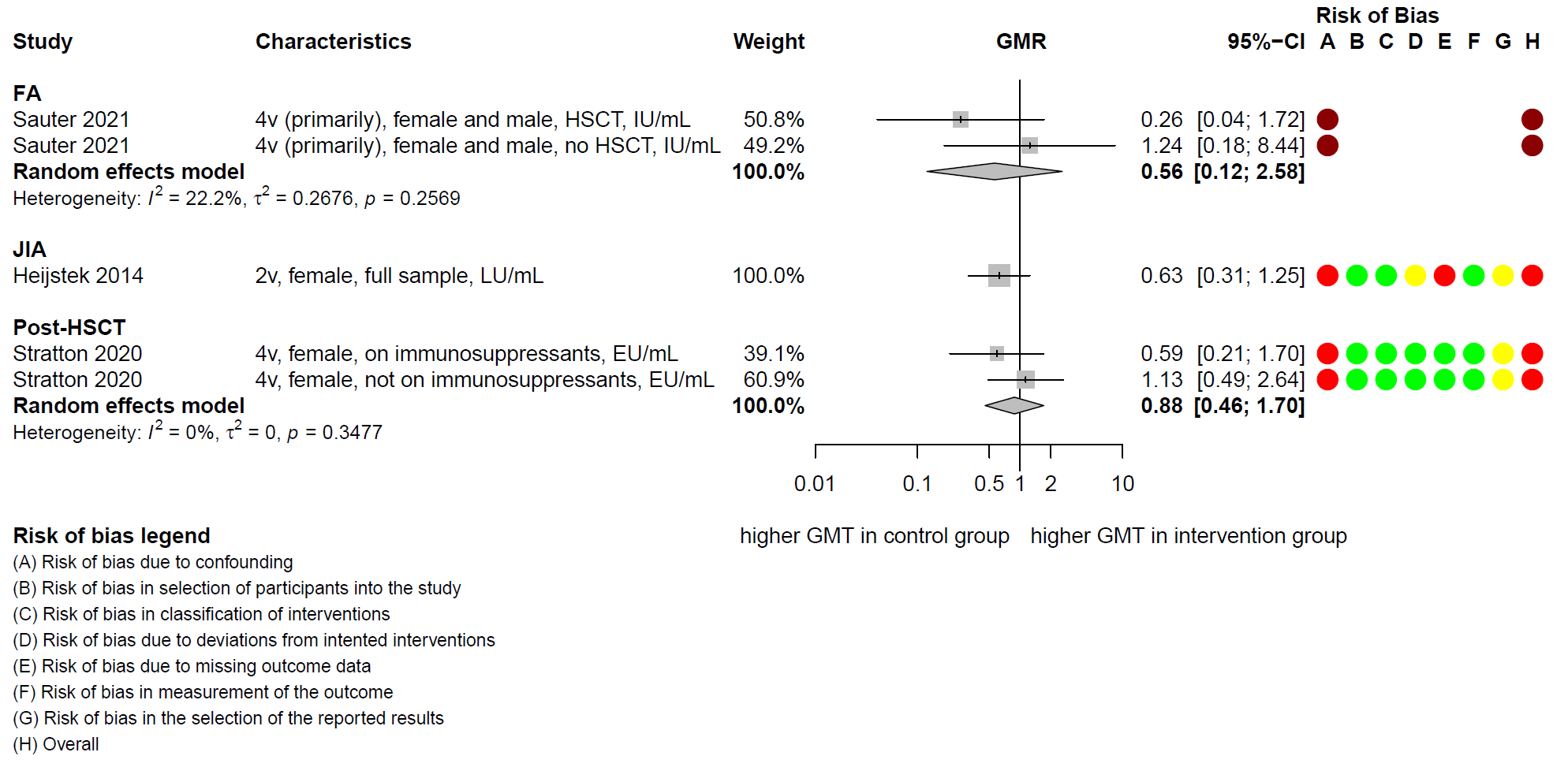


**Supplement Figure S3: GMR of HPV 16 (A) and 18 (B) at 12 months and more (comparison 3).** Meta-analyses are presented for each immunocompromised group, alongside the risk of bias assessment. CI: confidence interval; EU/mL: ELISA-Unit per millilitre; GMR: geometric mean ratio; GMT: geometric mean titre; HSCT: hematopoietic stem cell transplant; IU/mL: international unit per millilitre; JIA: juvenile idiopathic arthritis; LU/mL: Luminex Units per millilitre; post-HSCT: allogeneic hematopoietic stem cell transplant; 2v: bivalent; 4v: quadrivalent

1. For the purpose of this review, the abbreviation “NRSI” is used interchangeably for non-randomised studies of interventions, observational and single-arm studies. [↑](#footnote-ref-1)
2. Detection of a new HPV infection (defined as the presence of type-specific HPV DNA) after vaccination. [↑](#footnote-ref-2)
3. Persistent HPV infection defined as the presence of type-specific HPV DNA on repeated clinical biological samples over a period of at least six months after vaccination. [↑](#footnote-ref-3)
